# One‐Step Maleimide‐Based Dual Functionalization of Protein N‐Termini

**DOI:** 10.1002/anie.202417134

**Published:** 2024-11-27

**Authors:** Kengo Hanaya, Kazuaki Taguchi, Yuki Wada, Masaki Kawano

**Affiliations:** ^1^ Faculty of Pharmacy Keio University 1-5-30 Shibakoen. Minato-ku Tokyo 105-8512 Japan; ^2^ Department of Chemistry School of Science Tokyo Institute of Technology 2-12-1 Ookayama Meguro-ku Tokyo 152-8550 Japan

**Keywords:** protein modification, N-terminus, maleimides, copper, antibody-drug conjugates

## Abstract

Maleimide derivatives are privileged reagents for chemically modifying proteins through the Michael addition reaction with cysteine due to their selectivity, operational simplicity, and commercial availability. However, since accessible free cysteine is rarely found in natural proteins, it is highly desirable to find alternative targets to enable direct bioconjugation of proteins with maleimides. In this study, we have developed an operationally simple and straightforward method for the N‐terminal modification of proteins without the need for mutagenesis via a copper(II)‐mediated [3+2] cycloaddition reaction with maleimides and 2‐pyridinecarboxaldehyde (2‐PCA) derivatives under non‐denaturing conditions at pH 6 and 37 °C in aqueous media. Our method utilizes commercially available maleimides to attach diverse functionalities to various N‐terminal amino acids. We demonstrate the preparation of a ternary protein complex cross‐linked at the N‐termini and dually modified trastuzumab equipped with monomethyl auristatin E (MMAE), a cytotoxic agent, and a Cy5 fluorophore (MMAE‐Cy5‐trastuzumab). MMAE‐Cy5‐trastuzumab retained human epidermal growth factor receptor 2 (HER2) recognition activity and exerted cytotoxicity against HER2‐positive cells. Furthermore, MMAE‐Cy5‐trastuzumab allowed successful visualization of HER2‐positive cancer cells in mouse tumors. This straightforward method will expand the accessibility of protein conjugates with well‐defined structures in a wide range of research fields.

## Graphical abstract

Maleimides can target the N‐termini of proteins instead of cysteine by the use of 2‐pyridinecarboxaldehyde and copper(II) salt under non‐denaturing conditions. The methodology requires no mutagenesis of proteins and enables the high‐yield attachment of various functional groups to proteins with various N‐terminal amino acids through a simple operation using commercially available maleimides.

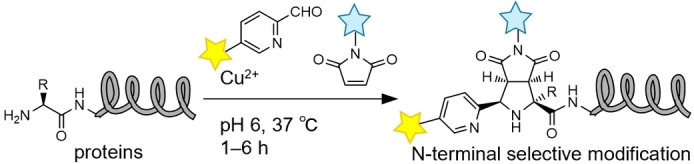

## Introduction

Bioconjugation reactions for protein modification enable the crosslinking of proteins with synthetic molecules and biopolymers, facilitating the labeling and creation of novel functionalized proteins not found in nature.[^1^, ^2^, ^3^] These reactions have been continually developed to meet the increasing demand in various fields, including activity‐based protein profiling,[^4^, ^5^, ^6^, ^7^, ^8^] N‐terminomics,[^9^, ^10^] investigations into cellular processes,[^11^, ^12^] fabrication of protein–biomolecule complexes and biopolymer‐based materials,[^13^, ^14^] and preparation of advanced biopharmaceuticals.[^15^, ^16^, ^17^] Ensuring a conjugate with a uniform structure and properties necessitates precise control over the protein‘s reaction site and the number of attached molecules, even under neutral pH and temperature conditions in aqueous solutions. Additionally, protein modifications must proceed with high yields and reaction kinetics at low concentrations of substrates and reagents. To address this challenge, researchers have explored the chemical modification of rare canonical amino acids such as cysteine,[^18^, ^19^, ^20^, ^21^, ^22^] tyrosine,[^23^, ^24^, ^25^, ^26^, ^27^, ^28^] tryptophan,[^29^, ^30^, ^31^, ^32^, ^33^] methionine,[^34^, ^35^, ^36^] N‐terminus,[^37^, ^38^, ^39^] and C‐terminus,^[40]^ as well as the genetic incorporation of non‐canonical amino acids with bioorthogonal handles,^[41]^ or polypeptide tags[^42^, ^43^, ^44^, ^45^, ^46^] as unique chemically or enzymatically reactive sites. Among these approaches, direct single‐site chemical modification of canonical amino acids is particularly attractive due to its simplicity and convenience. Remarkable progress has been made in the chemical modification of specific amino acids in proteins of interest. However, despite these advancements, achieving high chemo‐ and/or site‐selectivity often requires elaborately synthesized reagents with unique structures and finely tuned reaction conditions. This complexity hinders their widespread application by individuals including non‐chemists in various fields, who demand simplicity, reproducible protocols, and minimal need for specialized equipment. In this context, cysteine modification via Michael addition with maleimide derivatives remains the gold standard due to its high selectivity, operational simplicity, and the wide availability of maleimide derivatives.[^47^, ^48^, ^49^, ^50^] Currently, various maleimide derivatives with specialized functional groups, such as photoactive groups, fluorophores, affinity tags, bioorthogonal handles, spin labels, pharmaceuticals, and hydrophilic solubilizing agents, are commercially available. However, since accessible free cysteine is rarely found in the proteins of interest, the genetic incorporation of a reactive cysteine often becomes necessary after all. In contrast, N‐termini are present in all proteins and are frequently located on their surface as unique reactive sites. Therefore, developing simple and reliable alternative methods using readily accessible maleimide derivatives to modify N‐terminal amino acids in proteins without requiring mutagenesis could have a notable impact across various scientific fields.^[51]^

**Figure 1:**
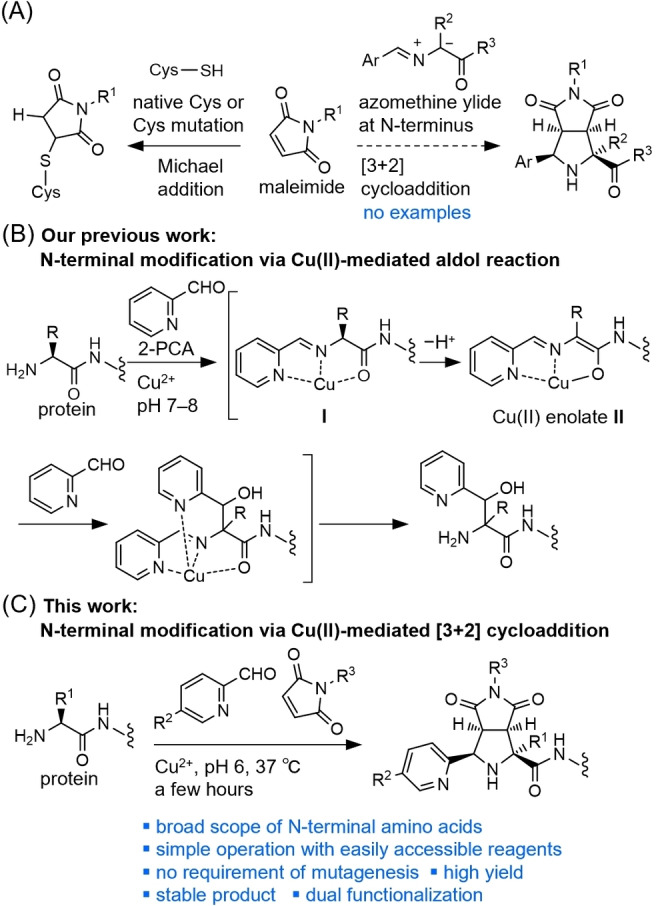
A) Modification of canonical amino acids in proteins with maleimide derivatives. B) Selective N‐terminal modification of proteins via aldol reaction using in situ generated copper(II) enolate. C) Selective N‐terminal modification of proteins via copper(II)‐mediated [3+2] cycloaddition under non‐denaturing conditions.

Maleimides are known as excellent Michael acceptors as well as dienophiles and 1,3‐dipolarophiles that undergo [4+2] or [3+2] cycloadditions (Figure 1A). Azomethine ylides, which were developed by Grigg and Kemp,[^52^, ^53^, ^54^] are bio‐relevant 1,3‐dipoles that can be generated from imines of α‐amino acids and aryl aldehydes. If azomethine ylides could be easily constructed on various N‐terminal amino acids of proteins using a straightforward method, such as by mixing all required reagents under non‐denaturing conditions at ambient temperature and pH in an aqueous solution, the [3+2] cycloaddition of azomethine ylides with maleimides would offer a convenient and practical approach for the selective N‐terminal modification of proteins. However, despite the long history of [3+2] cycloaddition of azomethine ylides,[^55^, ^56^, ^57^, ^58^] no examples of protein modification exist, likely due to several challenges. These challenges include the low stability of amino acid imines in aqueous conditions and the need for bioincompatible basic conditions to generate azomethine ylides in situ via the deprotonation of the amino acid imine. Furthermore, substituted amino acids are less reactive precursors of azomethine ylides due to the lower acidity of the α‐proton caused by the inductive effect and the inefficiency of imine formation due to steric hindrance. As a result, the substrate scope is limited, primarily restricted to glycine esters and short peptides with N‐terminal glycine, when using conventional conditions with metal salts and phosphine‐based ligands under basic conditions in organic solvents. Therefore, the N‐terminal modification of proteins with different N‐terminal amino acids through [3+2] cycloaddition of azomethine ylide calls for a completely new approach to improve both imine formation and α‐proton deprotonation.

We have recently reported the selective N‐terminal modification of peptides and proteins with various N‐terminal amino acids via copper(II)‐mediated aldol reaction with pyridine‐2‐carboxaldehyde (2‐PCA) derivatives at a neutral pH and 37 °C (Figure 1B).^[59]^ The copper(II) ion forms a stable ternary complex **I** with the Schiff base of the N‐terminal amino acid and 2‐PCA, even in aqueous media. Due to the Lewis acidity of the metal ion, deprotonation of the α‐proton occurs at neutral pH, generating a copper(II) enolate **II** in situ. Intermediate **II** reacts with 2‐PCA to afford the aldol product after the decomposition of the Schiff base–copper(II) complex. Lysine also forms a Schiff base–metal complex on its side chain, which is inert during this process due to its low acidity. Lysine is regenerated after quenching, resulting in selective N‐terminal modification.

We expect that the in situ*‐*generated intermediate **II** will serve as a potential 1,3‐dipole species. Herein, we have devised a user‐friendly and practical approach to selectively modify the N‐termini of proteins through a copper(II)‐mediated [3+2] cycloaddition reaction with 2‐PCA derivatives and commercially available maleimide derivatives (Figure 1C). The developed method efficiently modifies a wide range of peptides and proteins with diverse N‐terminal amino acids. The process involves simply mixing all the necessary reagents for a few hours at pH 6 and 37 °C, without the requirement for prior introduction of special sequence tags or mutations. Additionally, by employing 2‐PCA with azide tags, we have successfully generated modified proteins that incorporate two different synthetic molecules.

## Results and Discussion

**Figure 2:**
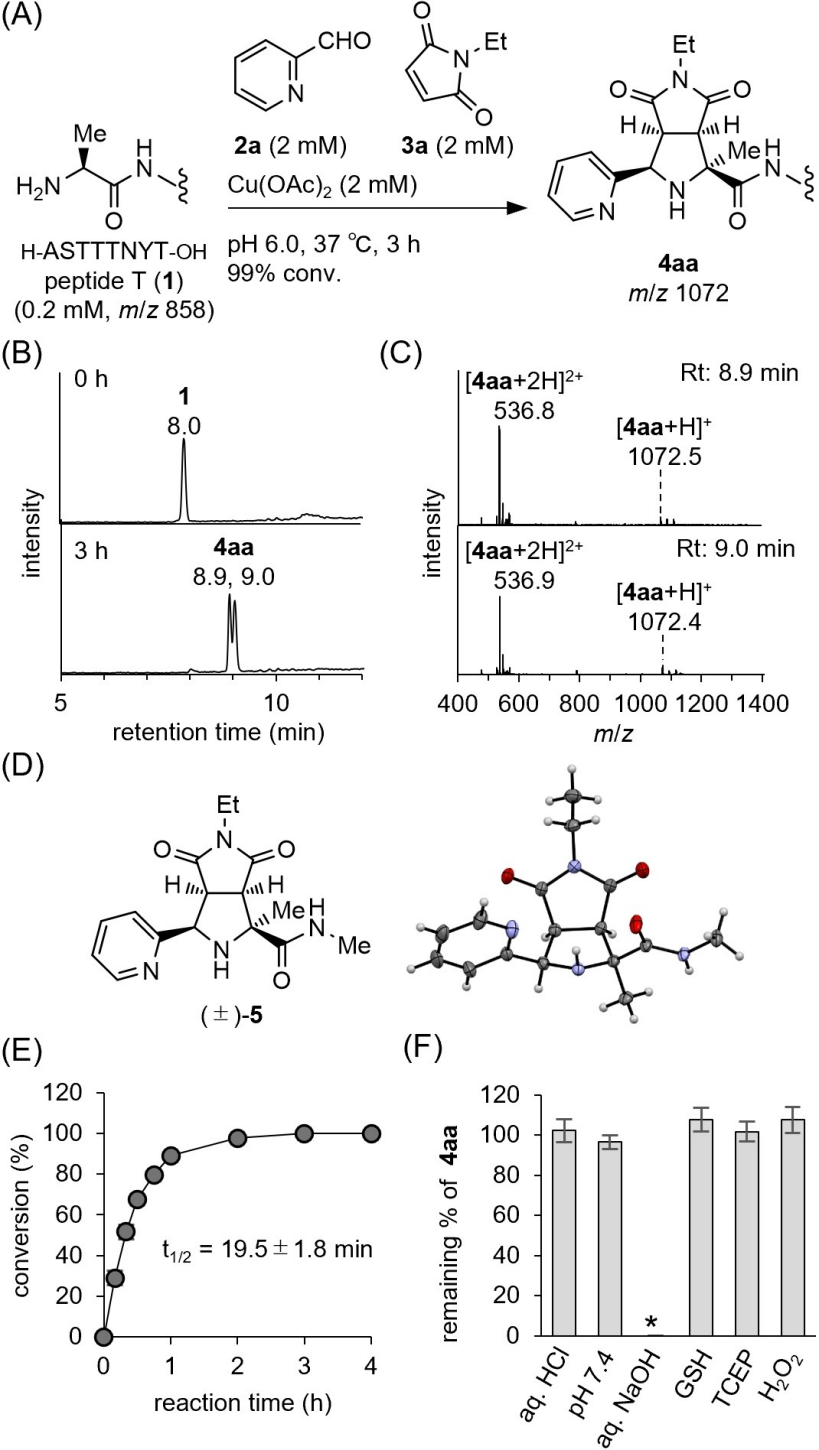
A) N‐terminal modification of peptide T (**1**) via copper(II)‐mediated [3+2] cycloaddition with pyridine‐2‐carboxaldehyde (**2 a**) and N‐ethylmaleimide (**3 a**). B) Total ion chromatograms from LC–MS analyses of the crude reaction mixture of **1** with **2 a** and **3 a**. C) Mass spectra of the fractions at 8.9 minutes and 9.0 minutes in LC–MS analysis. D) ORTEP diagram of (±)**‐5**, representing the N‐terminal substructure of **4 aa** (probability level: 50 %). E) Time course of the N‐terminal modification of peptide T (**1**) with **2 a** and **3 a**. F) Stability test of **4 aa**. Each bar represents the average remaining percentage of **4 aa** after incubation at 37 °C for 3 days (*n*=3). Error bars represent SD. *After incubation for 5 min.

The biologically active peptide T (**1**) with an N‐terminal alanine was used to explore the reaction conditions of [3+2] cycloaddition with *N*‐ethylmaleimide (**3 a**) applicable for protein modification. The conventional combination of 4‐methylbenzaldehyde, copper(I) salt, and hydrophobic phosphine‐based ligands such as XantPhos,^[58]^ proved unsuitable for reactions in aqueous media due to the extremely low aqueous solubility of the ligands. Further investigation revealed that the reaction of **1** with 2‐PCA (**2 a**) and *N*‐ethylmaleimide (**3 a**) in the presence of Cu(OAc)_2_ at 37 °C in a buffer solution at pH 6 provided the desired adduct **4 aa** quantitatively within 3 h (Figures 2A and 2B). Two new peaks at retention times of 8.9 and 9.0 minutes with the same *m*/*z* of 1072 (ΔM=+214 amu) were observed in LC–MS, indicating that both peaks corresponded to the two diastereomers of **4 aa** (Figure 2C). The modification site in **4 aa** was identified at the N‐terminus by LC–MS/MS analysis (Figure S1). Further structural elucidation of adduct **4 aa** was conducted using NMR (Figure S2–S4 and Table S1). In the ^1^H NMR spectra, a proton at the α‐position of the N‐terminal alanine (δ 3.88–3.85 ppm) in **1** disappeared after modification. The methyl signal of alanine changed from a doublet (δ 1.32 ppm) in **1** to a singlet (δ 1.50 or 1.59 ppm) in **4 aa**. HSQC and HMBC results indicated the presence of a quaternary carbon (67.3 ppm in ^13^C NMR of **4 aa**) at the N‐terminal residue, suggesting a 1,3‐dipolar cycloaddition at the N‐terminus, as expected. To estimate the stereochemistry of **4 aa**, the alanine derivative (±)‐**5**, the N‐terminal substructure of **4 aa**, was synthesized by copper(II)‐mediated [3+2] cycloaddition of alanine amide with **2 a** and **3 a**. Single crystal X‐ray analysis of (±)‐**5** revealed the endo‐configuration, suggesting that **4 aa** was a diastereomeric mixture of the endo products (Figure 2D).^[60]^ The N‐terminal modification of **1** with **2 a** and **3 a** proceeded at pH 6.0–8.0 with negligible amounts of byproducts such as aza‐Michael addition adducts at the N‐terminus with **3 a** (ΔM=+125 amu) and aldol adducts with **2 a** (ΔM=+107 amu and +196 amu) (Figure S5). To minimize the formation of these potential byproducts in further studies involving larger peptides and proteins, we concluded that a weakly acidic pH of 6.0 is optimal. Kinetic studies revealed that the half‐life of **1** at pH 6.0 was estimated to be 19.5±1.8 min (Figures 2E and S6 A). During the kinetic studies, we observed aldol product formation when the relative concentration of **2 a** to **3 a** was increased, indicating that an excess amount of **3 a** was key to obtaining **4 aa** in a high yield (Figure S6B). The newly constructed substructure in adduct **4 aa** remained stable at acidic and neutral pH or in the presence of TCEP, glutathione, and hydrogen peroxide at 37 °C (Figure 2F). Succinimide ring‐opening hydrolysis was negligible after incubation for three days under these conditions (Figure S7). In contrast, succinimide ring‐opening hydrolysis was completed at a basic pH within 5 minutes, resulting in a complex mixture after incubation for 1 day (Figure S8).

Other metal ions were examined as potential promoters of the N‐terminal modification of **1** via [3+2] cycloaddition with **2 a** and **3 a** (Figure S9). In the presence of nickel(II) and cobalt(II) salts, **1** exhibited 96 % and 88 % conversion, respectively, forming various products through aldol reaction (ΔM=+107 amu), imidazolidinone formation (ΔM=+89 amu),^[37]^ aza‐Michael addition (ΔM=+125 amu), and transamination (ΔM=+28 amu). Interestingly, nickel(II) salt accelerated the aza‐Michael addition of **1** and **3 a**. Although silver(I)[^61^, ^62^, ^63^, ^64^] and zinc(II)[^65^, ^66^] salts are generally used in the [3+2] cycloaddition of azomethine ylides derived from substituted amino acids in organic solvents, the conversion of **1** was markedly low (<10 %). The Lewis acidity of these ions was insufficient to enhance both imine formation and deprotonation of the α‐proton. Ultimately, we concluded that copper(II) salt provided the highest conversion of **1** (>95 %), with **4 aa** exclusively formed as the major product.

Subsequently, we assessed the conversion of **1** in reactions with other aryl aldehydes **2 b**–**k** using LC–MS (Table 1, Figures S10 and S11). The modification reaction demonstrated strict requirements for aryl aldehydes with adjacent coordination groups. Benzaldehyde (**2 b**) and salicylaldehyde (**2 c**) were unreactive, whereas heterocyclic aldehydes **2 d**–**f** achieved high conversion and provided the corresponding adducts without noticeable byproducts. Pyridone‐3‐carboxaldehyde (**2 f**) likely transformed in situ into the corresponding hydroxypyridine, which participated in the reaction. 8‐Quinolinecarboxaldehyde (**2 g**), capable of forming a six‐membered chelation ring, produced the adduct, albeit with lower reactivity. 3‐Pyridinecarboxaldehyde (**2 h**), possessing a formyl group at the 3‐position, resulted in negligible conversion, suggesting that the formation of a five‐membered chelation ring at the N‐terminus plays an important role in the [3+2] cycloaddition. Methyl‐substituted 2‐PCAs **2 i**–**k** were examined to determine the most favorable positions for introducing functionalities. Gratifyingly, the substituent position on the 2‐PCA ring had little impact on reactivity.

**Table 1:**
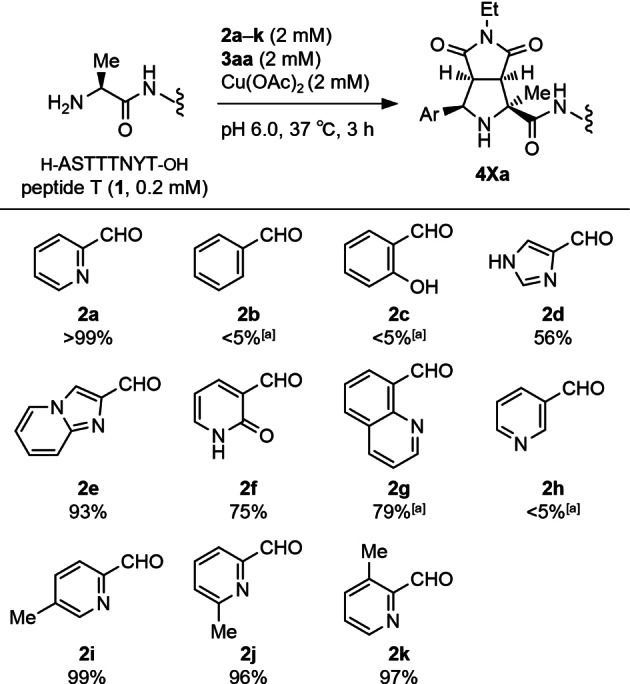
Scope of aryl aldehyde for N‐terminal modification of peptide T (**1**)

[a] Conversion of peptide T (**1**) after the reaction for 18 h.

1,3‐Dipolarophiles **3 a**–**e**, commonly employed in bioconjugations, were evaluated for their suitability in the N‐terminal modification of **1**. Among the compounds tested, *N*‐ethylmaleimide (**3 a**) and *N*‐phenylmaleimide (**3 b**) exhibited sufficient reactivity for practical applications (Table S2 and Figure S12). However, **3 b** yielded ring‐opened adducts **4 ab′**, which were formed through hydrolysis of the imide ring in **4 ab**.^[50]^ For phenyl vinyl sulfone (**3 c**) and ethyl acrylate (**3 d**), which have limited reactivity, **1** is consumed in the copper(II)‐mediated aldol reaction and subsequent imidazolidinone formation, resulting in a mixture of singly and doubly modified products of **2 a**, as previously reported. The use of bicyclononyne **3 e** produced a complex mixture of unidentified products. Considering the reactivity and commercial availability of the functionalized derivatives, we conclude that *N*‐alkylmaleimides are the most potent reagents for N‐terminal modification via copper(II)‐mediated [3+2] cycloaddition.

**Figure 3:**
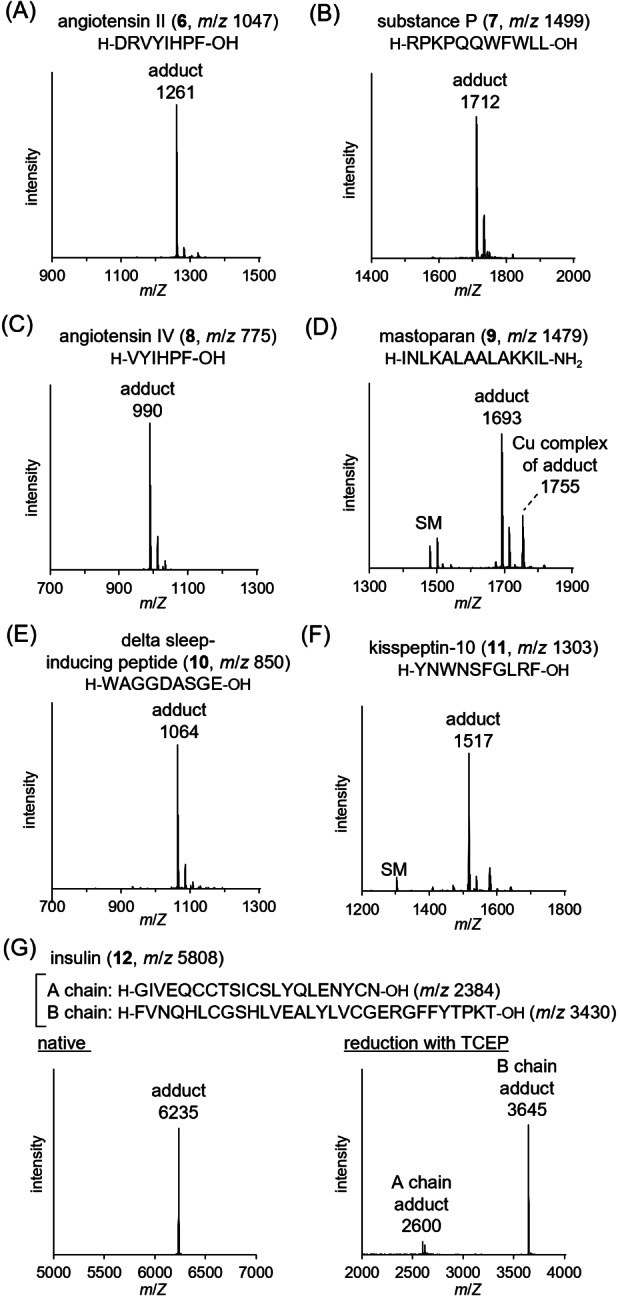
MALDI‐TOF MS spectra of crude reaction mixtures of N‐terminal modification of biologically active peptides with various N‐terminal amino acids with **2 a** and **3 a**. A) Angiotensin II (**6**). B) Substance P (**7**). C) Angiotensin IV (**8**). D) Mastoparan (**9**). E) Delta sleep‐inducing peptide (**10**). F) Kisspeptin‐10 (**11**). G) Insulin (**12**). Conditions: peptide (0.2 mM), **2 a** (2 mM), **3 a** (2 mM), Cu(OAc)_2_ (2 mM) in phosphate buffer (10 mM, pH 6.0) at 37 °C for 6 h, then EDTA (4 mM) and methoxyamine (40 mM).

The applicability of the developed method to various N‐terminal amino acids was evaluated using biologically active peptides **6**–**12** (Figures 3 and S13). The reactions provided the corresponding adducts without any noticeable byproducts, as confirmed by MALDI‐TOF MS and LC–MS analyses. Notably, even after the addition of EDTA, the copper(II) complex of the adduct of **9** was observed suggesting that the side chain of the second residue, asparagine, complexes with the copper(II) ion, in addition to the nitrogen atoms of pyridine, pyrrolidine, and backbone amide, forming a stable complex (Figure 3D). For insulin (**12**), both the N‐termini of the A and B chains were modified, resulting in doubly modified products (Figure 3G). The N‐terminal modifications of peptides **6**–**11** were confirmed by LC–MS/MS (Figures S14–S19). N‐terminal amino acids with charged, bulky, and aromatic side chains were compatible with the reaction. Even with multiple lysine residues capable of undergoing the aza‐Michael reaction, specific modifications were observed at the N‐terminus (Figure 3D). In addition to the weakly acidic pH, which decreases the nucleophilicity of the lysine amino group of Lys, the amino group of lysine is transiently protected as a Schiff base–metal complex during the reaction, minimizing aza‐Michael addition. Peptides with N‐terminal metal‐binding domains (His or Gly‐Asn‐His) showed significantly low reactivity. Pyroglutamic acid and N‐acetylated amino acids at the N‐terminus afforded no detectable adducts (Figures S20 and S21). These results strongly suggest the requirement for the formation of a ternary copper(II) complex.

**Figure 4:**
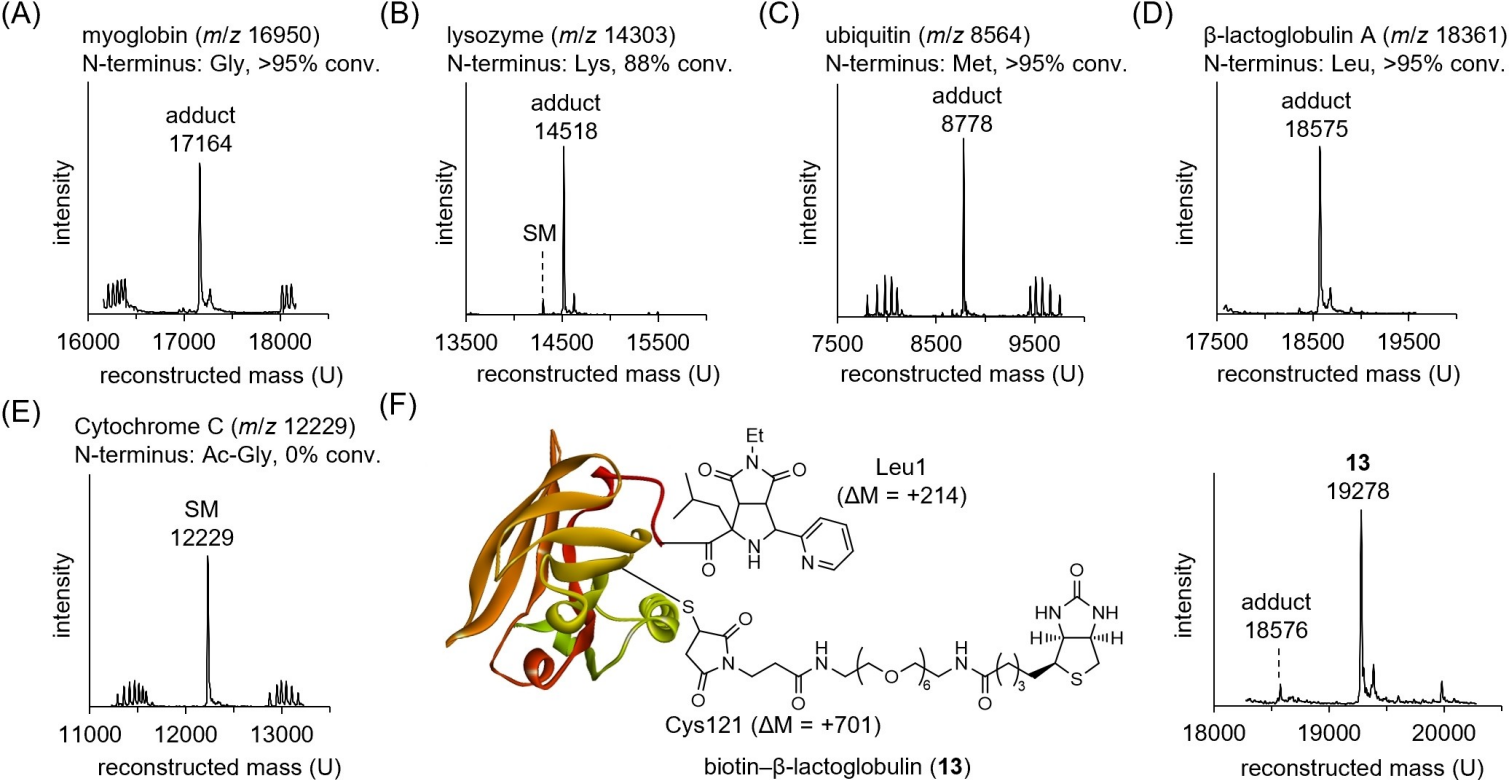
Deconvoluted mass spectra of crude reaction mixture of N‐terminal modification of proteins with **2 a** and **3 a**. A) Myoglobin. B) Lysozyme. C) Ubiquitin. D) β‐Lactoglobulin A. E) Cytochrome C. Conditions: protein (0.8 mg/mL), **2 a** (2 mM), **3 a** (2 mM), Cu(OAc)_2_ (2 mM) in phosphate buffer (10 mM, pH 6.0) at 37 °C for 1 h for ubiquitin and β‐lactoglobulin, 3 h for myoglobin, or 6 h for lysozyme, then EDTA (4 mM) and methoxyamine (40 mM). F) β‐Lactoglobulin A after sequential modification at N‐terminal Leu (Leu1) and Cys121.

The N‐terminal modification developed can be applied to proteins. We performed modification reactions on myoglobin (N‐terminus: Gly), lysozyme (N‐terminus: Lys), ubiquitin (N‐terminus: Met), β‐lactoglobulin A (N‐terminus: Leu), and cytochrome C (N‐ terminus: Ac‐Gly) with **2 a** and **3 a** (Figures 4A−E and S22–S26).

In all reactions, except for cytochrome C, high conversion of the parent protein was observed, and the expected conjugate (ΔM=+214) was detected as the major product (Figures 4A–D). Tryptic digestion of each modified protein revealed that the modification occurred exclusively on the N‐terminus‐containing fragments (Figures S27–S34). Cytochrome C, with an acetylated N‐terminus, was unreactive, consistent with the peptide study (Figure 4E). Notably, only a trace amount of the **3 a** adduct resulting from the aza‐Michael addition of lysine, was detected.

To assess the inhibitory effect of acidic pH and each reagent on the aza‐Michael addition in protein modifications, reactions of cytochrome C and **3 a** were conducted in the presence or absence of **2 a** and copper(II) salt at pH 6.0 or 7.5 (Figure S35). The results revealed that both acidic pH and copper(II) ions contributed to the suppression of the aza‐Michael addition. In particular, when both **2 a** and copper(II) ions were used, the formation of the adduct with **3 a** was most suppressed, even at pH 7.5, suggesting that lysine was transiently protected by forming a Schiff base–copper(II) complex during the reaction (Figures S35E and S35G).

Notably, β‐lactoglobulin A was modified with **2 a** and **3 a** at the N‐terminus despite the presence of one free cysteine (Cys121). Both the acidic pH and copper(II) ions inhibited cysteine modification with **3 a** during the [3+2] cycloaddition (Figure S36). This allowed us to sequentially modify the N‐terminal and Cys residues. The residual Cys121 was modified by 1,4‐addition with biotin–PEG6–maleimide **14** at pH 7.5, forming the dually modified β‐lactoglobulin A **13** (Figures 4F and S37).

**Figure 5:**
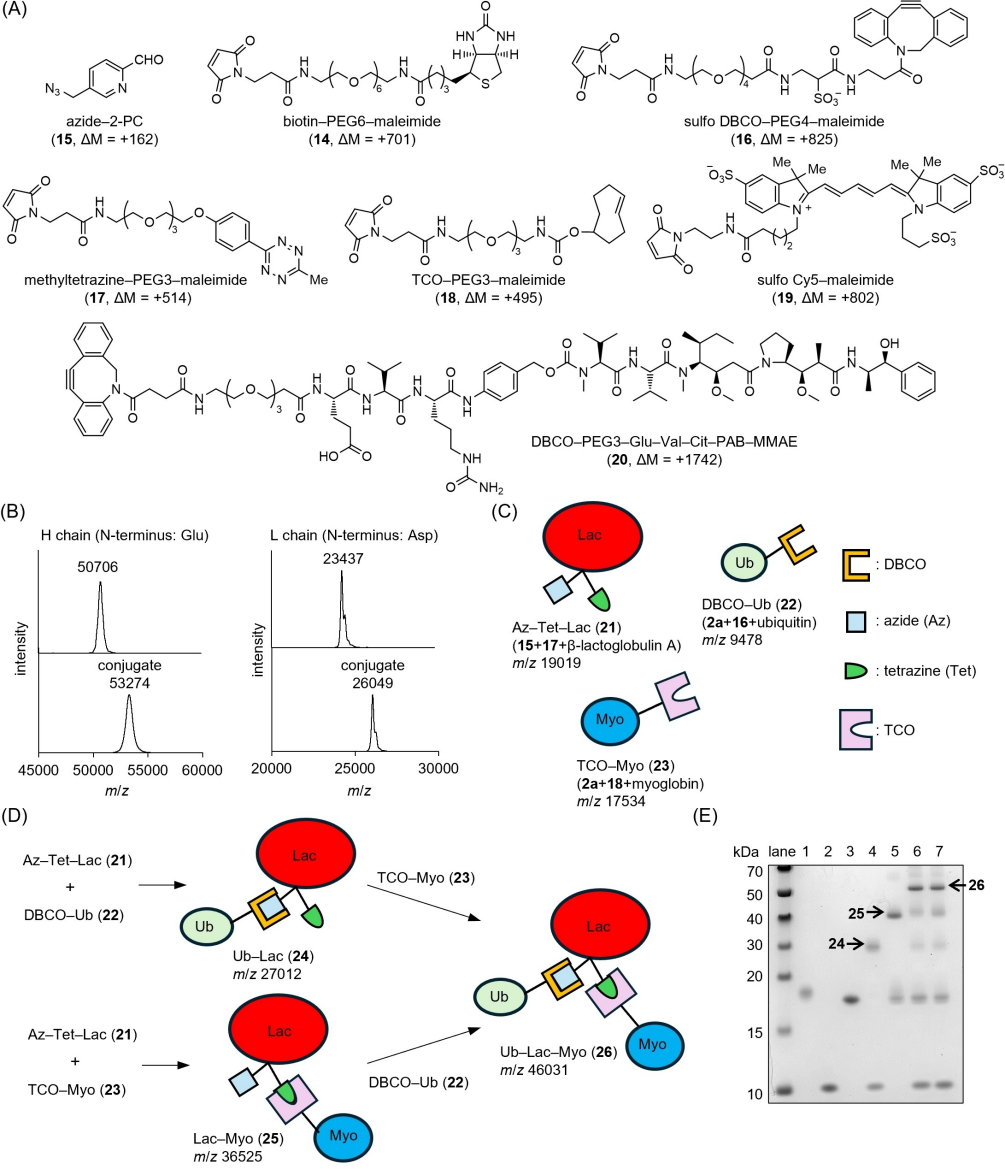
A) Structures of reagents used in the experiments. B) MALDI TOF‐MS spectra of the heavy (H) chains and light (L) chains of trastuzumab (upper) and MMAE–biotin–trastuzumab (lower). C) Schematic presentation of modified proteins with click chemistry handles. D) Scheme for the sequential preparation of protein clusters **24**, **25**, and **26** by click chemistry. E) Gel image of modified proteins and protein clusters. Lane 1: Az–tetrazine–Lac **21**; lane 2: DBCO–Ubi **22**; lane 3: TCO–Myo **23**; lane 4: reaction mixture of **21** and **22**; lane 5: reaction mixture of **21** and **23**; lane 6: reaction mixture of **21**, **22**, then **23**; lane 7: reaction mixture of **21**, **23**, and then **22**.

Functionalized 2‐PCA and maleimides were utilized for N‐terminal modification (Figure 5A). Trastuzumab, an anti‐human epidermal growth factor receptor 2 (HER2) antibody used to treat metastatic breast cancer, reacted with biotinylated maleimide **14** and azide‐2‐PCA **15** in the presence of copper(II) ions. After removing unreacted **14** and **15** using a desalting column, monomethyl auristatin E (MMAE), a microtubule inhibitor, was incorporated through click chemistry between azide and dibenzocyclooctyne (DBCO) using DBCO–PEG3–Glu–Val–Cit–PAB–MMAE (**20**). Despite the high molecular weight (~150 kDa) and complex structure of trastuzumab, MALDI‐TOF MS analyses revealed that both the heavy chain (N‐terminus: Glu) and light chain (N‐terminus: Asp) were completely consumed and modified with one set of **14**, **15**, and **20** (theoretical ΔM=+2571) (Figure 5B).

Introducing click chemistry handles to the N‐termini via 1,3‐dipolar cycloaddition with commercially available maleimides **16–18** enabled the construction of cross‐linked proteins with defined structures (Figures 5C and 5D). Azide–tetrazine–β‐lactoglobulin A (Az–Tet–Lac, **21**), DBCO–ubiquitin (DBCO−Ub, **22**), and trans‐cyclooctene–myoglobin (TCO–Myo, **23**) were prepared using the developed method (Figures S38 and S39). The resulting conjugates were coupled to each other at their N‐termini via strain‐promoted azide‐alkyne cycloaddition between azide and DBCO, and the inverse electron demand Diels–Alder reaction between tetrazine and cyclooctene. Formation of the binary conjugates Ub–Lac (**24**) and Lac–Myo (**25**) was confirmed by SDS‐PAGE and size‐exclusion column analyses. Furthermore, the ternary conjugate Ub–Lac–Myo (**26**) was successfully obtained by subsequent reactions of **24** with **23**, and **25** with **22** (Figures 5E and S40).

**Figure 6:**
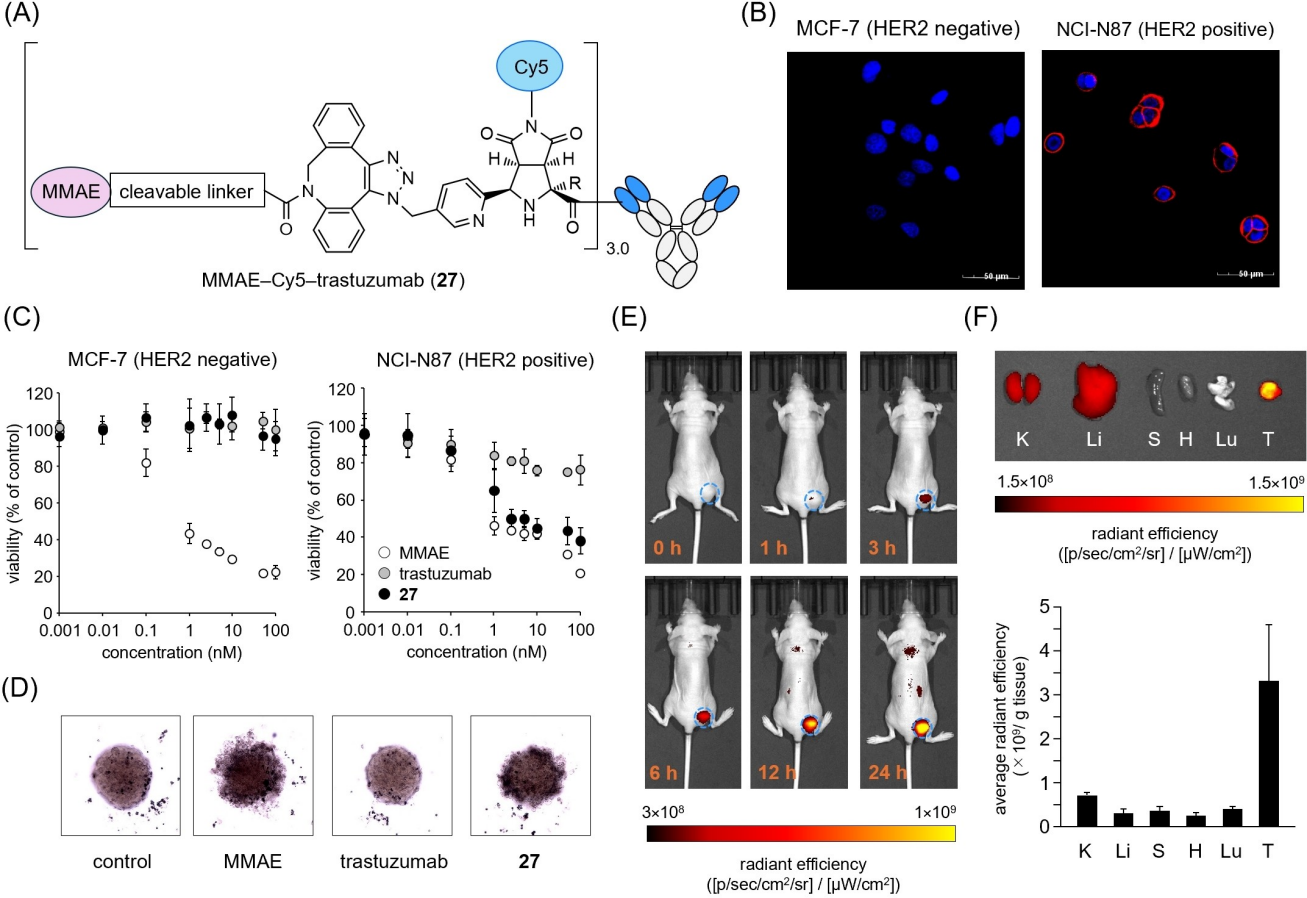
A) Schematic view of MMAE–Cy5–trastuzumab **27**. B) Representative confocal microscopy images of human epidermal growth factor receptor 2 (HER2‐positive cells NCI‐N87 cells and HER2‐negative cells MCF‐7 cells treated with **27** (red). Blue: nuclear stain with DAPI. Scale bar: 50 μm. C) Cytotoxicity assay of MMAE, trastuzumab, and **27** on HER2‐positive NCI‐N87 cells and HER2‐negative MCF‐7 cells. Values on each bar represent the average cell viability (*n*=3). Error bars represent SD. D) Representative morphology of NCI‐N87 spheroids treated with MMAE (10 nM), trastuzumab (3.5 nM), and **27** (MMAE: 10 nM, trastuzumab: 3.5 nM). E) Representative fluorescence images of the NCI‐N87‐bearing mice at 0, 1, 3, 6, 12, and 24 h after the administration of **27**. The tumor is circled with a light blue dashed line. F) Ex vivo fluorescence images and intensities of tumors and major organs at 24 h after the administration of **27**. K, Li, S, H, Lu, and T indicate kidneys, liver, spleen, heart, lungs, and tumor, respectively. Values on each bar represent the average radiant efficiency (*n*=4). Error bars represent SD.

Next, we evaluated the compatibility of the N‐terminal modification via copper(II)‐mediated [3+2] cycloaddition with the intrinsic activity of the proteins. We prepared MMAE‐Cy5–trastuzumab (**27**) via the copper(II)‐mediated [3+2] cycloaddition of **15** and **19**, followed by a copper‐free click reaction with DBCO–PEG3–Glu–Val–Cit–PAB–MMAE (**20**) (Figures 6A and S41).

MMAE‐Cy5–trastuzumab (**27**) was expected to not only visualize HER2‐positive tumor cells but also release the active payload MMAE specifically at the tumor site upon cathepsin‐mediated cleavage of the Glu–Val–Cit linker,^[67]^ thereby treating cancer. The developed method provided **27** with an average of 3.0 molecules of MMAE and Cy5 molecules attached to the Fab regions of both the heavy and light chains, as demonstrated by UV/Vis. spectroscopy, MALDI‐TOF MS, and in‐gel fluorescence detection following papain treatment (Figure S42). The residual copper in 1.5 μM of **27** was below the detection limit (0.2 μg/L) of the ICP‐OES analysis. Surface plasmon resonance (SPR) analysis revealed that the binding affinity of **27** to HER2 (*K*_D_=1.2 nM) was comparable to that of trastuzumab (*K*_D_=1.1 nM) (Figure S43). We assessed the binding of **27** to HER2 on the cell surface by confocal microscopy (Figures 6B and S44). Although there was no significant fluorescence signal from HER2‐negative human breast adenocarcinoma cells MCF‐7, strong Cy5‐derived fluorescence signals were observed on the surface of HER2‐positive human gastric cancer cells NCI‐N87. These results suggest that the N‐terminal modification of trastuzumab had little impact on the structure of the antigen recognition site on the N‐terminal domain. The anti‐proliferative activity of **27** on MCF‐7 and NCI‐N87 cells was compared with that of the parent drugs, trastuzumab and MMAE, in 2D‐ and 3D‐cultured models (Figures 6C and 6D). While MMAE showed a non‐specific inhibitory effect on both tumor cells in the 2D‐cultured model, trastuzumab and **27** exhibited specific anti‐proliferative activity only in HER2‐positive NCI‐N87 cells (Figure 6C). Conjugate **27** displayed more potent activity than the parent drug, trastuzumab, at concentrations above 1 nM, comparable to that of MMAE. These results suggest that **27** did not release MMAE outside the cells but was internalized into the cells via HER2 receptor‐mediated endocytosis to release MMAE into the cytosol through cathepsin‐mediated hydrolysis of the Glu–Val–Cit linker. In the 3D culture model, trastuzumab did not affect the spheroid shape, whereas MMAE and conjugate **27** inhibited the growth of NCI‐N87 spheroids and caused the spheroids to collapse (Figures 6D and S45). As the tumor spheroid mimics the in vivo tumor microenvironment,^[68]^ the data from the 3D‐cultured model strongly corroborate the potent anti‐tumor activity of conjugate **27**. Finally, we assessed the biodistribution of **27** in NCI−N87‐bearing mice by IVIS in vivo fluorescence imaging. MMAE‐Cy5–trastuzumab (**27**) (0.22 mg/kg) was intravenously administered to the NCI‐N87‐bearing mice, and the fluorescence images were captured at the specified time points (Figure 6E). The fluorescence signal of Cy5 was observed in the tumor at 3 h after administration and continued to increase until 24 h (Figure 6E). Quantification of the fluorescence signals in the dissected organs indicated a high accumulation of **27** in the tumor tissue (Figure 6F).

## Conclusion

In summary, this study demonstrated the potential of N‐terminal amino acids as alternative modification targets for maleimide derivatives. We applied maleimide derivatives for selective N‐terminal modification of peptides and proteins with various N‐terminal amino acids via a copper(II)‐mediated [3+2] cycloaddition using commercially available 2‐PCA (**2 a**). The method requires no mutagenesis or special sequence at the N‐terminus, representing a significant advancement over other chemical modification of canonical amino acids, including cysteine mutagenesis. Although the reaction rate of a copper(II)‐mediated [3+2] cycloaddition (*t*_1/2_=19.5 minutes) is slower than cysteine modification via Michael addition at pH 7.4 (*t*_1/2_≤1 minute), the experimental procedure is straightforward. It involves incubating all reagents under weakly acidic conditions at pH 6.0 and 37 °C for 1–6 h. Additionally, the combination with readily accessible azide–2‐PCA **15** enabled dual functionalization of the N‐terminus with chemical probes or cytotoxic agents in a stepwise manner. This developed methodology allowed the construction of modified proteins using click chemistry handles, and the formation of binary and ternary complexes were cross‐linked at their N‐termini. We successfully applied N‐terminal modification to prepare MMAE‐Cy5–trastuzumab (**27**), a dually modified antibody–drug conjugate that displayed HER2 recognition activity in vitro and in vivo and exhibited anti‐proliferative activity in 2D‐ and 3D‐cultured HER2‐positive cells.

For selective N‐terminal modification via copper(II)‐mediated [3+2] cycloaddition in weakly acidic to neutral aqueous solutions, heteroaromatic aldehydes with the coordination motifs, such as 2‐PCA (**2 a**), were necessary. In conventional metal‐mediated [3+2] cycloadditions of amino acid imines with maleimides in organic solvents, the bidentate coordination of amino acid imines with metal ions promotes the formation of azomethine ylides. However, because amino acid imines are vulnerable to hydrolysis, additional coordination from heteroaromatic nitrogen is required to stabilize the imines in aqueous solution. Furthermore, the electron‐withdrawing inductive effect of the heteroaromatic ring increases the acidity of the α‐proton, presumably allowing the formation of azomethine ylide formation under biocompatible conditions without the addition of strong bases. Heteroaromatic aldehydes also advantageously protect the amino groups of lysine residues transiently during the reaction through copper(II)‐imine complex formation, suppressing undesired aza‐Michael addition and resulting in high N‐terminal selectivity.

There have been very few examples of single‐step modifications that exclusively target the N‐termini of proteins without requiring specific residues or sequences at N‐termini.[^37^, ^39^, ^59^] Among these, 2‐PCA derivatives themselves offer an exceptionally simple approach for selective N‐terminal modification through imidazolidinone formation without the addition of any metal salts.^[37]^ However, since imidazolidinone formation with 2‐PCAs is not a fast process, it often requires long incubation times (typically 16 hours) and a large excess of 2‐PCA (5–10 mM, 200–400 eq) to ensure sufficient protein conversion. Additionally, the adduct tends to partially revert back to the parent proteins and 2‐PCA under typical physiological conditions, resulting in more than 50 % loss after 48 h at pH 7.5 and 37 °C.^[69]^ In our methodology, the addition of copper(II) salt and maleimide derivatives not only reduces reaction time (1–6 hr), decreases the amount of reagents required (2 mM, 10–50 eq), and improves product stability, but also enables multiple functionalization.

An intrinsic drawback of targeting the N‐terminus is that a large proportion of eukaryotic proteins are post‐translationally modified.^[70]^ Furthermore, the copper(II)‐mediated [3+2] cycloaddition may face interference from biologically related molecules that bind copper(II) ions, including glutathione and metal‐binding proteins. These limitations restrict the practical implementation of the method for protein modification in live cells or in vivo studies. However, it is applicable to chemical modification of most peptides and expressed proteins in vitro, excluding peptides and proteins with metal‐binding motifs at the N‐terminus. The widespread availability of maleimide derivatives and the simplicity of the procedure enable researchers to apply this methodology across various fields.

## Conflict of Interests

The authors declare no conflict of interest.

1

## Supporting information

As a service to our authors and readers, this journal provides supporting information supplied by the authors. Such materials are peer reviewed and may be re‐organized for online delivery, but are not copy‐edited or typeset. Technical support issues arising from supporting information (other than missing files) should be addressed to the authors.

Supporting Information

## Data Availability

The data that support the findings of this study are available in the supplementary material of this article.
