# Supplementary material for: One‐Step Maleimide‐Based Dual Functionalization of Protein N‐Termini
Source: Angew Chem Int Ed Engl. 2024 Nov 27;64(5):e202417134. doi: 10.1002/anie.202417134 (PMC11773299; doi:10.1002/anie.202417134)
Supplement: Supplementary file 1 — Supporting Information [file ANIE-64-e202417134-s001.pdf]

Angewandte Chemie
Eine Zeitschrift der Gesellschaft Deutscher Chemiker

Supporting Information

One-Step Maleimide-Based Dual Functionalization of Protein N-Termini

K. Hanaya, K. Taguchi, Y. Wada, M. Kawano*

Supporting Information

One-Step Maleimide-Based Dual Functionalization of Protein N-Termini

Kengo Hanaya,* Kazuaki Taguchi, Yuki Wada, and Masaki Kawano

Table of Contents

1. Supporting Data 2
   The structure determination of the modified peptide T 4aa 2
   Screening of reaction conditions for N-terminal modification of peptide T (1) via the copper(II)-mediated [3+2] cycloaddition 9
   Stability of the modified peptide T 4aa under various biologically relevant conditions 11
   LC-MS analyses of N-terminal modification of proteins with 2a and 3a 28
   Identification of modification site by MALDI-TOF MS analyses of enzyme-digested peptide fragments 33
   Inhibitory effect of copper(II) ion, 2a, and acidic pH on aza-Michael addition 41
   Suppression of Michael addition of cysteine by copper(II) ion, 2a, and acidic pH 42
   Additional experimental data for sequential modification of lactoglobulin at N-terminus and Cys121 43
   Additional experimental data for cross-linked proteins 44
   Characterization of MMAE–Cy5–trastuzumab 27 47
   Additional experimental data for the assessment of anti-proliferative activity of 27 50
2. Experimental procedures........ 52
   2-1. General information........ 52
   2-2. Peptide modification........ 53
   2-3. Protein modification 54
   2-4. Organic syntheses 58
3. ^{1}H and ^{13}C NMR spectra of the key and new compounds 60
4. References........ 68

1. Supporting Data

The structure determination of the modified peptide T 4aa

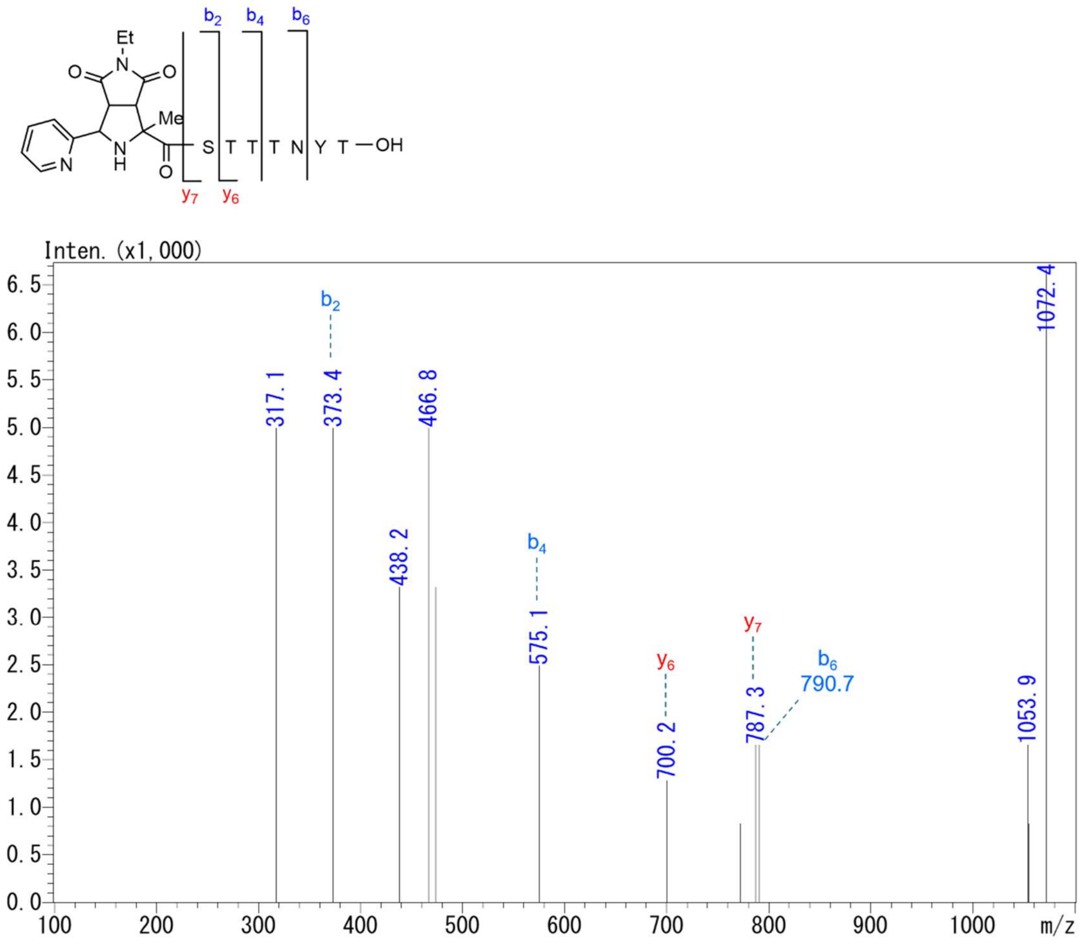

Figure S1. Collision-induced dissociation (CID) and the LC-MS/MS spectrum of the singly charged positive ion at *m*/*z* = 1072.50 corresponding to the modified peptide T (**4aa**).

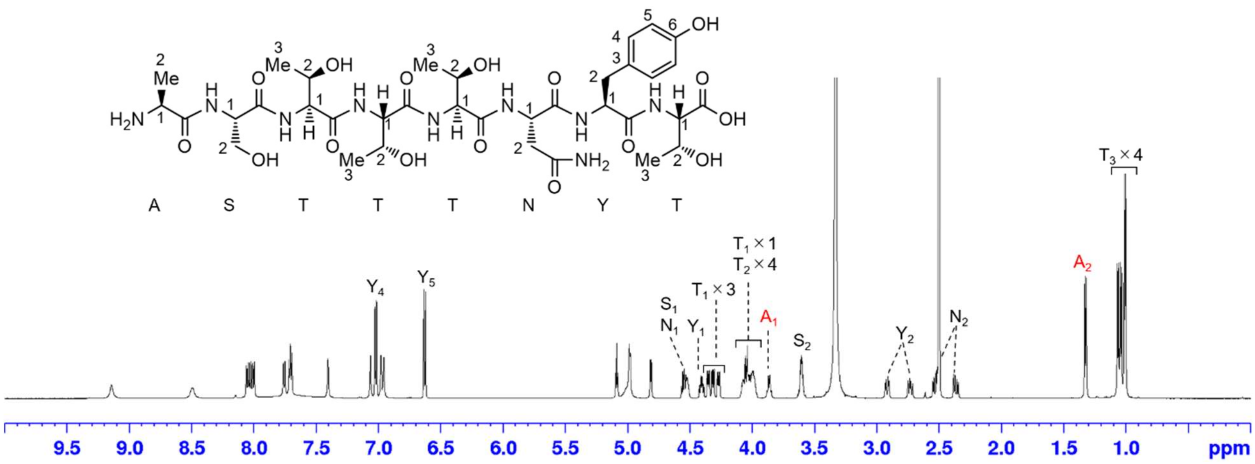

Figure S2. ^{1}H NMR spectra of native peptide T (**1**). The number above each signal in ^{1}H NMR spectra corresponded to protons attached to the carbon with the same number.

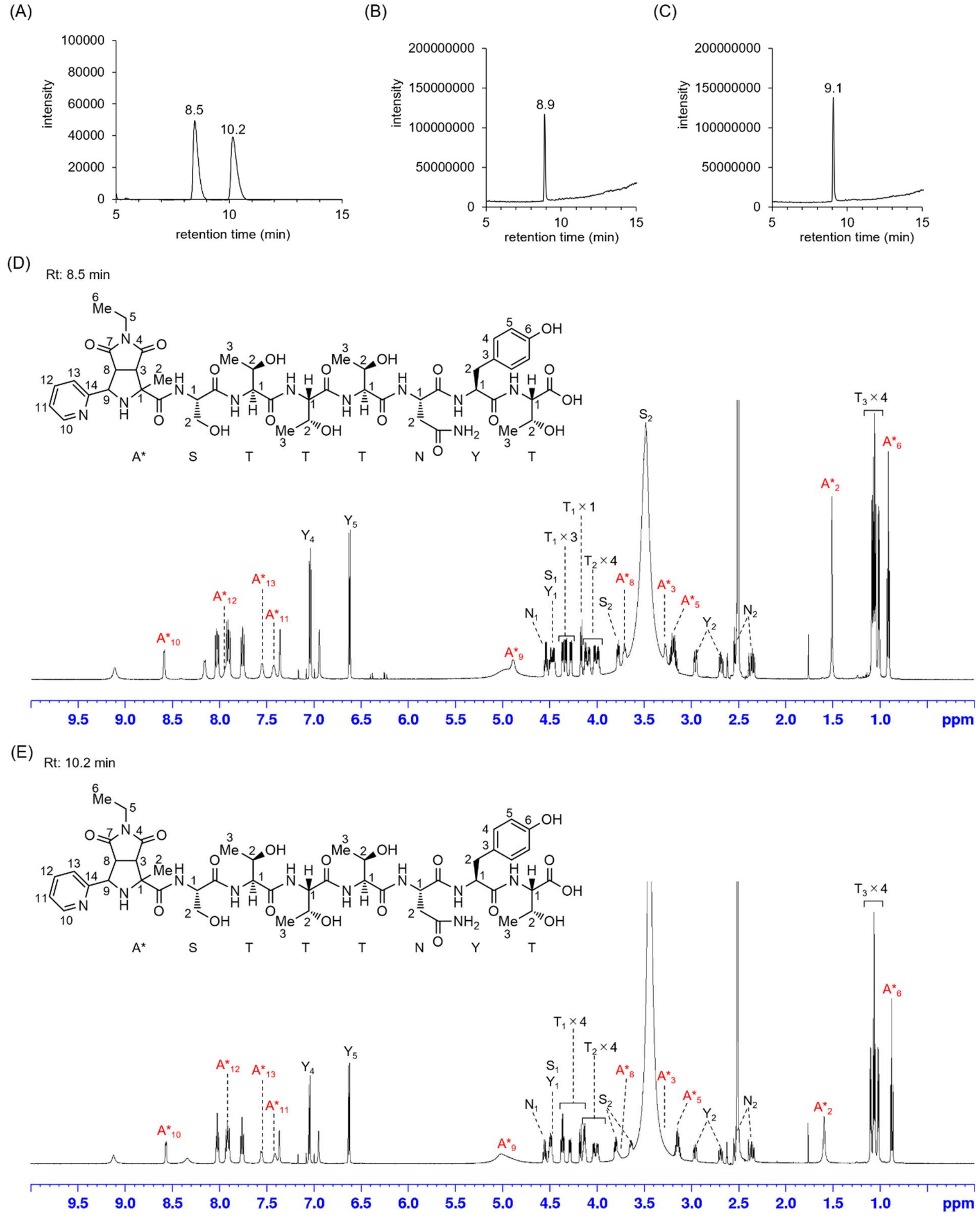

Figure S3. (A) Preparative HPLC chromatogram of the crude reaction mixture of of **1** with **2a** and **3a**. Reaction conditions: Peptide T (**1**, 0.2 mM), **2a** (2 mM), **3a** (2 mM), $Cu(OAc)_2$ (2 mM) in phosphate buffer (10 mM, pH 6.0) at 37 °C for 3 h, then EDTA (4 mM) and methoxyamine (40 mM). (B) LC-MS analysis of the fraction at 8.5 min in the preparative HPLC chromatogram. (C) LC-MS analysis of the fraction at 10.2 min in the preparative HPLC chromatogram. (D)(E) ^1H NMR spectra of the

modified peptide **4aa** at retention time of 8.5 and 10.2 min in preparative HPLC. The number above each signal in ^{1}H NMR spectra corresponded to protons attached to the carbon with the same number.

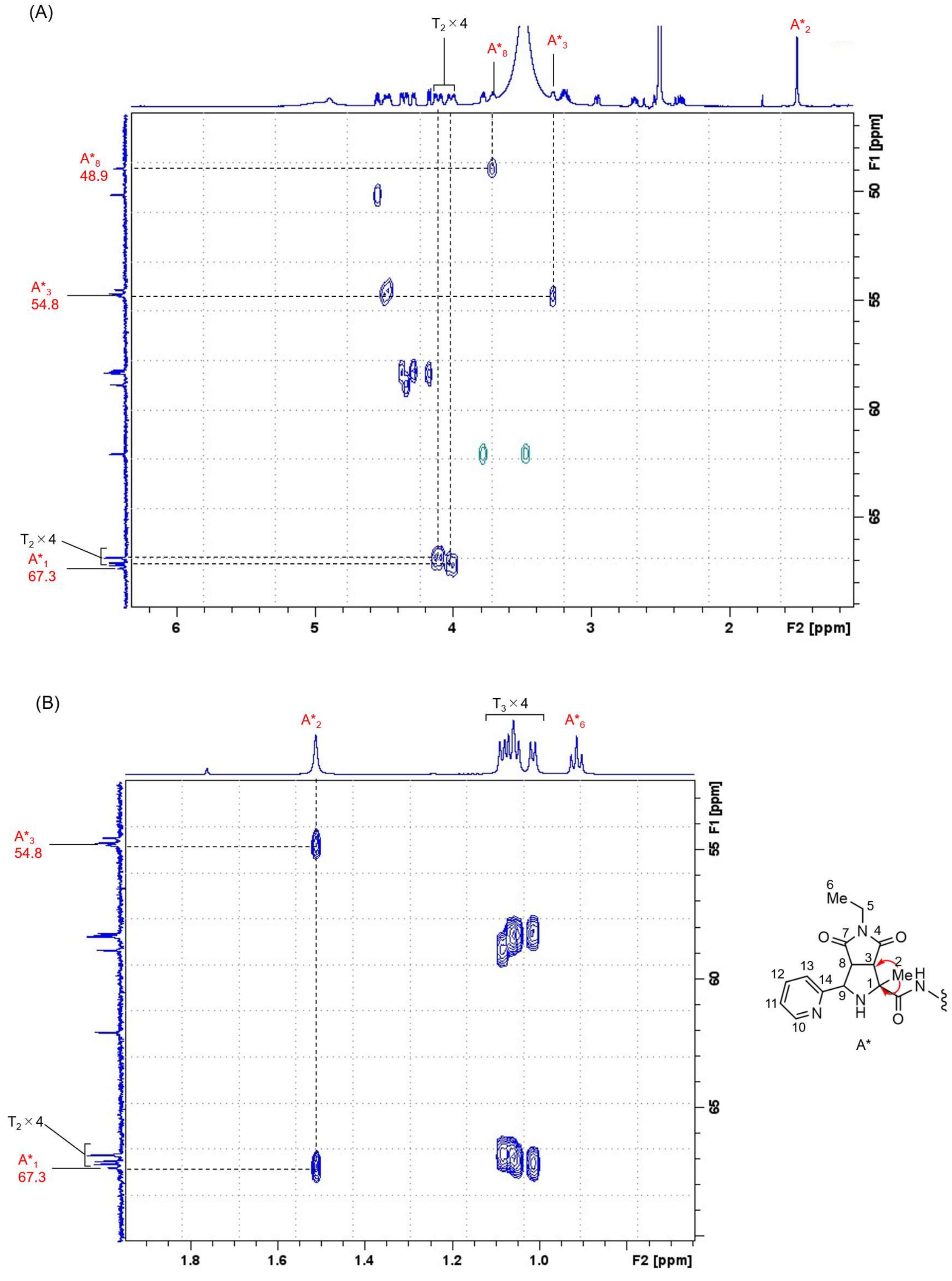

Figure S4. 2D NMR studies for structure identification of the modified peptide T **4aa** (Rt: 8.5 min). (A) HSQC and (B) HMBC spectra. X-axis: ^{1}H NMR chemical shifts (600 MHz), Y-axis: ^{13}C NMR chemical shifts (125 MHz).

Table S1 ^{1}H and ^{13}C NMR assignments for peptide T (**1**) and the modified peptide T (**4aa**)

		**1**		**4aa** (Rt: 8.5 min)		**4aa** (Rt: 10.2 min)	
	No.	^{1}H	^{13}C	^{1}H	^{13}C	^{1}H	^{13}C
A1	NH	–	–	–	–	–	–
	1	3.88–3.85	48.4	–	67.3	–	N.D
	2	1.32 (d, J = 6.9 Hz)	17.9	1.50 (s)	22.9	1.59	22.7
	3	–	–	3.27 (br)	54.8	N.D.	54.7
	4	–	–	–	175.1	–	N.D.
	5	–	–	3.23–3.15	33.5	3.19–3.11	33.5
	6	–	–	0.91 (t, J = 7.2 Hz)	13.2	0.87 (t, J = 7.2 Hz)	13.0
	7	–	–	–	175.3	–	N.D.
	8	–	–	3.71 (br)	48.9	3.78 (br)	49.1
	9	–	–	4.89 (br)	N.D.	5.01 (br)	N.D.
	10	–	–	8.59 (br)	147.6	8.57 (d, J = 4.3 Hz)	148.4
	11	–	–	7.43 (br)	124.0	7.42 (br)	124.0
	12	–	–	7.94 (br)	N.D.	7.92 (br)	N.D.
	13	–	–	7.55 (br)	122.9	7.56 (br)	123.3
	14	–	–	–	N.D.	–	N.D.
S2	NH	8.49 (br)	–	8.16 (d, J = 6.3 Hz)	–	8.34 (br)	–
	1	4.52 (br)	54.9	4.47–4.41	54.5	4.51–4.47	54.7
	2	3.62–3.58	61.9	3.79–3.76, 3.51–3.45	62.0	2.97–2.94, 2.71–2.67	62.3
T3,4,5,8	NH	8.03 (d, J = 8.3 Hz)	–	8.04 (d, J = 8.2 Hz)<br>7.92 (d, J = 8.5 Hz)<br>7.77 (d, J = 8.4 Hz)<br>7.75 (d, J = 8.5 Hz)	–	8.02 (d, J = 7.7 Hz)<br>7.93 (d, J = 8.5 Hz)<br>7.77 (d, J = 8.8 Hz)<br>7.76 (d, J = 9.1 Hz)	–
	1	4.36–4.34, 4.32–4.30<br>4.28–4.26, 4.06–4.04	58.4, 58.1<br>57.79,<br>57.76	4.37–4.35, 4.34–4.32<br>4.29–4.27, 4.18–4.16	58.8, 58.31<br>58.26, 58.2	4.38–4.36, 4.36–4.35<br>4.29–4.27, 4.18–4.16	58.6, 58.31<br>58.29, 58.2
	2	4.08–3.97	66.7, 66.6<br>66.4, 66.4	4.14–4.10, 4.10–4.06<br>4.04–4.02, 4.00–3.97	67.2, 67.0<br>66.81,<br>66.78	4.15–4.11, 4.05–4.01<br>4.01–3.97	67.1, 67.0<br>66.81, 66.78
	3	1.06 (d, J = 6.4 Hz)<br>1.04 (d, J = 6.4 Hz)<br>1.01 (d, J = 6.3 Hz)<br>1.01 (d, J = 6.3 Hz)	20.0, 19.5<br>19.32,<br>19.25	1.08 (d, J = 6.4 Hz)<br>1.06 (d, J = 6.5 Hz)<br>1.04 (d, J = 6.5 Hz)<br>1.01 (d, J = 6.4 Hz)	20.8, 20.0<br>19.74,<br>19.67	1.10 (d, J = 6.4 Hz)<br>1.07 (d, J = 6.4 Hz)<br>1.05 (d, J = 6.4 Hz)<br>1.02 (d, J = 6.5 Hz)	20.8, 20.0<br>19.8, 19.7
N6	NH	8.06 (d, J = 7.8 Hz)	–	8.02 (d, J = 8.0 Hz)	–	8.03 (d, J = 7.3 Hz)	–
	1	4.57–4.53	49.7	4.56–4.52	50.1	4.57–4.53	50.1

	2	2.55–2.51, 2.38–2.34	37	2.54–2.52, 2.36–2.32	37.6	2.55–2.52, 2.37–2.33	37.6
Y7	NH	8.00 (d, *J* = 7.8 Hz)		7.90 (d, *J* = 8.1 Hz)	–	7.91 (d, *J* = 8.1 Hz)	–
	1	4.42–4.39	54.8	4.50–4.48	54.7	4.51–4.47	55.1
	2	2.93–2.90, 2.75–2.71	36.1	2.97–2.94, 2.70–2.66	36.8	2.97–2.94, 2.71–2.67	36.8
	3	–	127.7	–	128.3	–	128.3
	4	7.02 (d, *J* = 8.5 Hz)	130.1	7.04 (d, *J* = 8.5 Hz)	130.7	7.05 (d, *J* = 8.5 Hz)	130.7
	5	6.63 (d, *J* = 8.5 Hz)	114.9	6.62 (d, *J* = 8.5 Hz)	115.3	6.63 (d, *J* = 8.5 Hz)	115.3
	6	–	155.7	–	156.2	–	156.2

Screening of reaction conditions for N-terminal modification of peptide T (1) via the copper(II)-mediated [3+2] cycloaddition

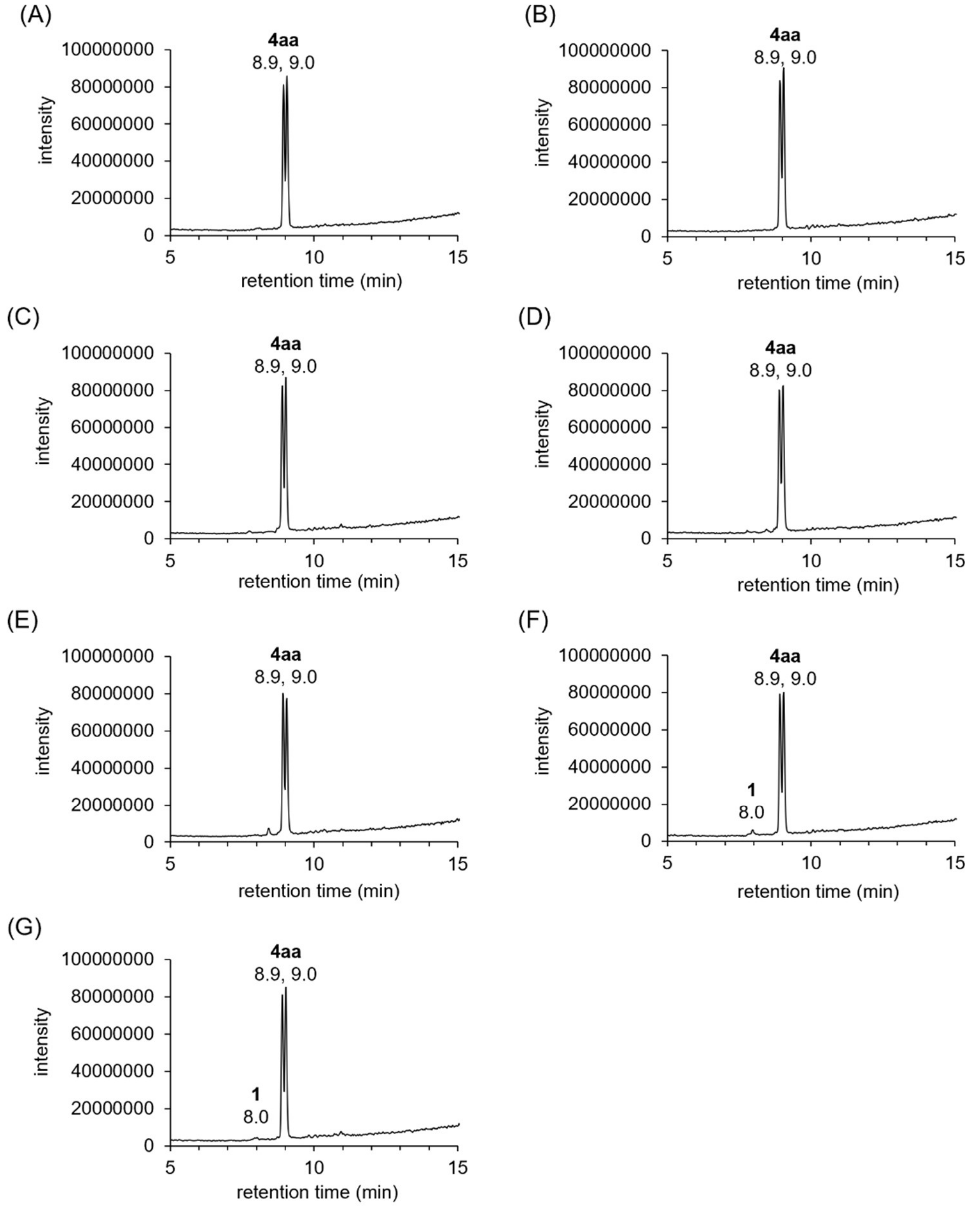

Figure S5. Total ion chromatograms in LC-MS analyses of the crude reaction mixtures of **1** with **2a** and **3a** in various buffers. (A) Phosphate buffer (10 mM, pH 6.0), (B) phosphate buffer (10 mM, pH 6.5), (C) phosphate buffer (10 mM, pH 7.0), (D) phosphate buffer (10 mM, pH 7.5), (E) phosphate buffer (10 mM, pH 8.0), (F) MES buffer (10 mM, pH 6.0), and (G) *N*-methylmorpholine buffer (10 mM, pH 6.0). Conditions: **1** (0.2 mM), **2a** (2 mM), $Cu(OAc)_2$ (2 mM), and **3a** (2 mM) in buffer at 37 °C for 3 h, then EDTA (4 mM) and methoxyamine (40 mM).

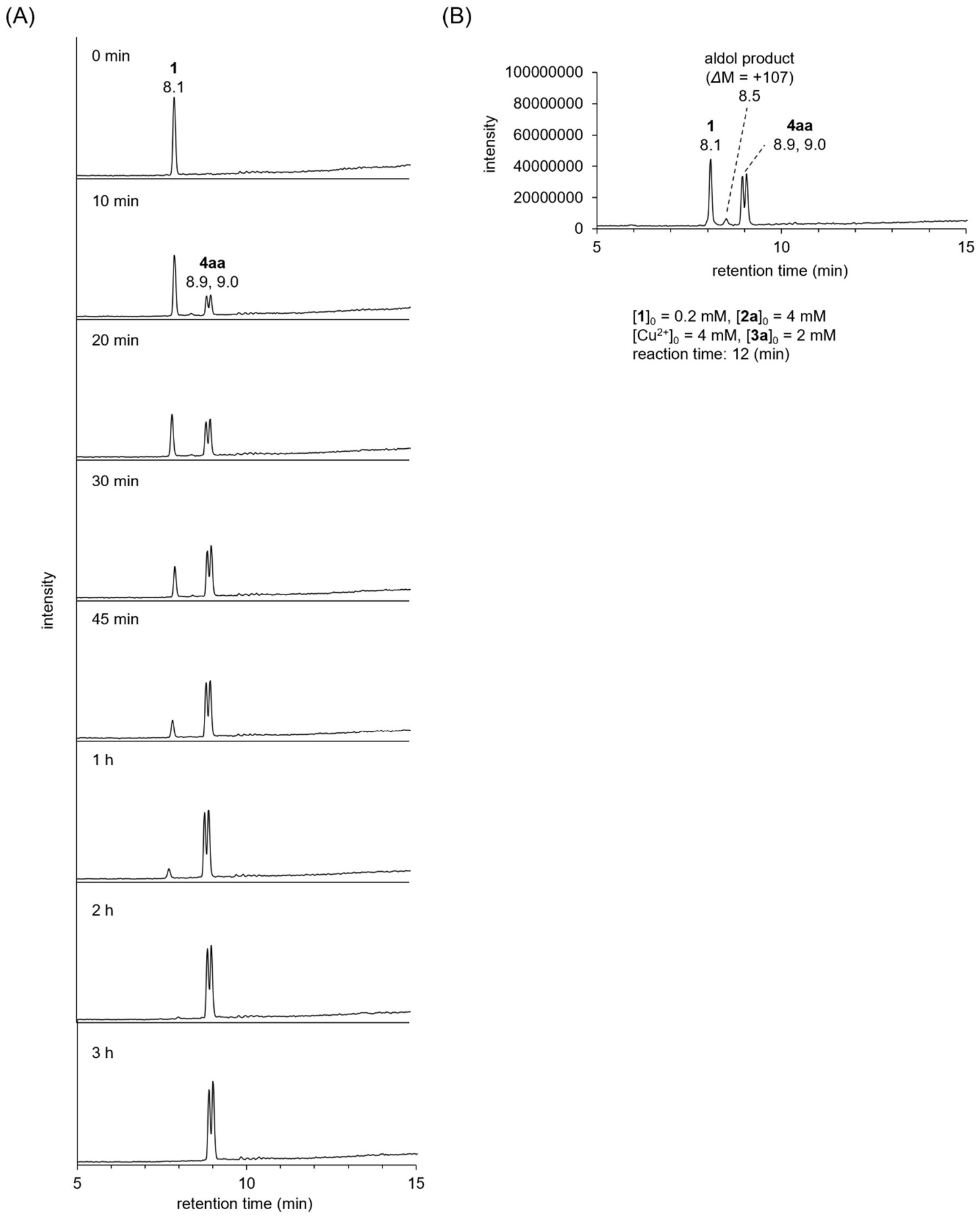

Figure S6. Kinetic study of the copper(II)-mediated N-terminal modification of **1** with **2a** and **3a**. (A) Total ion chromatograms in LC-MS analyses of the crude reaction mixtures after the incubation for a given period of time. (B) Typical LC-MS chromatogram in the case with high relative concentration of **2a**. Retention time: **1**: 8.1 min; aldol product: 8.5 min; **4aa**: 8.9 and 9.0 min.

Stability of the modified peptide T 4aa under various biologically relevant conditions

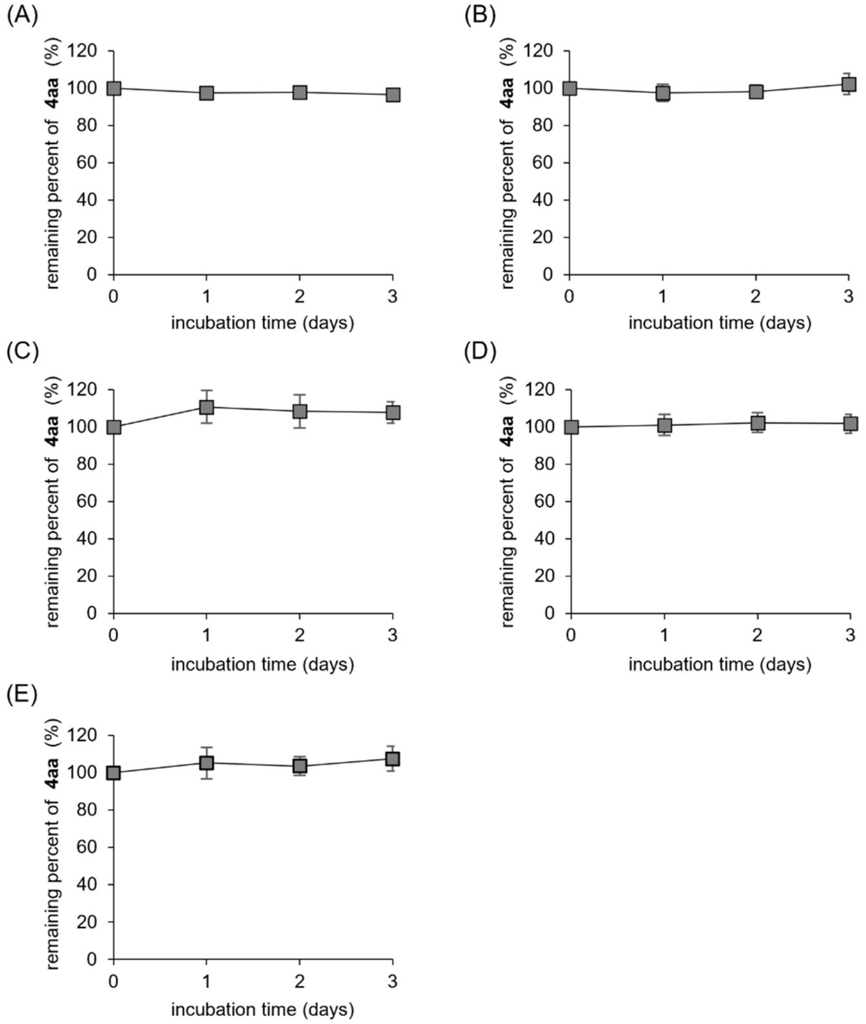

Figure S7. Remaining percent of **4aa** after incubation in (A) phosphate buffer (50mM, pH 7.4), (B) aqueous HCl solution (0.1 M), (C) glutathione (1 mM in phosphate buffer (50 mM, pH 7.4)), (D) TCEP (1 mM in phosphate buffer (50 mM, pH 7.4)), and (E) H_2O_2 (1 mM in phosphate buffer (50 mM, pH 7.4)) for a given time of period at 37 °C.

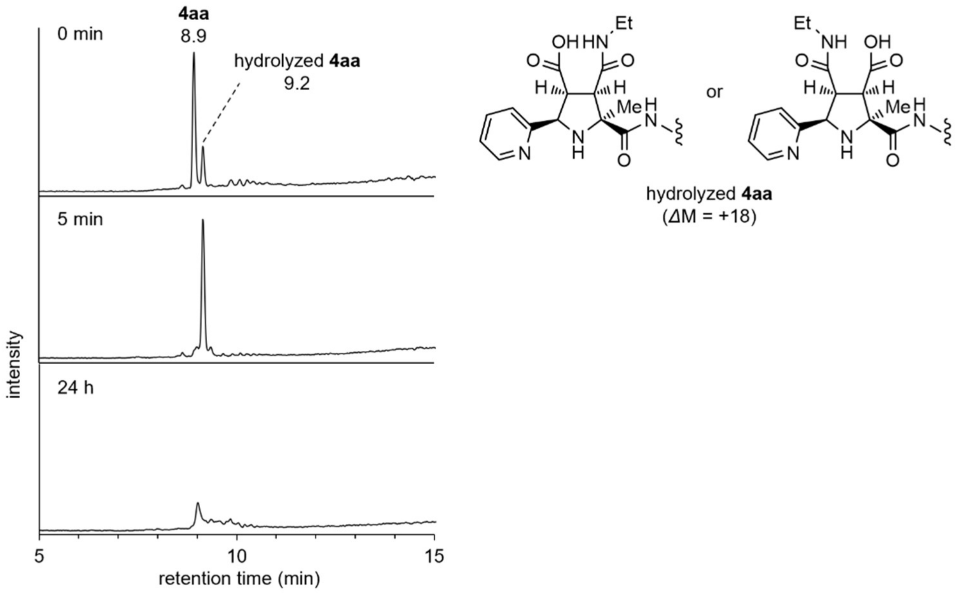

Figure S8. Total ion chromatograms in LC-MS analyses of **4aa** after incubation in aqueous NaOH solution (0.1 M) for a given time of period at 37 °C.

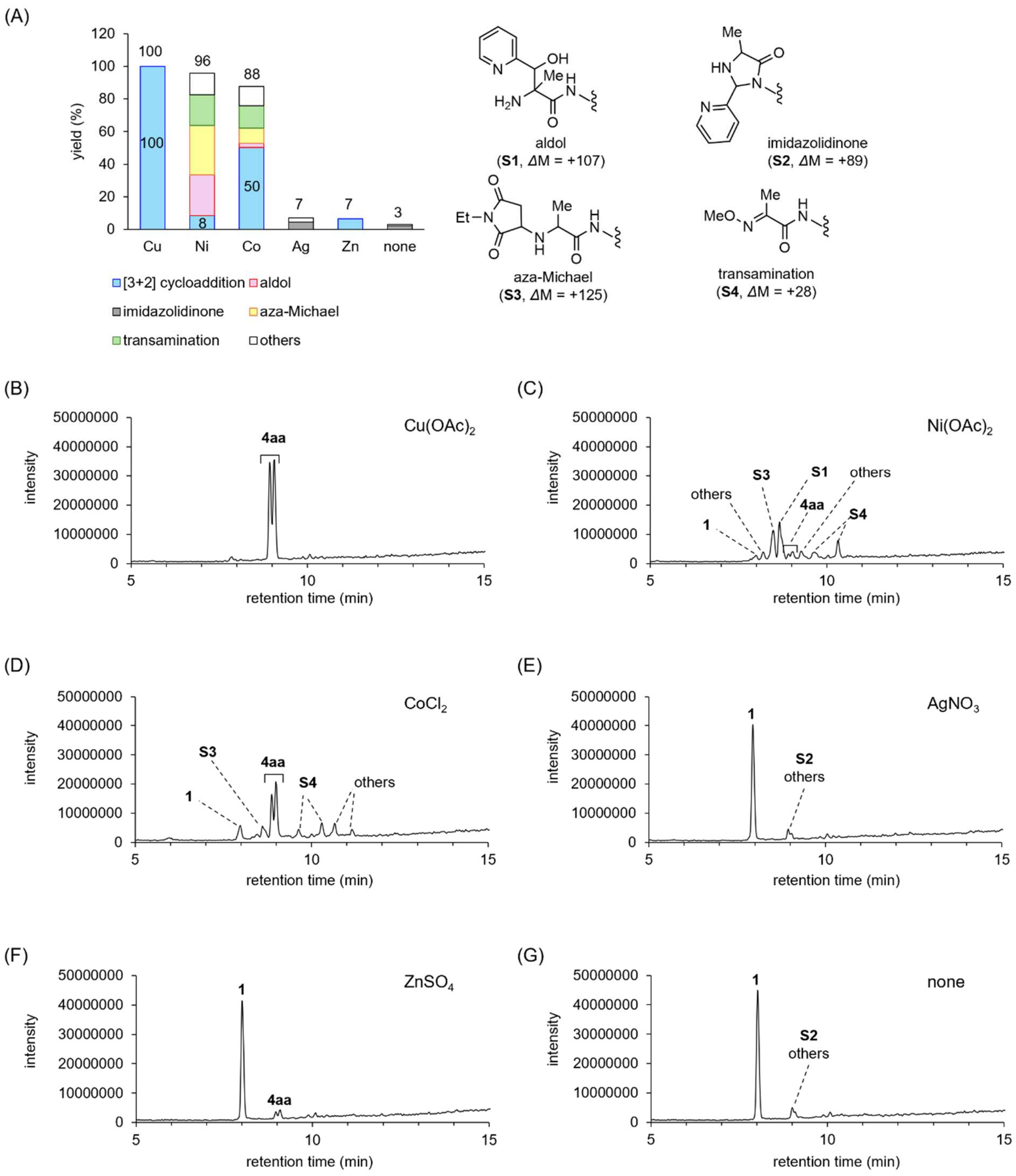

Figure S9. The reaction of **1** with **2a** and **3a** in the presence of various metal salts including $Cu(OAc)_2$, $Ni(OAc)_2$, $CoCl_2$, $AgNO_3$, and $ZnSO_4$. (A) The yields of each product were assessed by LC-MS analyses of the reaction mixture. The number above each bar represents the conversion of **1**. The number in the bar is the yield of the modified products **4aa**. (B)–(G) Total ion chromatograms in LC-MS analyses of the crude reaction mixtures of the N-terminal modification of **1** (retention time: 8.0 min) with **2a** and **3a** in the presence or absence of metal salts. Reaction condition: **1** (0.2 mM), **2a** (2 mM), **3a** (2 mM) and a metal salt (2 mM) in phosphate buffer (10 mM, pH 6.0) at 37 °C for 3 h, then EDTA (4 mM) and methoxyamine (40 mM).

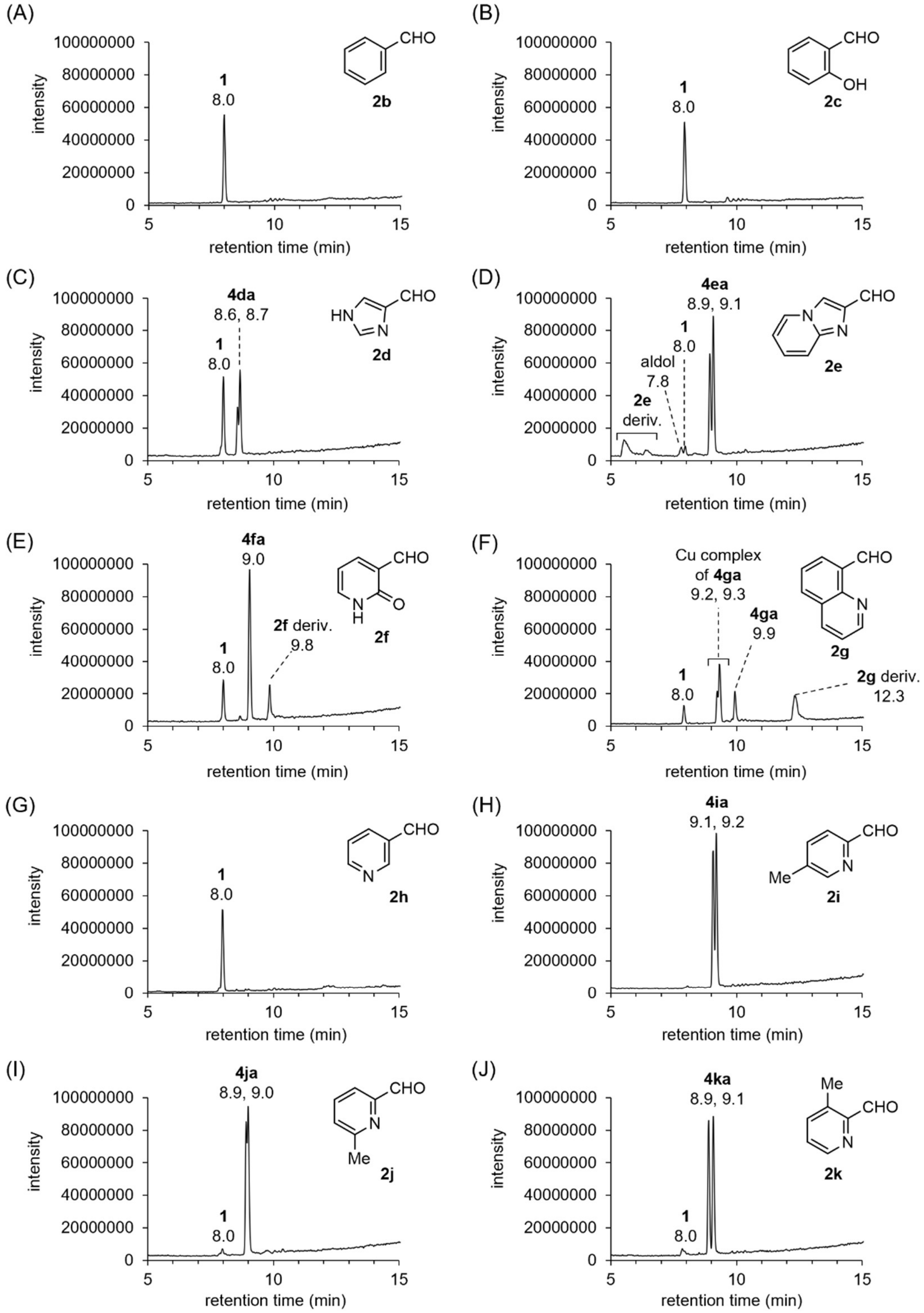

Figure S10. Total ion chromatograms in LC-MS analyses of the crude reaction mixtures of the copper(II)-mediated N-terminal modification of **1** (retention time: 8.0 min) with **2b**−**k** and **3a**. For the reaction with **2e**, "aldol" corresponded to the aldol product with ($\Delta M = +146$ amu). Reaction conditions: **1** (0.2 mM), aldehyde (2 mM), **3a** (2 mM), and $Cu(OAc)_2$ (2 mM) in phosphate buffer (10 mM, pH 6.0) at 37 °C for 3 h, then EDTA (4 mM) and methoxyamine (40 mM).

(A)

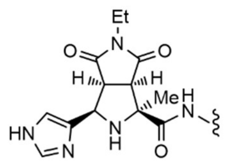

4da
calcd. for [M+H]$^{+}$: 1061.4

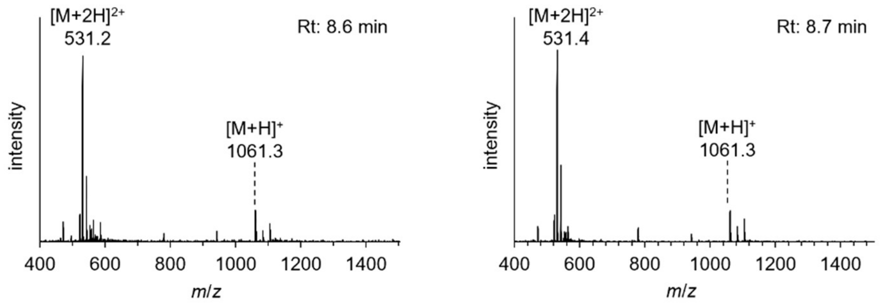

(B)

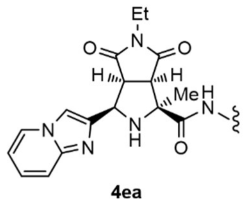

4ea
calcd. for [M+H]$^{+}$.: 1111.5

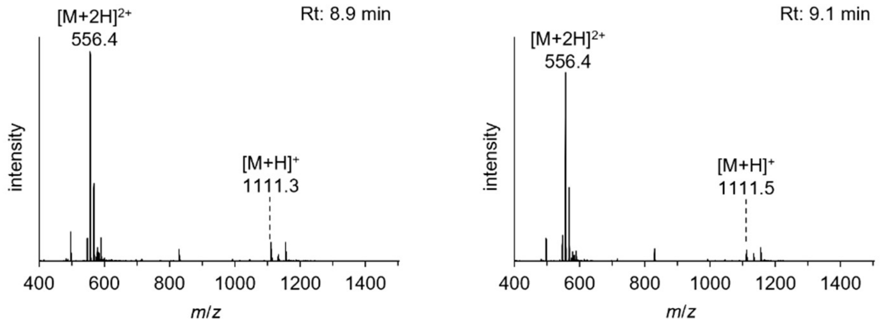

(C)

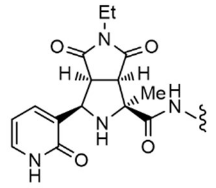

4fa
calcd. for [M+H]$^{+}$.: 1088.4

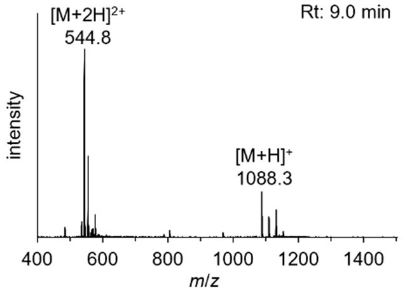

(D)

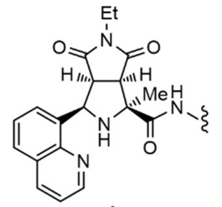

4ga
calcd. for [M+H]$^{+}$.: 1122.5
calcd. for [M+Cu−H]$^{+}$.: 1183.4

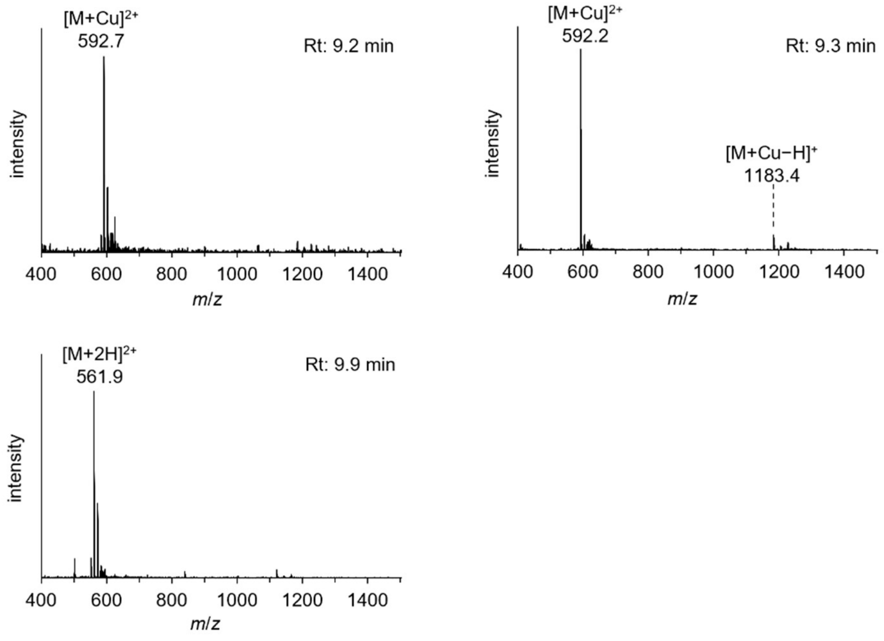

(E)

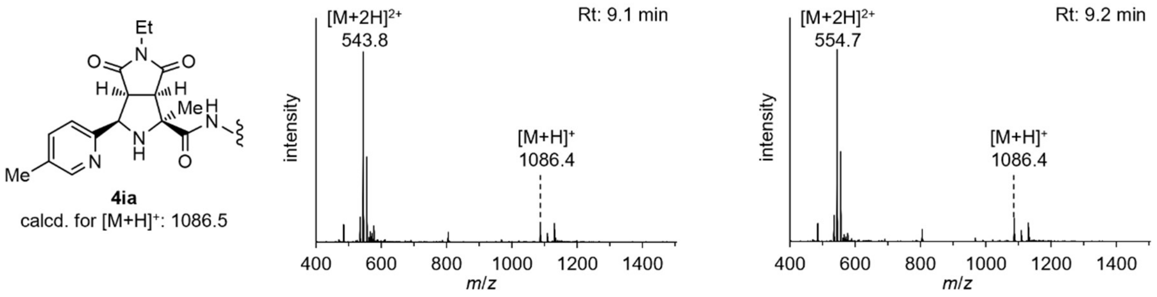

(F)

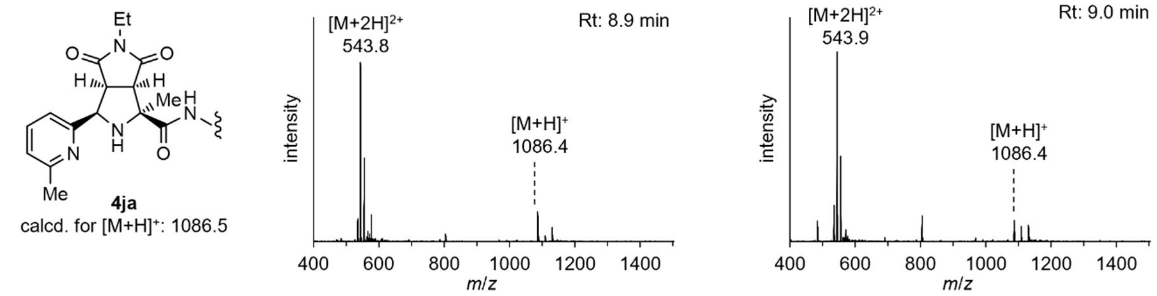

(G)

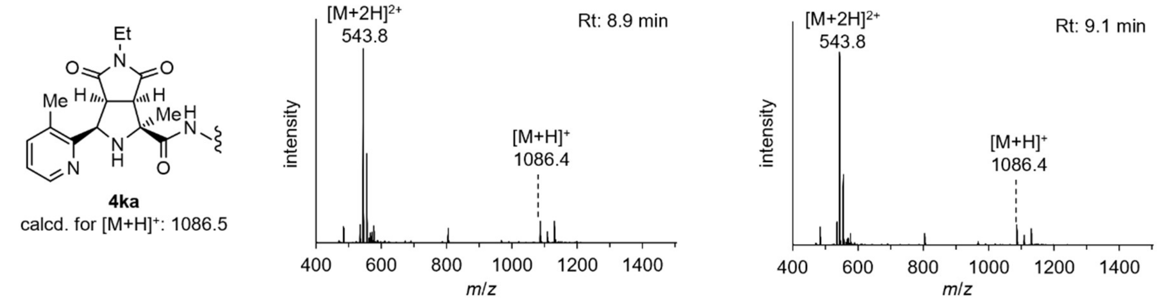

Figure S11. Mass spectra for peaks of **4xa** in total ion chromatograms (Figure S10) in LC-MS analyses of the crude reaction mixtures of **1** with various arylaldehyde and **3a**. Rt: retention time in the LC-MS analysis.

Table S2. Scope of dipolarophile on N-terminal modification of peptide T (**1**)

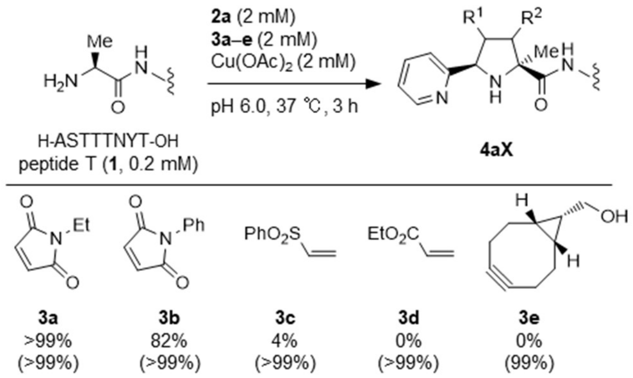

Conversion of peptide T (**1**) is shown in parentheses.

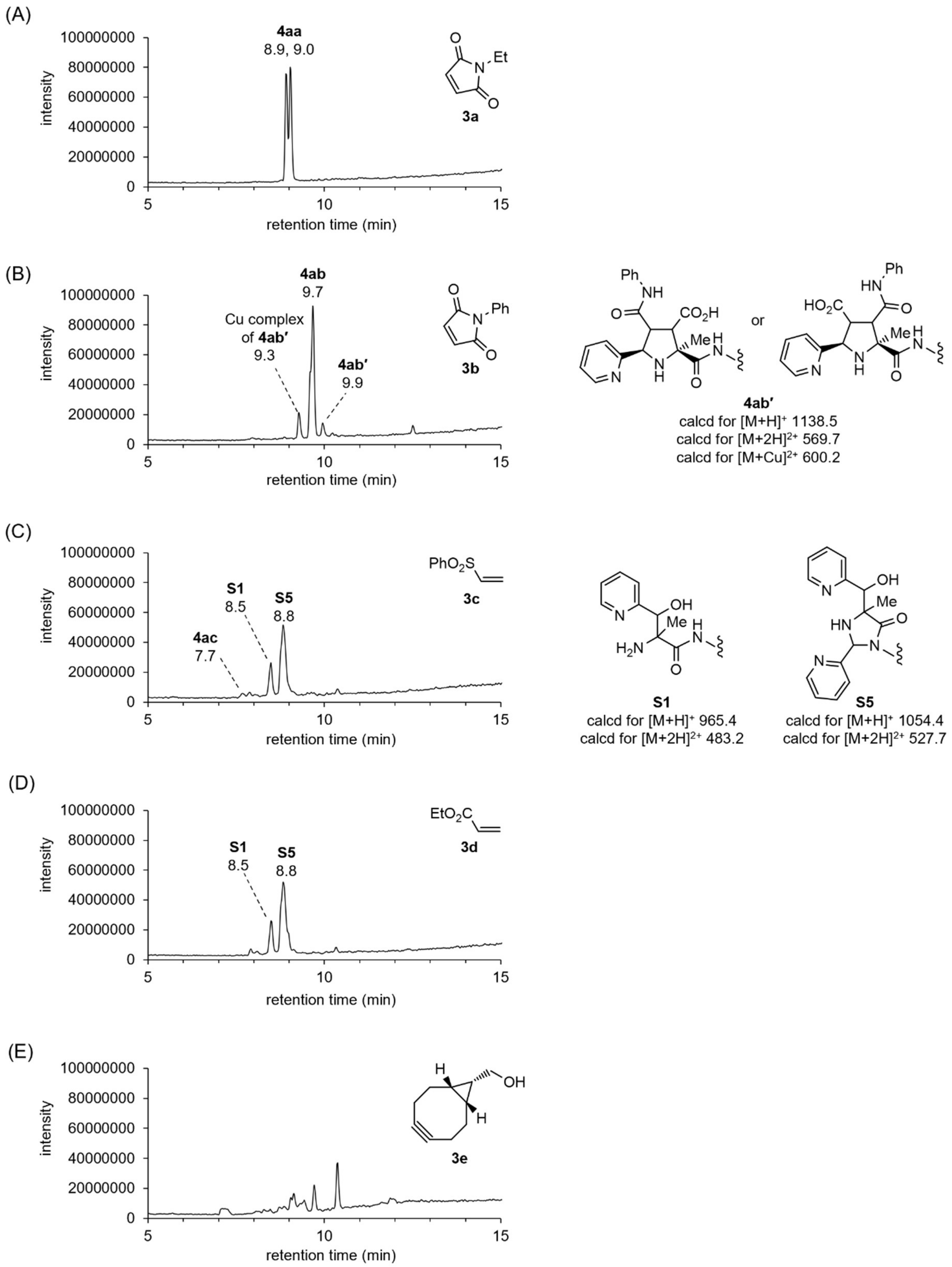

Figure S12. Total ion chromatograms in LC-MS analyses of the crude reaction mixtures of the copper(II)-mediated N-terminal modification of **1** (retention time: 8.0 min) with **2a** and **3a**–**e**. Reaction conditions: **1** (0.2 mM), **2a** (2 mM), **3a**–**e** (2 mM), and $Cu(OAc)_2$ (2 mM) in phosphate buffer (10 mM, pH 6.0) at 37 °C for 3 h, then EDTA (4 mM) and methoxyamine (40 mM).

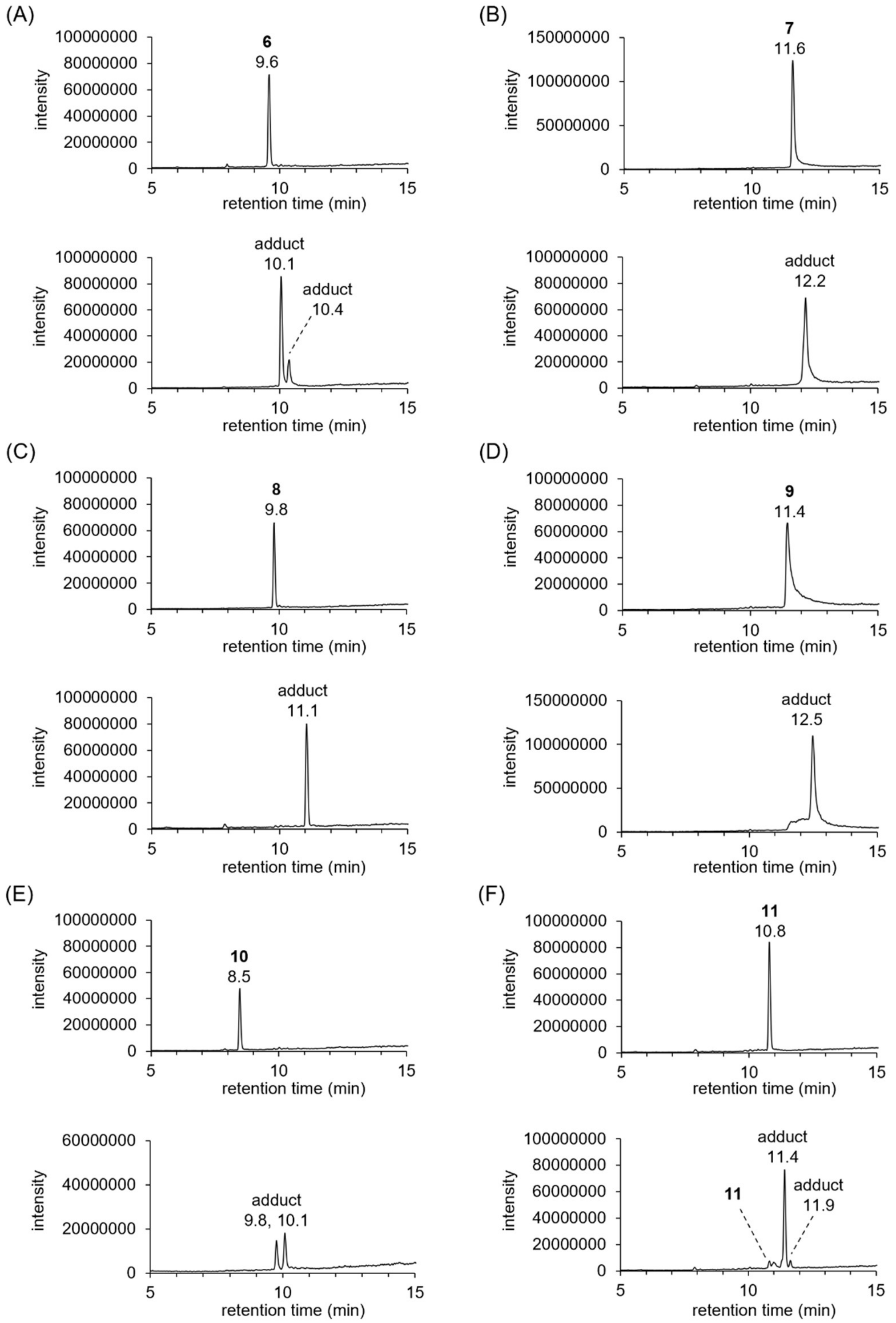

Figure S13. Total ion chromatograms in LC-MS analyses of biologically peptides **6–11** (top) and the crude reaction mixtures of the copper(II)-mediated N-terminal modification of **6–11** with **2a** and **3a** (bottom). Reaction conditions: peptide (0.2 mM), **2a** (2 mM), **3a** (2 mM), and $Cu(OAc)_2$ (2 mM) in phosphate buffer (10 mM, pH 6.0) at 37 °C for 3 h, then EDTA (4 mM) and methoxyamine (40 mM).

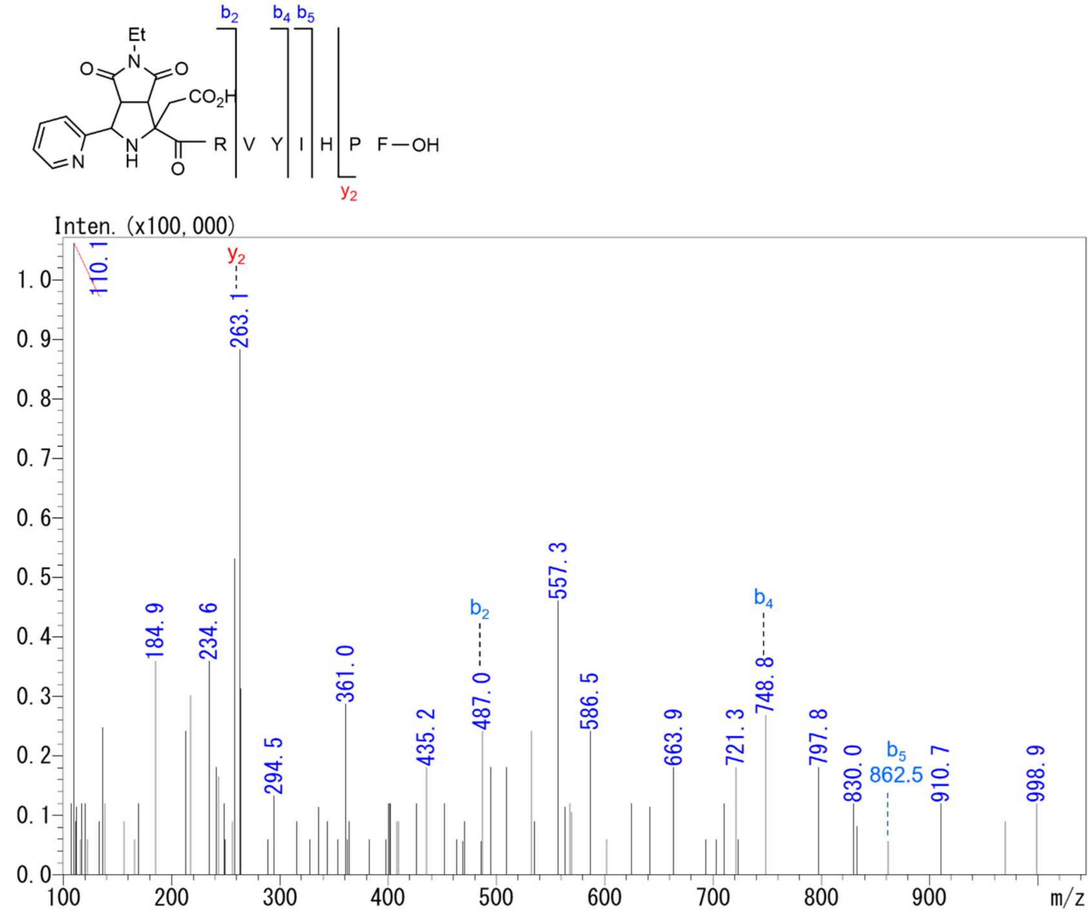

Figure S14. Collision-induced dissociation (CID) and the LC-MS/MS spectrum of the doubly charged positive ion at m/z = 631.10 corresponding to the adduct of angiotensin II (**6**).

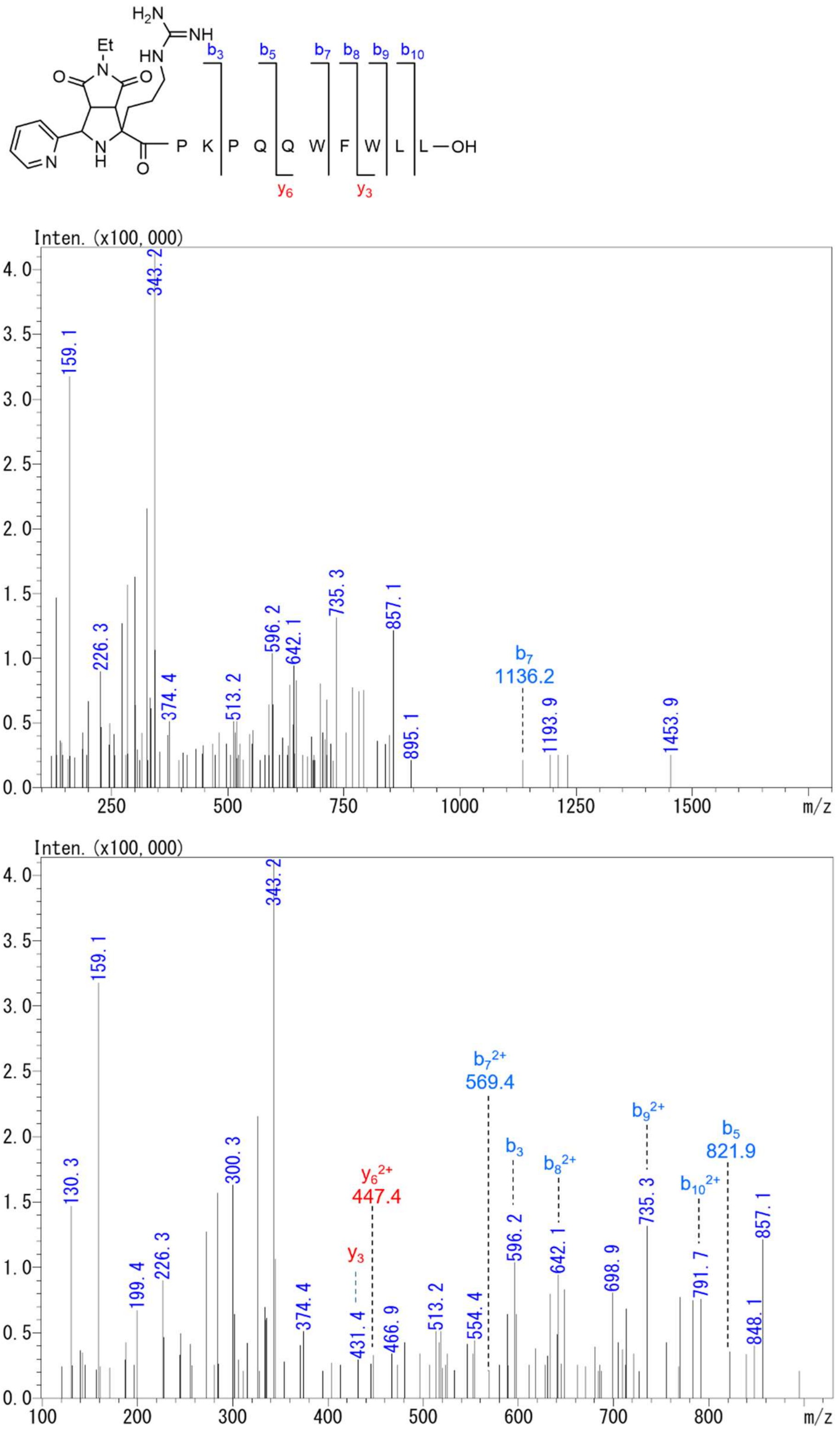

Figure S15. Collision-induced dissociation (CID) and the LC-MS/MS spectrum of the doubly charged positive ion at *m*/*z* = 856.75 corresponding to the adduct of substance P (**7**). The bottom mass spectrum was an enlarged view.

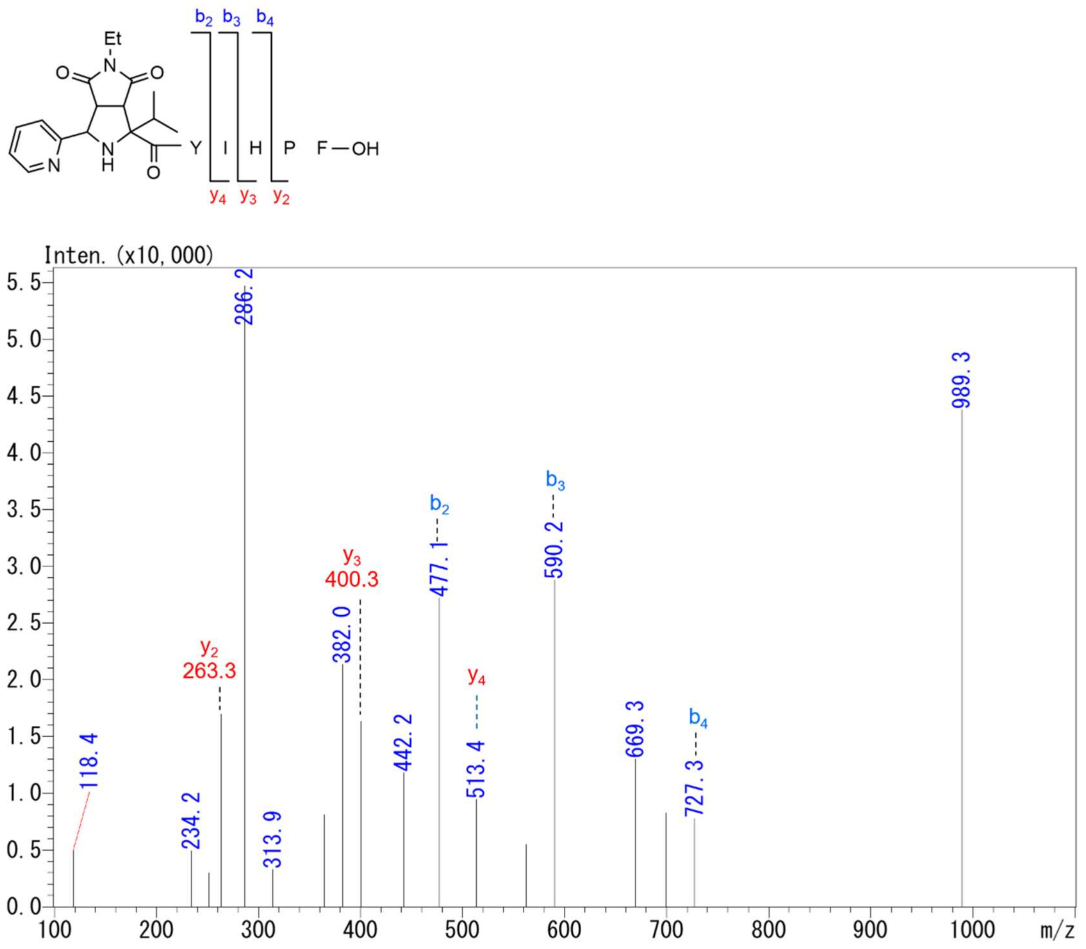

Figure S16. Collision-induced dissociation (CID) and the LC-MS/MS spectrum of the singly charged positive ion at *m*/*z* = 989.40 corresponding to the adduct of angiotensin IV (**8**).

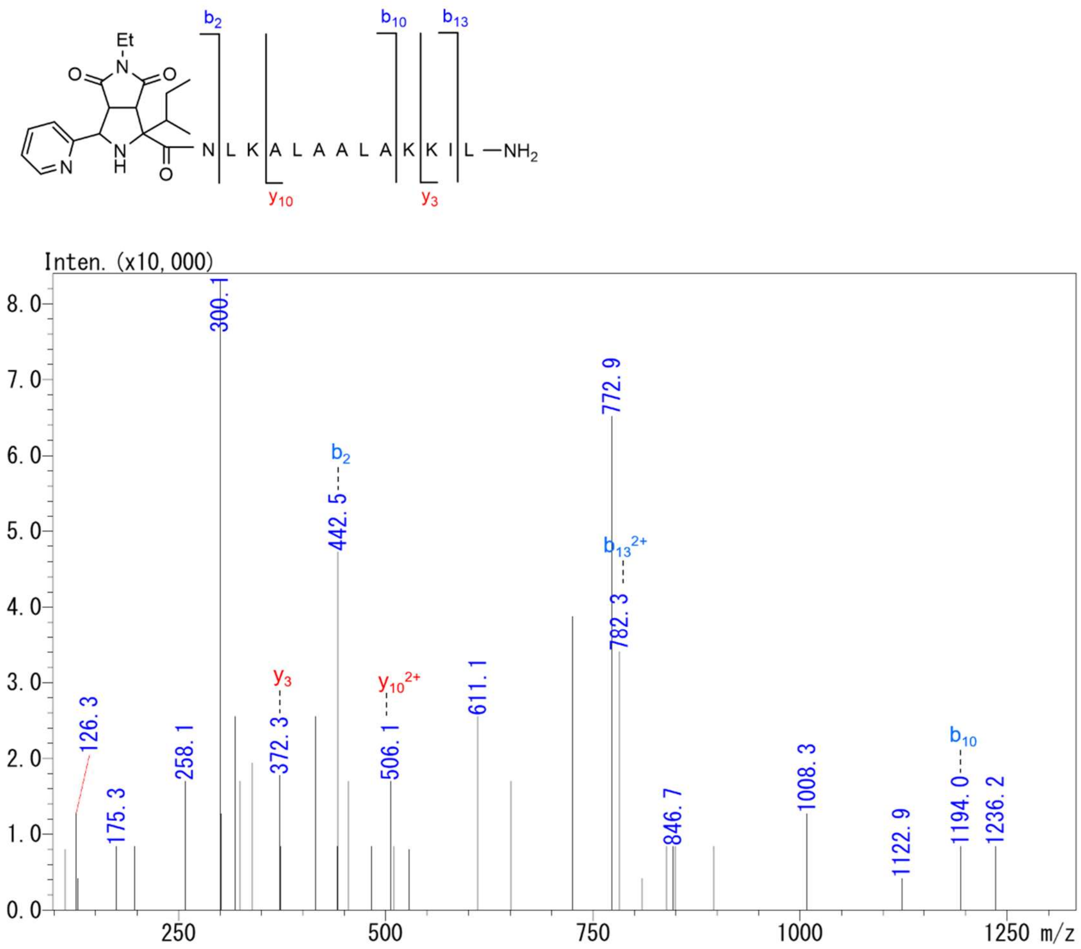

Figure S17. Collision-induced dissociation (CID) and the LC-MS/MS spectrum of the doubly charged positive ion at *m*/*z* = 847.30 corresponding to the adduct of mastoparan (**9**).

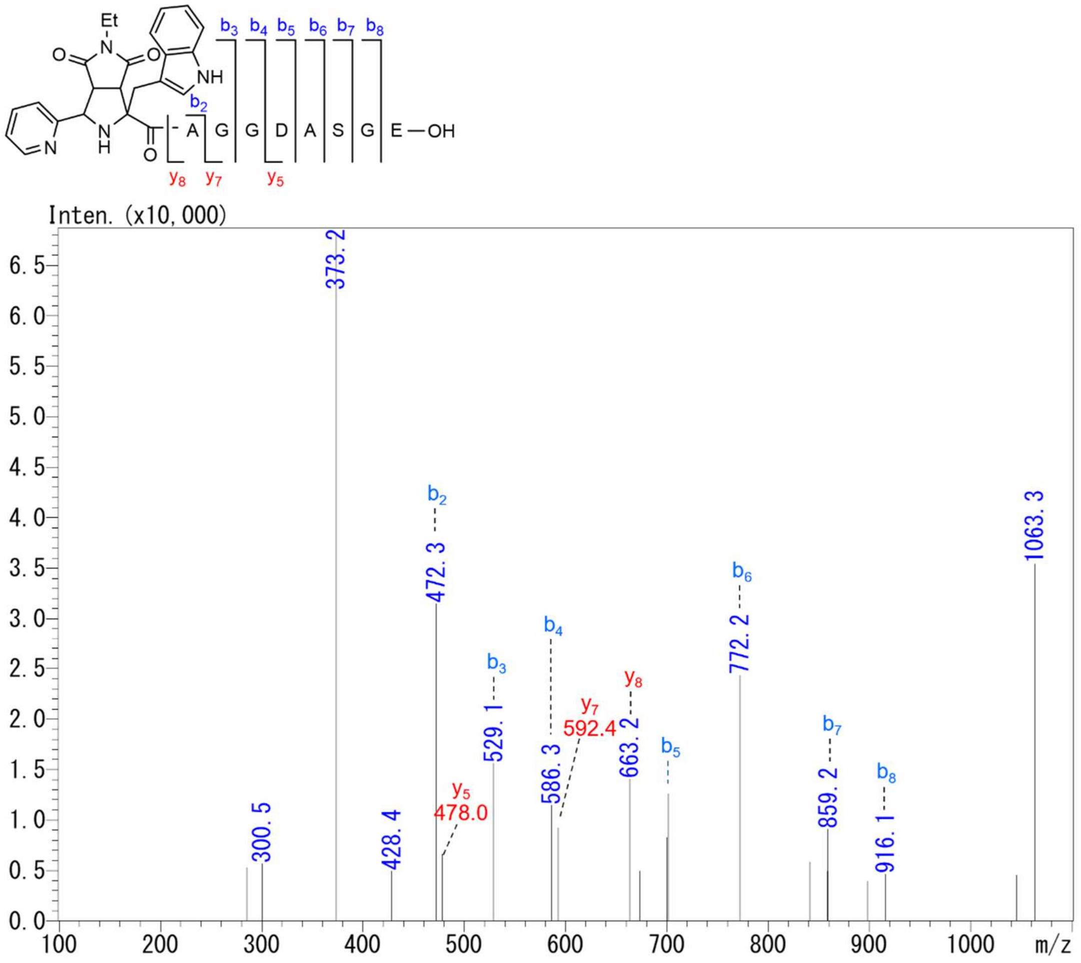

Figure S18. Collision-induced dissociation (CID) and the LC-MS/MS spectrum of the singly charged positive ion at *m*/*z* = 1063.3 corresponding to the adduct of delta sleep-inducing peptide (**10**).

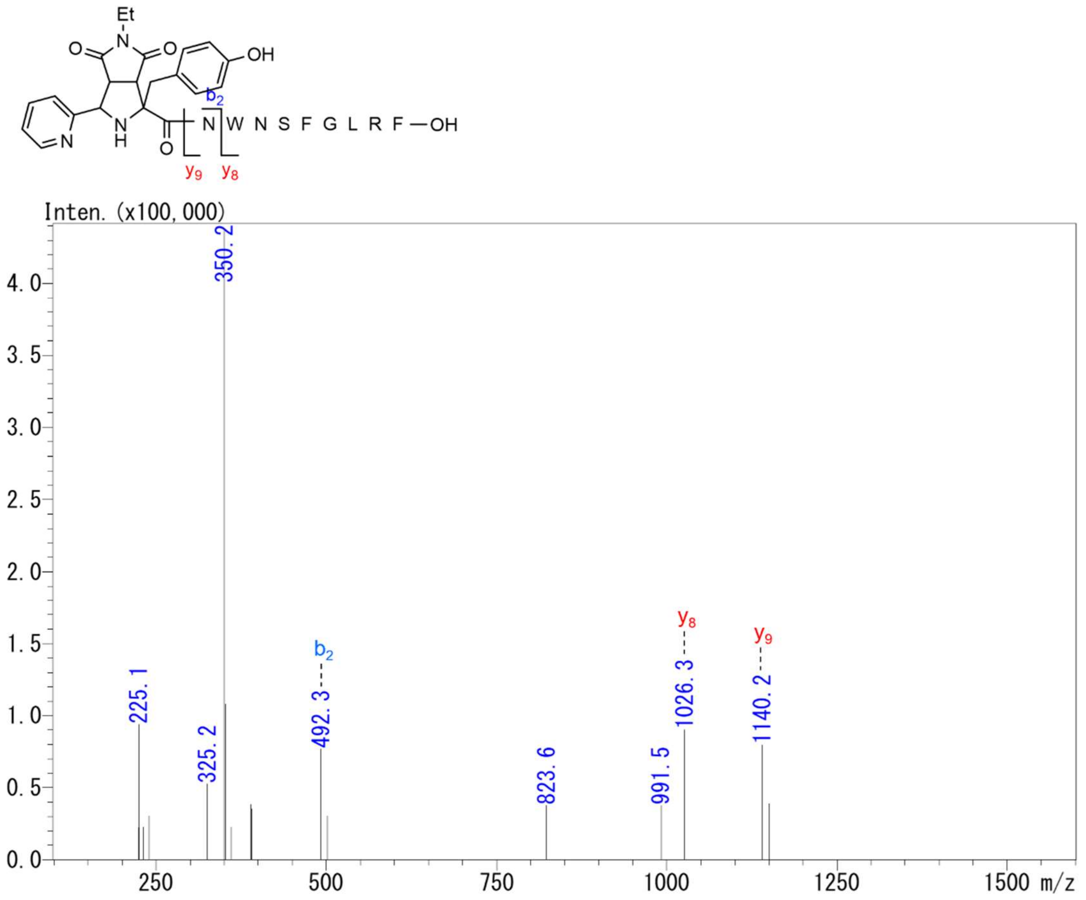

Figure S19. Collision-induced dissociation (CID) and the LC-MS/MS spectrum of the doubly charged positive ion at m/z = 759.15 corresponding to the adduct of kisspeptin-10 (**11**).

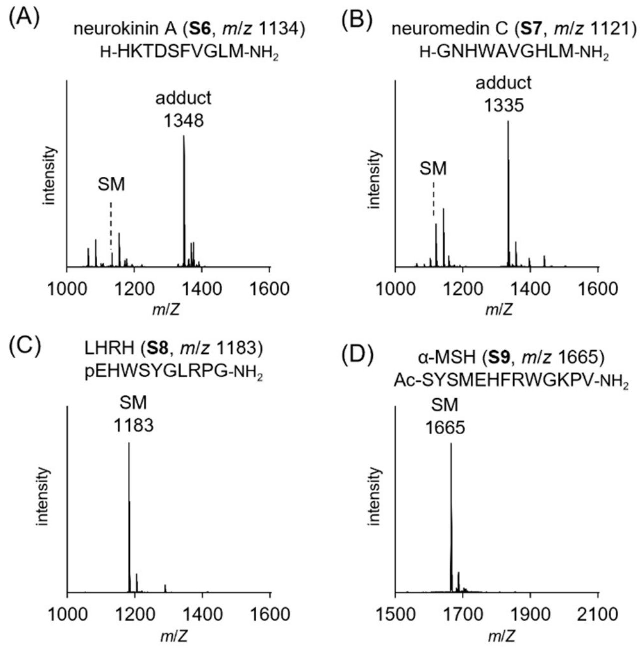

Figure S20. MALDI TOF-MS spectra of copper(II)-mediated [3+2] cycloaddition of peptides **S6**–**S9** with **2a** and **3a**. Conditions: peptides (0.2 mM), **2a** (2 mM), **3a** (2 mmol), and $Cu(OAc)_2$ (2 mM) in phosphate buffer (10 mM, pH 6.0) at 37 °C for 6 h, then EDTA (4 mM) and methoxyamine (40 mM).

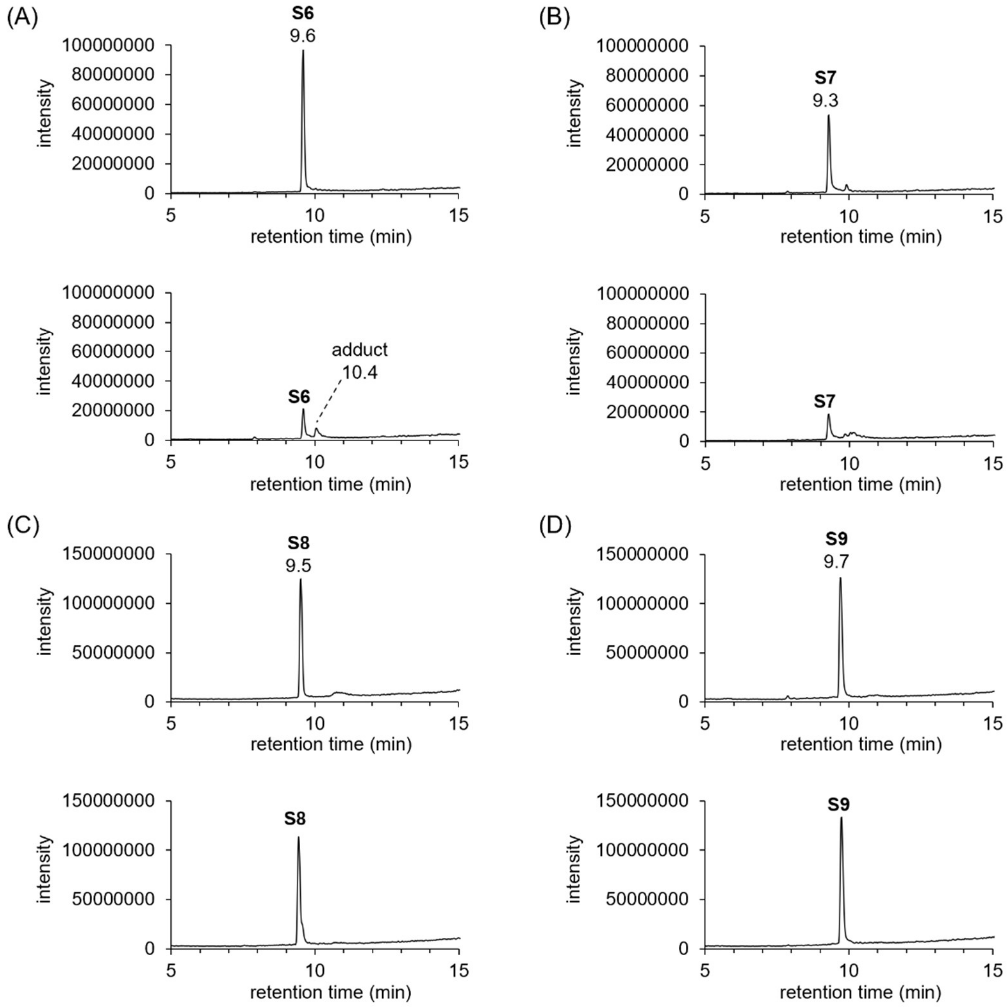

Figure S21. Total ion chromatograms in LC-MS analyses of biologically peptides **S6**–**S9** (top) and the crude reaction mixtures of the copper(II)-mediated N-terminal modification of **S6**–**S9** with **2a** and **3a** (bottom). Reaction conditions: peptide (0.2 mM), **2a** (2 mM), **3a** (2 mM), and $Cu(OAc)_2$ (2 mM) in phosphate buffer (10 mM, pH 6.0) at 37 °C for 6 h, then EDTA (4 mM) and methoxyamine (40 mM)

LC-MS analyses of N-terminal modification of proteins with 2a and 3a

(A)

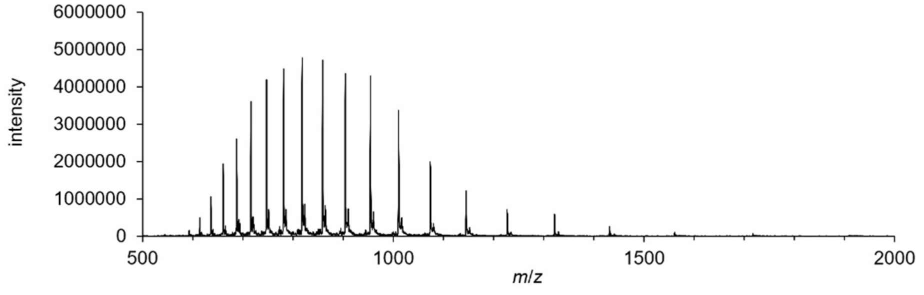

(B)

reconstructed mass (U)	number of peaks used for deconvolution	intensity	standard deviation
17163.5027	10	2590126	1
8581.2654	5	2979376	0.3
5720.7326	3	2998238	0.3
2860.4423	2	3421940	0
3432.4393	2	3277464	0
15448.0631	3	1810166	4.7
2145.3416	2	2228387	0
6006.1199	2	2454912	0.6
1906.8874	2	1983329	0.1
2451.9158	2	2211196	0
13896.7219	2	2282137	2.7
15354.0003	2	2313744	2.1
8128.427	2	2152273	1.3
6238.8814	2	1774078	1.9
16261.1909	2	2261698	1.8
16211.8836	2	2150509	2.5
15255.844	2	2221803	0.1
9701.8482	2	2213026	0.1
18176.9574	3	1328556	3.7
19186.129	2	1868850	3.8
17270.2824	6	633032.1	1.8
9297.517	2	1869028	1.2
1430.3427	2	1343434	0.1
5493.4657	2	1468688	1.5
6177.999	2	1361496	0.5
1320.2406	2	778093	0.1
19305.2388	2	1153382	3.2
8634.3353	4	571220.1	0.4
18238.4531	2	1057126	2.7
9153.9073	2	704397.6	0.5
7625.0463	2	595687.1	2.1
5755.9963	2	671507.5	0
3453.508	2	590518	0.1
1225.9846	2	348595.8	0
2878.2298	2	554124.3	0.3
9008.6298	2	518437.9	0.3
2158.7327	2	391647.9	0.3
7969.5969	2	375218.9	0.8
8978.9657	2	339725	0
16192.592	2	287607.9	0.2
17223.6912	2	246477.8	4

Figure S22. (A) Mass spectrum of the crude reaction mixture of the copper(II)-mediated N-terminal modification of myoglobin, which was used for obtaining the deconvoluted mass spectrum. (B) The list of reconstructed masses calculated using multivalent ions.

(A)

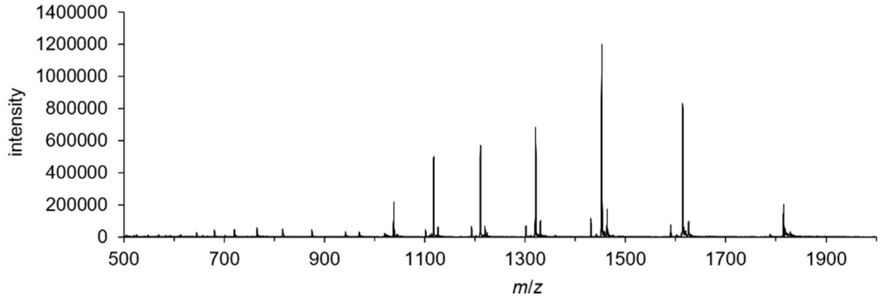

(B)

reconstructed mass (U)	number of peaks used for deconvolution	intensity	standard deviation
14517.91	7	580811.9	0.8
14624.66	5	99285.5	0.6
14303.55	4	75872.5	1.3
6501.218	2	64963.69	1.2
13403.56	2	258029.8	2.1
15840.85	2	338426	2.2
17161.08	2	341743	2.5

Figure S23. (A) Mass spectrum of the crude reaction mixture of the copper(II)-mediated N-terminal modification of lysozyme, which was used for obtaining the deconvoluted mass spectrum. (B) The list of reconstructed masses calculated using multivalent ions.

(A)

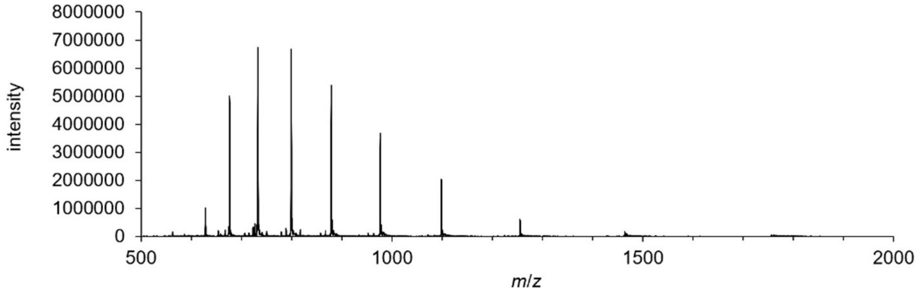

(B)

reconstructed mass (U)	number of peaks used for deconvolution	intensity	standard deviation
8777.961	8	3830209	0.3
8798.435	4	499461.4	1.7
7981.261	2	3462729	1.3

Figure S24. (A) Mass spectrum of the crude reaction mixture of the copper(II)-mediated N-terminal modification of ubiquitin, which was used for obtaining the deconvoluted mass spectrum. (B) The list of reconstructed masses calculated using multivalent ions.

(A)

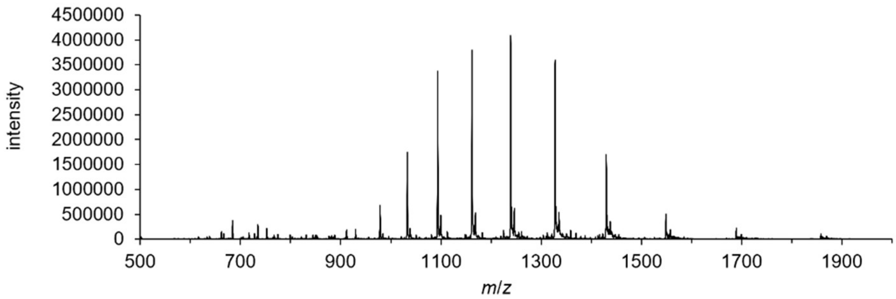

(B)

reconstructed mass (U)	number of peaks used for deconvolution	intensity	standard deviation
18574.85	9	2160108	0.9
18681.3	6	438586.5	2.5
18618.08	3	264337.5	0.6
7515.015	2	292832.8	0.1
19854.59	2	338427.5	3.8
20120.37	2	416926.8	1
2933.782	2	388571.7	1
20755.13	2	1524014	4.2
17246.75	2	1877949	1.5

Figure S25. (A) Mass spectrum of the crude reaction mixture of the copper(II)-mediated N-terminal modification of β-lactoglobulin A, which was used for obtaining the deconvoluted mass spectrum. (B) The list of reconstructed masses calculated using multivalent ions.

(A)

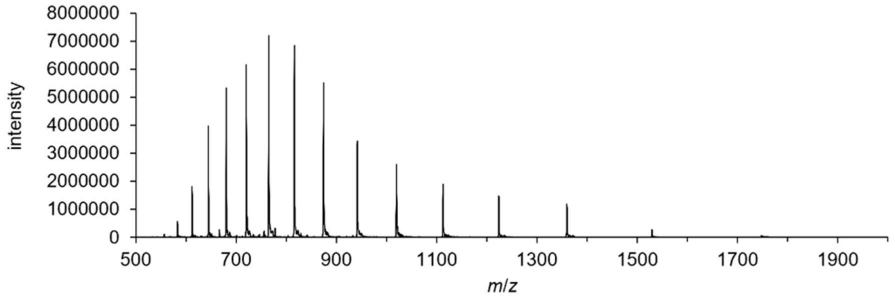

(B)

reconstructed mass	number of peaks used for deconvolution	intensity	standard deviation
12228.8317	11	3896381.84	0.6
12244.0653	2	374348.38	0.7
4076.226	2	2775161.8	0.2
1746.9524	2	2206195.85	0.1
6114.7104	2	3461683.38	0.2
13039.5081	2	2808582.25	3.8
2445.7195	2	3745820.97	0.2
3057.285	2	4045302.79	0.1

Figure S26. (A) Mass spectrum of the crude reaction mixture of the copper(II)-mediated N-terminal modification of cytochrome C, which was used for obtaining the deconvoluted mass spectrum. (B) The list of reconstructed masses calculated using multivalent ions.

Identification of modification site by MALDI-TOF MS analyses of enzyme-digested peptide fragments

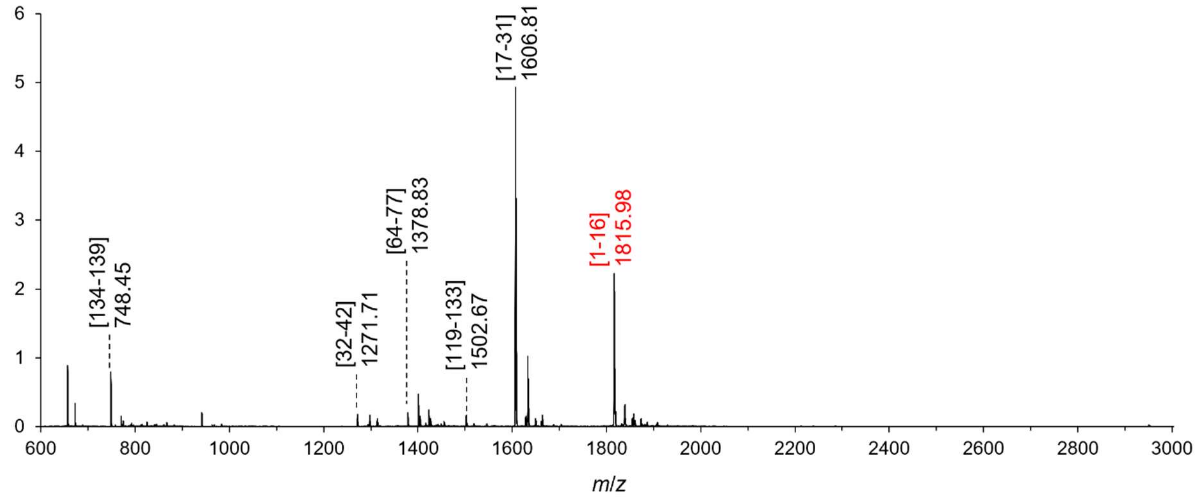

fragment	simulated m/z	charge	sequence
[1-16]	1815.9024	1	.GLSDGEWQQVLNVWGK.v
[17-31]	1606.8547	1	k.VEADIAGHGQEVLIR.I
[32-42]	1271.663	1	r.LFTGHPETLEK.f
[51-56]	708.3233	1	k.TEAEMK.a
[57-62]	662.3355	1	k.ASEDLK.k
[64-77]	1378.8417	1	k.HGTVVLTALGGILK.k
[80-96]	1853.9617	1	k.GHHEAELKPLAQSHATK.h
[103-118]	1885.0218	1	k.YLEFISDAIIHVLHSK.h
[119-133]	1502.6693	1	k.HPGDFGADAQGAMTK.a
[134-139]	748.4352	1	k.ALELFR.n
[140-145]	631.341	1	r.NDIAAK.y
[148-153]	650.3144	1	k.ELGFQG.

Figure S27. MALDI-TOF MS spectrum and the list of trypsin-digested peptide fragments of myoglobin. Fragments found in MALDI-TOF MS are highlighted in yellow in the list.

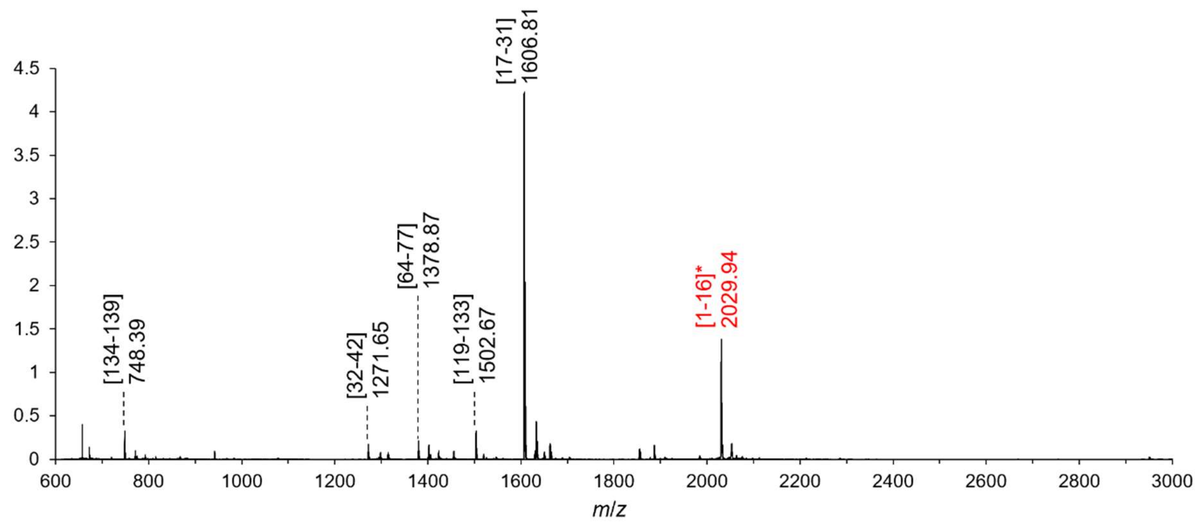

fragment	simulated m/z	charge	sequence
[1-16]	2029.9767	1	.GLSDGEWQQVLNVWGK.v [1x**2a**_1x**3a**]
[17-31]	1606.8547	1	k.VEADIAGHGQEVLIR.l
[32-42]	1271.663	1	r.LFTGHPETLEK.f
[51-56]	708.3233	1	k.TEAEMK.a
[57-62]	662.3355	1	k.ASEDLK.k
[64-77]	1378.8417	1	k.HGTVVLTALGGILK.k
[80-96]	1853.9617	1	k.GHHEAELKPLAQSHATK.h
[103-118]	1885.0218	1	k.YLEFISDAIIHVLHSK.h
[119-133]	1502.6693	1	k.HPGDFGADAQGAMTK.a
[134-139]	748.4352	1	k.ALELFR.n
[140-145]	631.341	1	r.NDIAAK.y
[148-153]	650.3144	1	k.ELGFQG.

Figure S28. MALDI-TOF MS spectrum and the list of trypsin-digested peptide fragments of the adduct of myoglobin with **2a** and **3a**. Fragments found in MALDI-TOF MS are highlighted in yellow in the list.

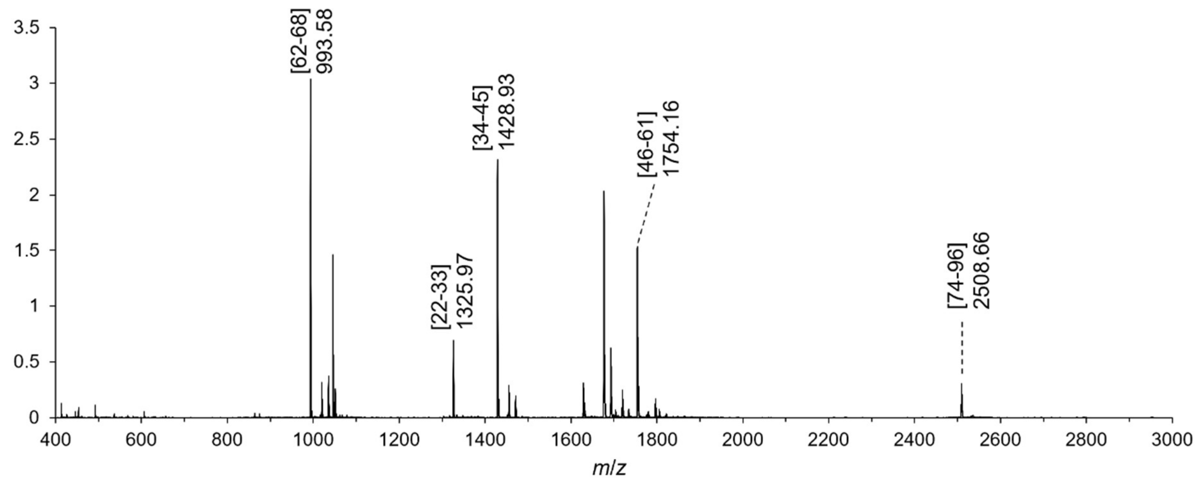

fragment	simulated m/z	charge	sequence
[2-5]	478.2772	1	k.VFGR.c
[6-13]	893.4219	1	r.CELAAAMK.r [1xCarbamidomethyl]
[15-21]	874.4166	1	r.HGLDNYR.g
[22-33]	1325.6307	1	r.GYSLGNWVCAAK.f [1xCarbamidomethyl]
[34-45]	1428.6502	1	k.FESNFNTQATNR.n
[46-61]	1753.8351	1	r.NTDGSTDYGILQINSR.w
[62-68]	993.3996	1	r.WWCNDGR.t [1xCarbamidomethyl]
[69-73]	517.2729	1	r.TPGSR.n
[74-96]	2508.1891	1	r.NLCNIPCSALLSSDITASVNCAK.k [3xCarbamidomethyl]
[98-112]	1675.8009	1	k.IVSDGNGMNAWVAWR.n
[117-125]	1045.5425	1	k.GTDVQAWIR.g

Figure S29. MALDI-TOF MS spectrum and the list of trypsin-digested peptide fragments of lysozyme. Fragments found in MALDI-TOF MS are highlighted in yellow in the list.

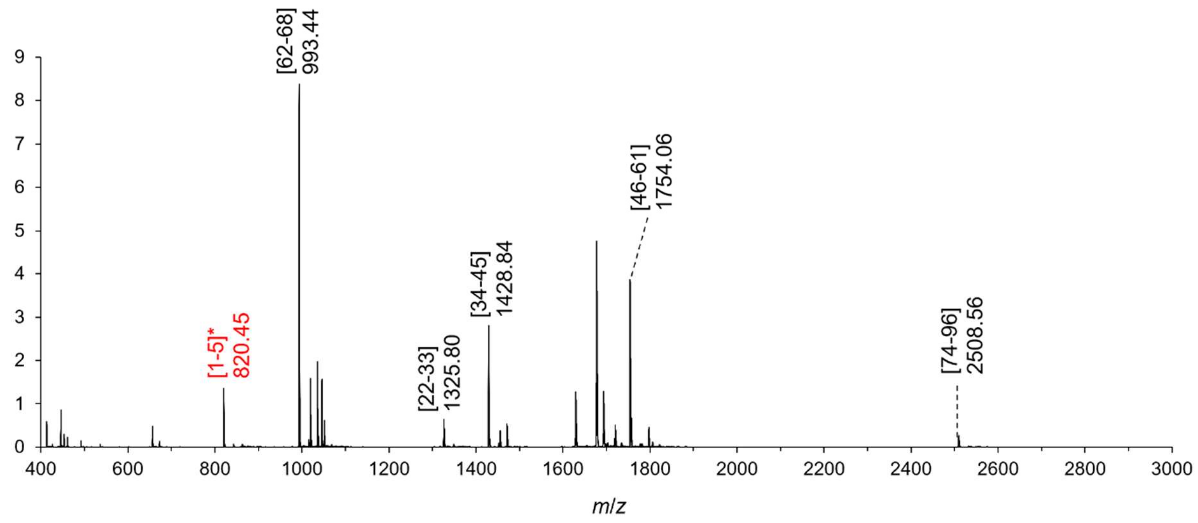

fragment	simulated m/z	charge	sequence
[1-5]	820.4464	1	.KVFGR.c[1x**2a**_1x**3a**]
[6-13]	893.4219	1	r.CELAAAMK.r [1xCarbamidomethyl]
[15-21]	874.4166	1	r.HGLDNYR.g
[22-33]	1325.6307	1	r.GYSLGNWVCAAK.f [1xCarbamidomethyl]
[34-45]	1428.6502	1	k.FESNFNTQATNR.n
[46-61]	1753.8351	1	r.NTDGSTDYGILQINSR.w
[62-68]	993.3996	1	r.WWCNDGR.t [1xCarbamidomethyl]
[69-73]	517.2729	1	r.TPGSR.n
[74-96]	2508.1891	1	r.NLCNIPCSALLSSDITASVNCAK.k [3xCarbamidomethyl]
[98-112]	1675.8009	1	k.IVSDGNGMNAWVAWR.n
[117-125]	1045.5425	1	k.GTDVQAWIR.g

Figure S30. MALDI-TOF MS spectrum and the list of trypsin-digested peptide fragments of the adduct of lysozyme with **2a** and **3a**. Fragments found in MALDI-TOF MS are highlighted in yellow in the list. Modification at N-terminus prevent tryptic cleavage at Lys1.

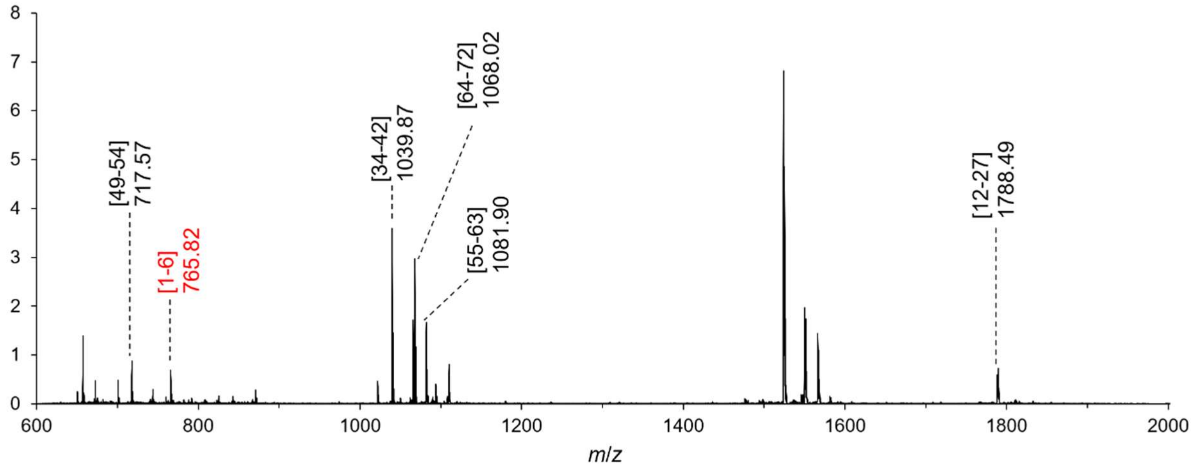

fragment	simulated m/z	charge	sequence
[1-6]	765.4328	1	.MQIFVK.t
[7-11]	519.3137	1	k.TLTGK.t
[12-27]	1787.9273	1	k.TITLEVEPSDTIENVK.a
[30-33]	503.2824	1	k.IQDK.e
[34-42]	1039.5167	1	k.EGIPPDQQR.l
[43-48]	648.4079	1	r.LIFAGK.q
[49-54]	717.3526	1	k.QLEDGR.t
[55-63]	1081.5524	1	r.TLSDYNIQK.e
[64-72]	1067.6208	1	k.ESTLHLVLR.l

Figure S31. MALDI-TOF MS spectrum and the list of trypsin-digested peptide fragments of ubiquitin. Fragments found in MALDI-TOF MS are highlighted in yellow in the list.

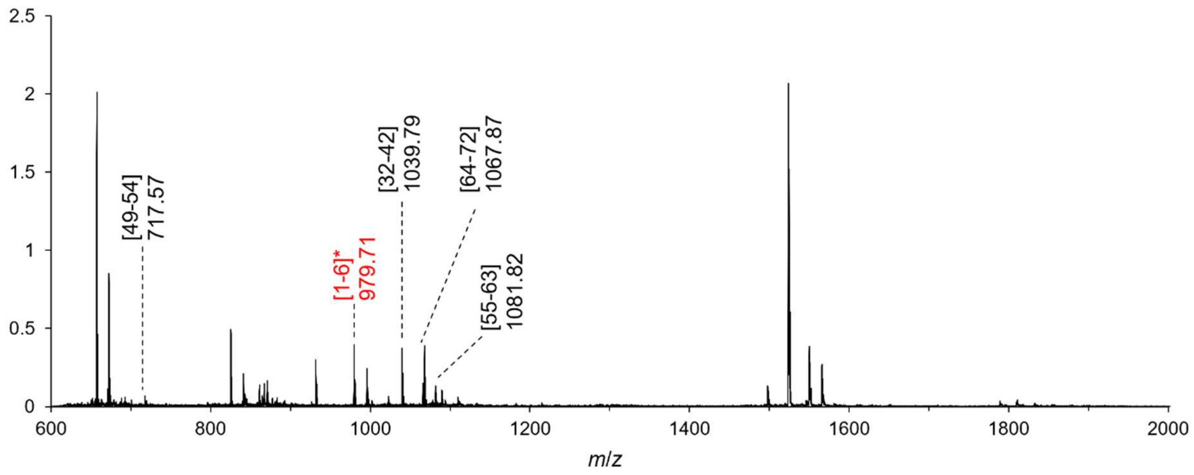

fragment	simulated m/z	charge	sequence
[1-6]	979.507	1	.MQIFVK.t [1x**2a**_1x**3a**]
[7-11]	519.3137	1	k.TLTGK.t
[12-27]	1787.9273	1	k.TITLEVEPSDTIENVK.a
[30-33]	503.2824	1	k.IQDK.e
[34-42]	1039.5167	1	k.EGIPPDQQR.l
[43-48]	648.4079	1	r.LIFAGK.q
[49-54]	717.3526	1	k.QLEDGR.t
[55-63]	1081.5524	1	r.TLSDYNIQK.e
[64-72]	1067.6208	1	k.ESTLHLVLR.l

Figure S32. MALDI-TOF MS spectrum and the list of trypsin-digested peptide fragments of the adduct of ubiquitin with **2a** and **3a**. Fragments found in MALDI-TOF MS are highlighted in yellow in the list.

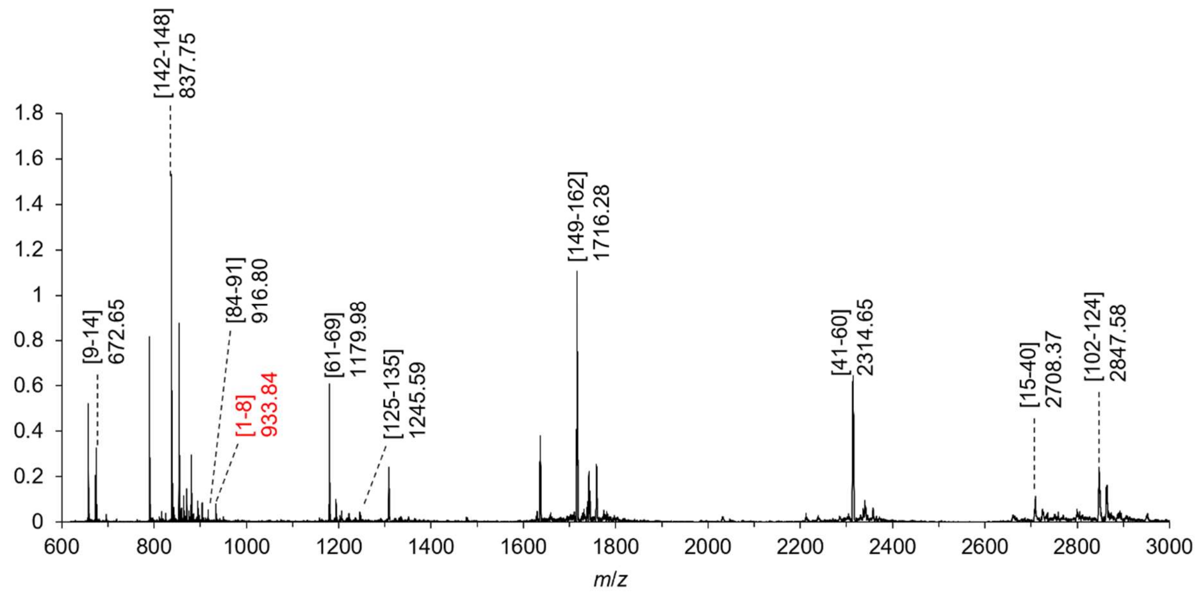

fragment	simulated m/z	charge	sequence
[1-8]	933.5438	1	.LIVTQTMK.g
[9-14]	673.3879	1	k.GLDIQK.v
[15-40]	2707.376	1	k.VAGTWYSLAMAASDISLLDAQSAPLR.v
[41-60]	2313.2588	1	r.VYVEELKPTPEGDLEILLQK.w
[61-69]	1179.4735	1	k.WENDECAQK.k [1xCarbamidomethyl]
[71-75]	573.3606	1	k.IIAEK.t
[78-83]	674.4236	1	k.IPAVFK.i
[84-91]	916.4734	1	k.IDALNENK.v
[92-100]	1065.5827	1	k.VLVLDTDYK.k
[102-124]	2846.298	1	k.YLLFCMENSAEPEQSLVCQCLVR.t [3xCarbamidomethyl]
[125-135]	1245.5845	1	r.TPEVDDEALEK.f
[136-138]	409.2082	1	k.FDK.a
[142-148]	837.4764	1	k.ALPMHIR.l
[149-162]	1715.8057	1	r.LSFNPTQLEEQCHI. [1xCarbamidomethyl]

Figure S33. MALDI-TOF MS spectrum and the list of trypsin-digested peptide fragments of β-lactoglobulin A. Fragments found in MALDI-TOF MS are highlighted in yellow in the list.

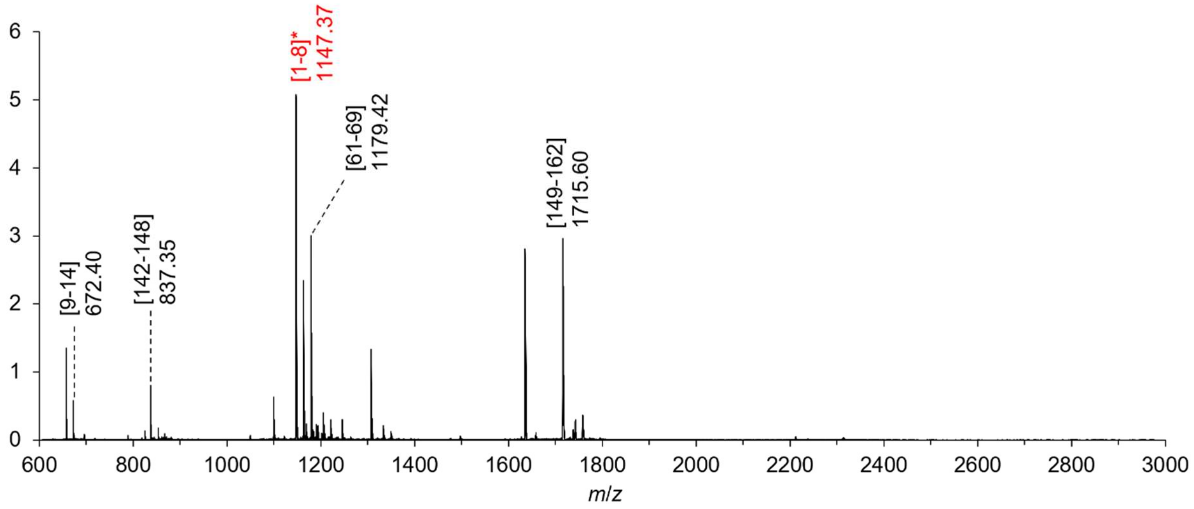

fragment	simulated m/z	charge	sequence
[1-8]	1147.618	1	.LIVTQTMK.g [[1x**2a**_1x**3a**]]
[9-14]	673.3879	1	k.GLDIQK.v
[15-40]	2707.376	1	k.VAGTWYSLAMAASDISLLDAQSAPLR.v
[41-60]	2313.2588	1	r.VYVEELKPTPEGDLEILLQK.w
[61-69]	1179.4735	1	k.WENDECAQK.k [1xCarbamidomethyl]
[71-75]	573.3606	1	k.IIAEK.t
[78-83]	674.4236	1	k.IPAVFK.i
[84-91]	916.4734	1	k.IDALNENK.v
[92-100]	1065.5827	1	k.VLVLDTDYK.k
[102-124]	2846.298	1	k.YLLFCMENSAEPEQSLVCQCLVR.t [3xCarbamidomethyl]
[125-135]	1245.5845	1	r.TPEVDDEALEK.f
[136-138]	409.2082	1	k.FDK.a
[142-148]	837.4764	1	k.ALPMHIR.l
[149-162]	1715.8057	1	r.LSFNPTQLEEQCHI. [1xCarbamidomethyl]

Figure S34. MALDI-TOF MS spectrum and the list of trypsin-digested peptide fragments of the adduct of β-lactoglobulin A with **2a** and **3a**. Fragments found in MALDI-TOF MS are highlighted in yellow in the list.

Inhibitory effect of copper(II) ion, 2a, and acidic pH on aza-Michael addition

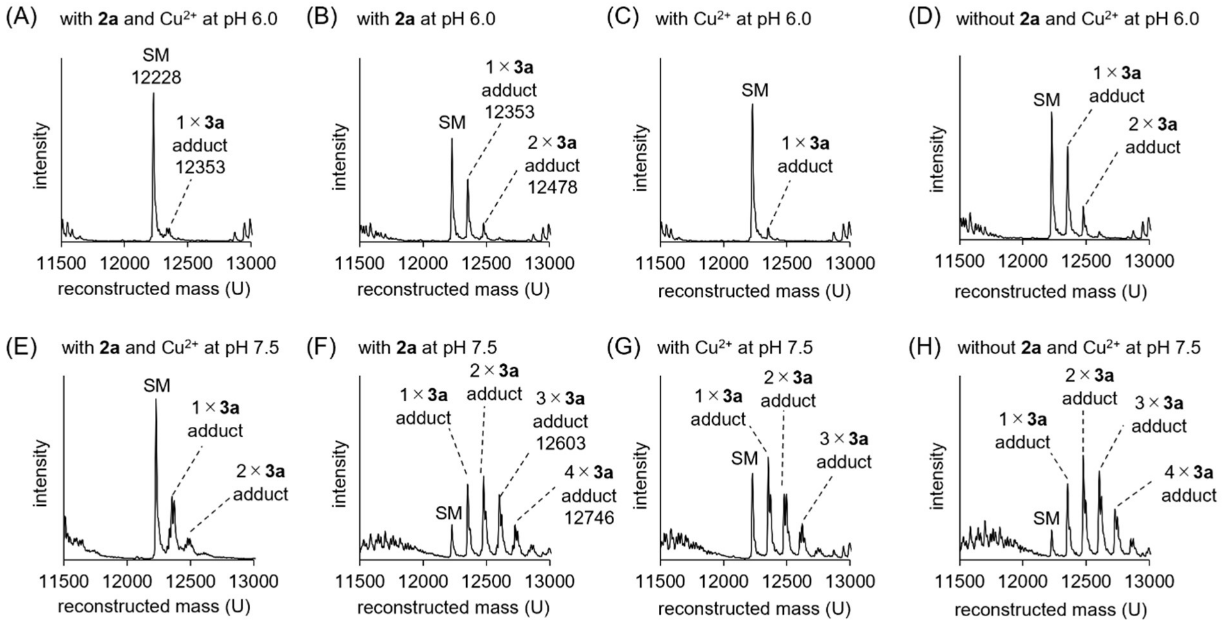

Figure S35. Deconvoluted spectra of the reaction of cytochrome C with **3a** at 37 °C for 18 h. (A) In the presence of **2a** and $Cu(OAc)_2$ at pH 6.0. (B) In the presence of **2a** at pH 6.0. (C) In the presence of $Cu(OAc)_2$ at pH 6.0. (D) In the absence of **2a** and $Cu(OAc)_2$ at pH 6.0. (E) In the presence of **2a** and $Cu(OAc)_2$ at pH 7.5. (F) In the presence of **2a** at pH 7.5. (G) In the presence of $Cu(OAc)_2$ at pH 7.5. (H) In the absence of 2a and $Cu(OAc)_2$ at pH 7.5. Conditions: cytochrome C (1 mg/mL), **3a** (2 mM), $Cu(OAc)_2$ (2 mM) in phosphate buffer (10 mM).

Suppression of Michael addition of cysteine by copper(II) ion, 2a, and acidic pH

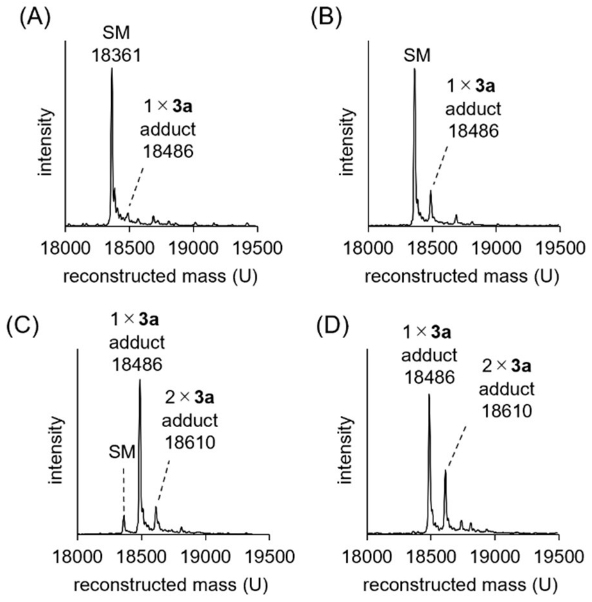

Figure S36. Deconvoluted spectra of the reaction of β-lactoglobulin A with **3a** at 37 °C for 3 h. (A) (B) In the presence and absence of $Cu(OAc)_2$ at pH 6.0. Michael addition of free thiol of Cys121 was suppressed at weakly acidic pH of 6.0. (C) (D) In the presence and absence of $Cu(OAc)_2$ at pH 7.5. Parent protein was almost consumed and the adducts with one or two of **3a** were formed. Reaction conditions: β-lactoglobulin A (36 μM), **3a** (2 mM), $Cu(OAc)_2$ (2 mM) in phosphate buffer (10 mM).

Additional experimental data for sequential modification of lactoglobulin at N-terminus and Cys121

(A)

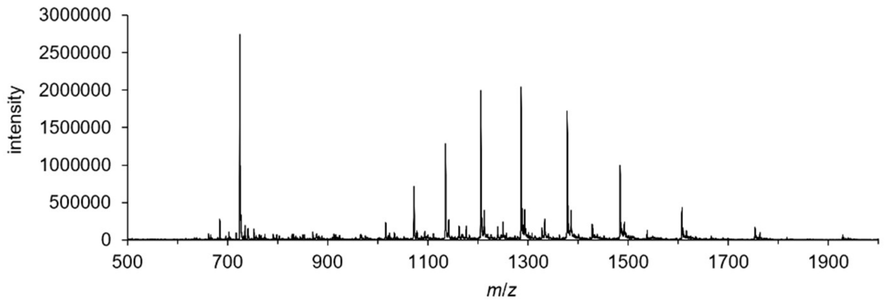

(B)

reconstructed mass (U)	number of peaks used for deconvolution	intensity	standard deviation
19278.4973	9	1043252.33	0.8
19386.885	5	332592.95	1
18576.6942	4	164014.81	0.9
19981.536	4	219500.94	0.6
19320.8756	3	176804.75	1.4
37151.7533	2	151186.5	0.6
38582.8939	2	136288	2.8
11607.7472	2	126770.75	2.3
7430.6983	2	154912.08	0.2
20703.3827	2	176415.5	0.6
37104.3613	2	190795.75	2.7
34503.2159	2	106104.25	1.5
21231.5267	2	192529	0.3
34297.6438	2	180578.5	4.2
7515.5061	2	209305.75	0.4
31848.9415	2	0	1.2
20774.3708	2	233583.25	2.6
27866.4719	2	270986.75	2.5
29725.4593	2	284014	1.7
12927.0754	2	232440.88	2.2
20881.8965	2	302241.88	2.5
17899.3875	2	861161.25	2.7
12296.6859	2	1434439.5	0.8

Figure S37. (A) Mass spectrum of the modified β-lactoglobulin A (**13**) at N-terminus and Cy121, which was used for obtaining the deconvoluted mass spectrum. (B) The list of reconstructed masses calculated using multivalent ions.

Additional experimental data for cross-linked proteins

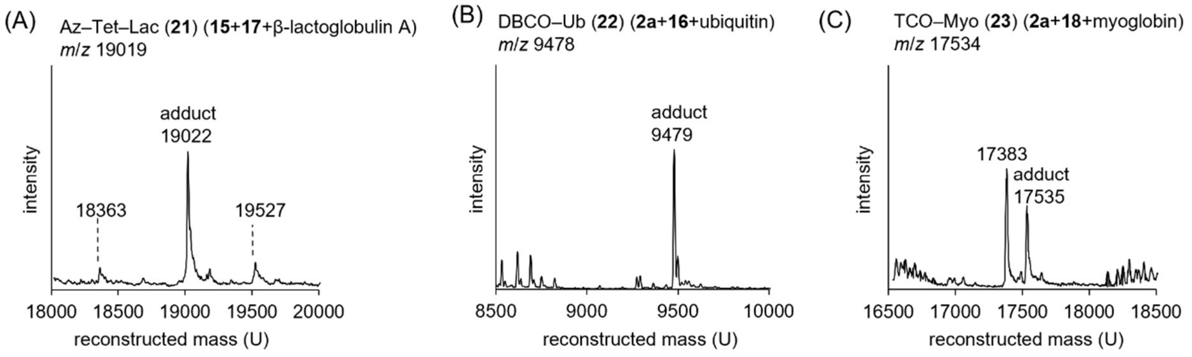

Figure S38. Deconvoluted spectra of (A) azide–tetrazole–β-lactoglobulin A (Az–Tet–Lac, **21**), (B) DBCO–ubiquitin (DBCO–Ub, **22**), and (C) TCO–myoglobin (TCO–Myo, **23**). For **23**, the product resulting from hydrolysis of the carbamate moiety was observed (*m*/*z* 17383).

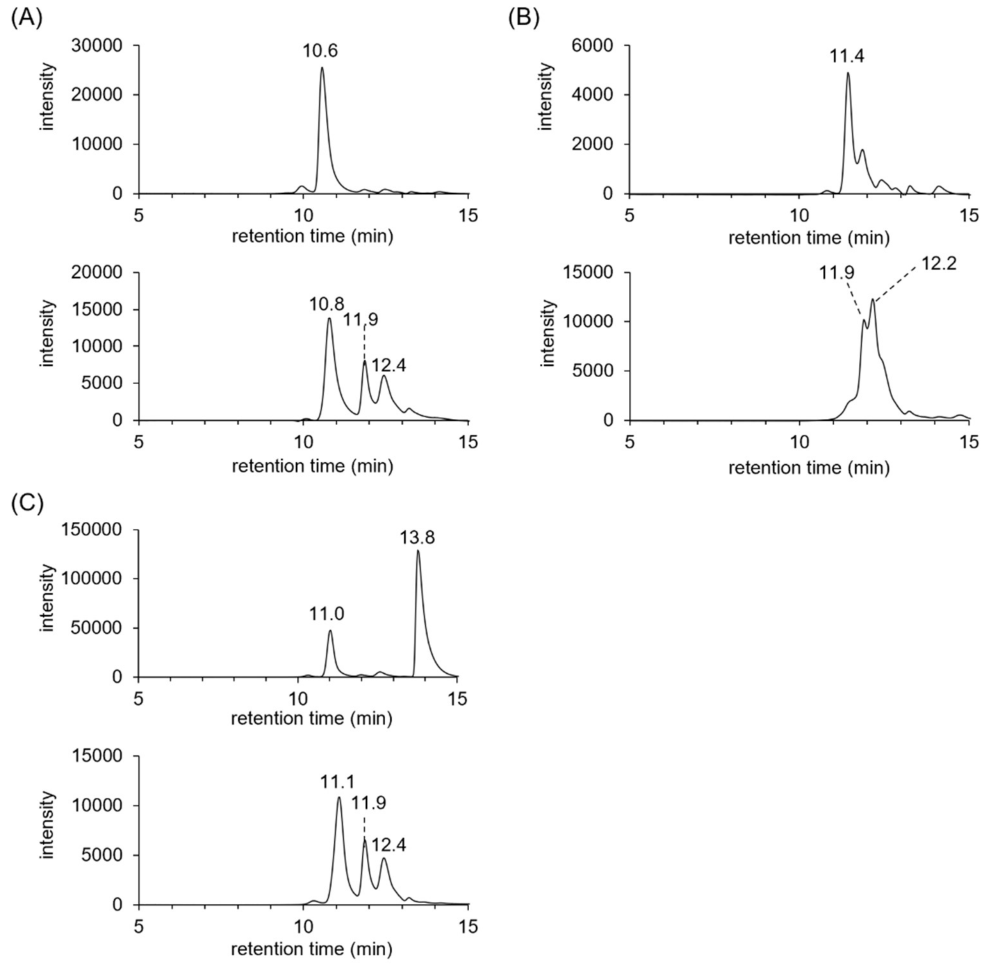

Figure S39. Size-exclusion chromatography analyses; (A) β-lactoglobulin A (top) and azide–tetrazole–β-lactoglobulin A (Az–Tet–Lac, **21**) (bottom); (B) ubiquitin (top) and DBCO–ubiquitin (DBCO–Ub, **22**) (bottom); (C) myoglobin (top) and TCO–myoglobin (TCO–Myo, **23**) (bottom).

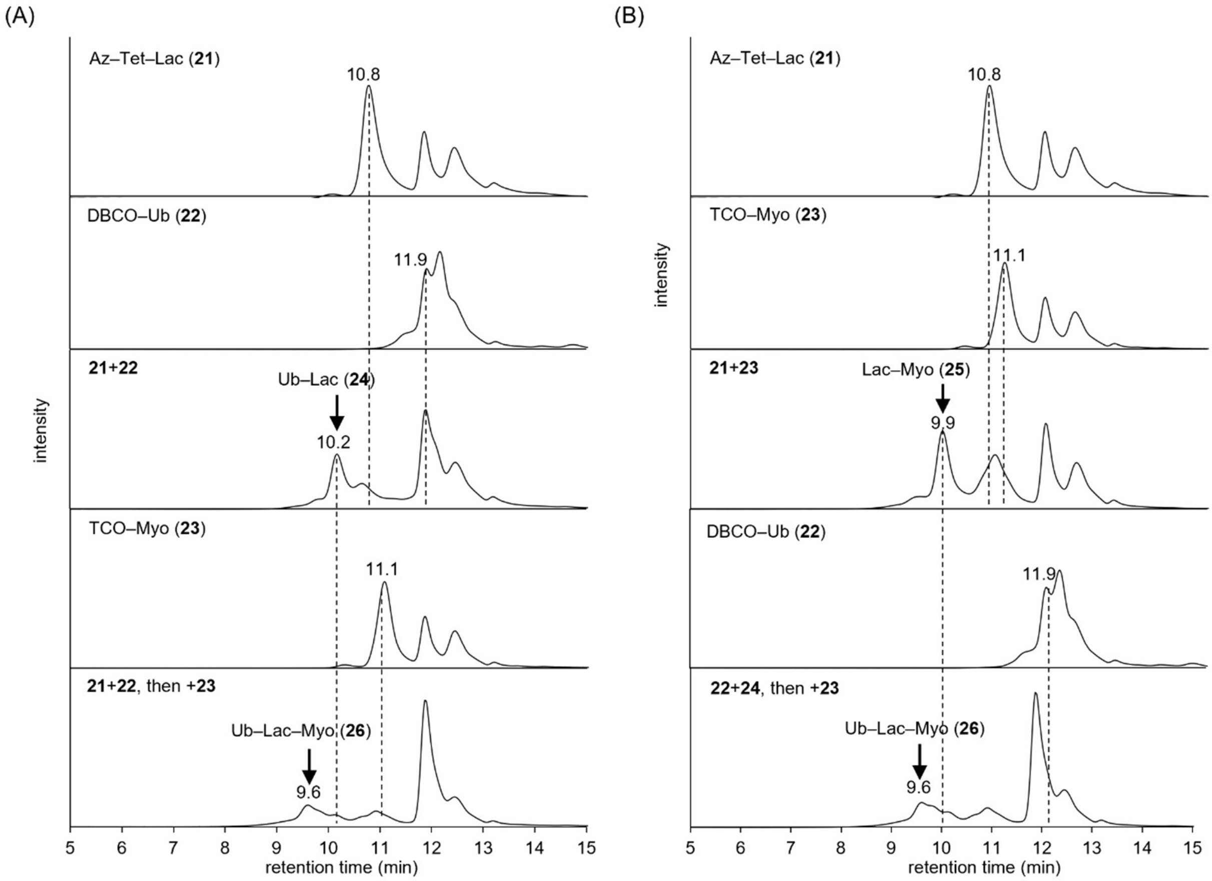

(C)

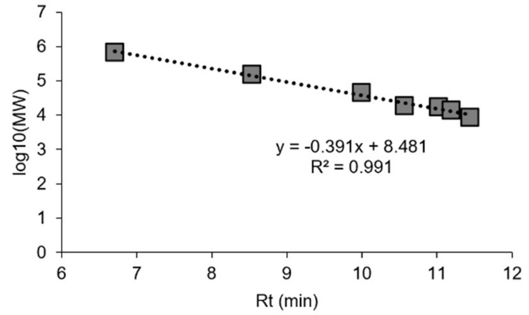

	theoritical MW	calculated MW
Az–Tet–Lac (**21**)	19019	18400
DBCO–Ub (**22**)	9478	10200
TCO–Myo (**23**)	17534	14100
Ub–Lac (**24**)	27012	32000
Lac–Myo (**25**)	36525	41600
Ub–Lac–Myo (**26**)	46031	53000

Figure S40. (A) Size-exclusion chromatography analyses of the reaction mixture of **21** and **22**, and the reaction mixture of **21** and **22**, then **23**. (B) Size-exclusion chromatography analyses of the reaction mixture of **21** and **23**, and the reaction mixture of **21** and **23**, then **22**. (C) The calibration curve for SEC analyses (left) and the list of theoretical and calculated molecular weights of each conjugate (right).

Characterization of MMAE–Cy5–trastuzumab 27

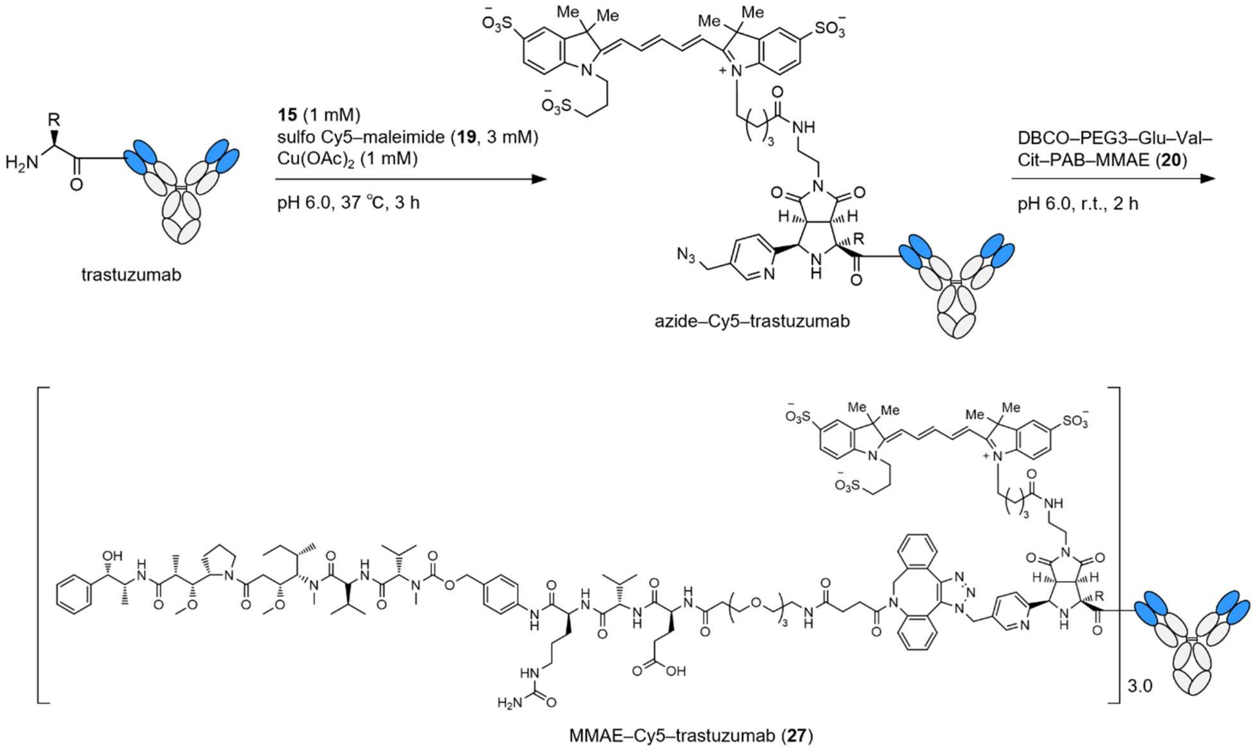

Figure S41. The scheme of the two-step preparation of MMAE–Cy5–trastuzumab via copper(II)-mediated [3+2] cycloaddition with **15** and **19**, followed by ring-strain promoted alkyne-azide cycloaddition reaction with DBCO–PEG3–Glu–Val–Cit–PAB–MMAE (**20**).

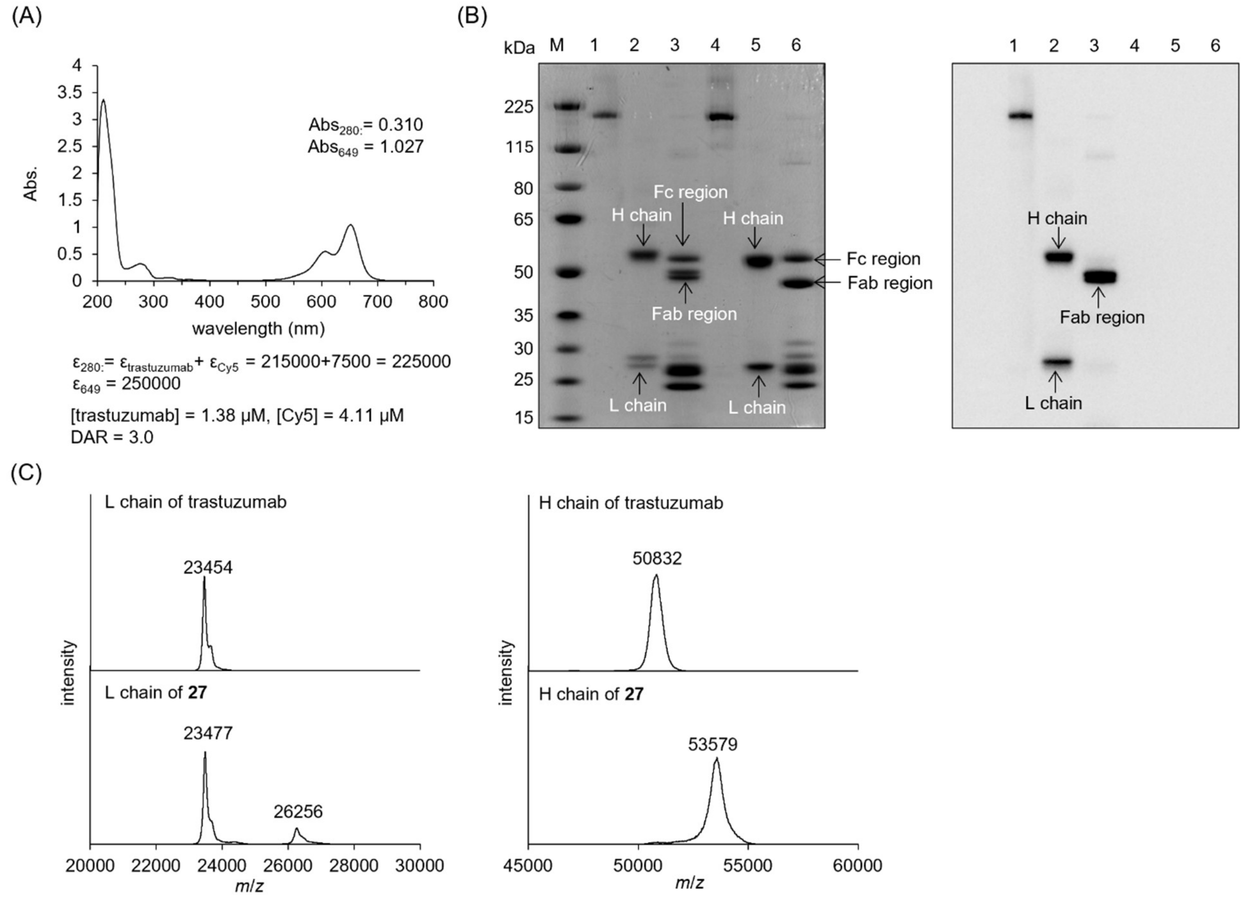

Figure S42. Characterization of MMAE–Cy5–trastuzumab (**27**). (A) UV-Vis spectrum of **27** (l = 1 cm). The drug-to-antibody ratio (DAR) was calculated to be 3.0 by using the molar extinction coefficient (ε) of trastuzumab and Cy5 at 280 nm and 649 nm. (B) CBB gel image (left) and fluorescent gel image (right). Fluorescence imaging was performed by 635-nm excitation with a 670-nm bandpass filter. M: molecular weight marker; lane 1: **27**; lane 2: **27** after reduction of disulfide bonds by treatment with 2-mercaptoethanol; lane 3: **27** after fragmentation by treatment with papain; lane 4: trastuzumab; lane 5: trastuzumab after reduction of disulfide bonds by treatment with 2-mercaptoethanol; lane 6: trastuzumab after fragmentation by treatment with papain. (C) MALDI-TOF MS spectra of trastuzumab and **27** after reduction of disulfide bonds by treatment with TCEP.

(A)

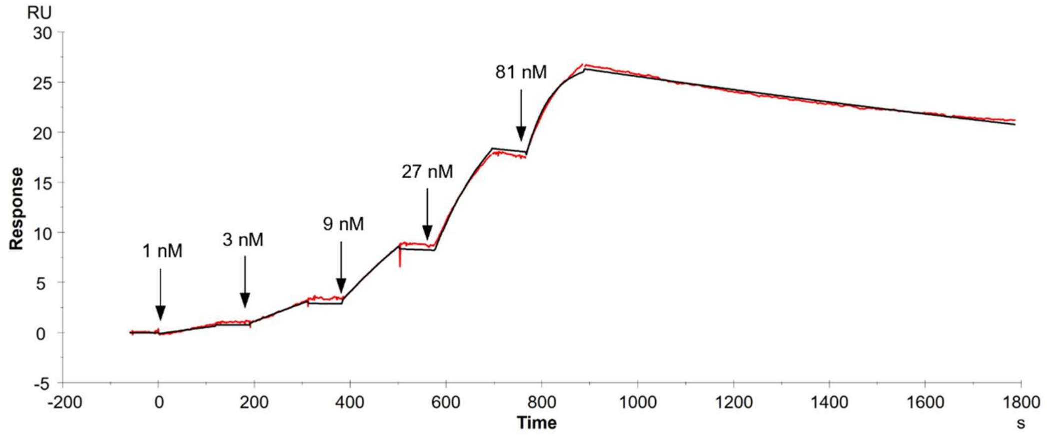

(B)

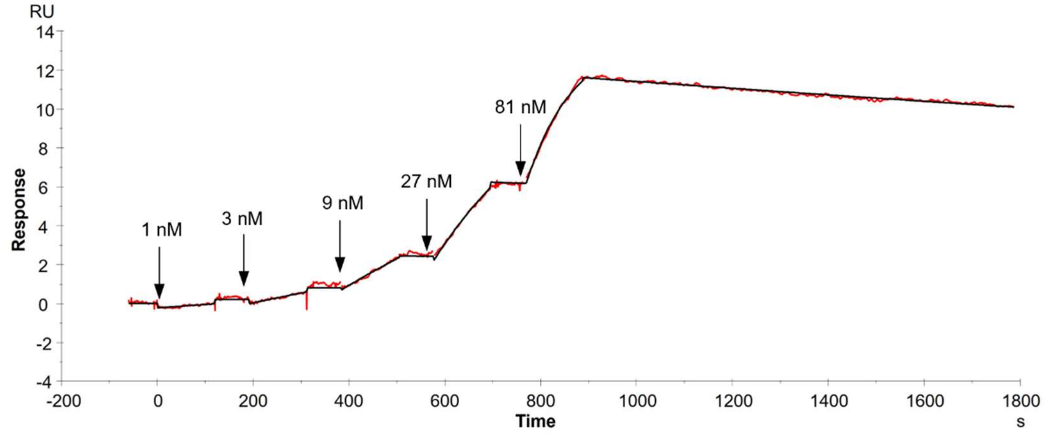

(C)

ligand	amount of immobilized ligand (RU)	k_a (1/M·s)	k_d (1/s)	K_D (M)	theoretical R_{max} (RU)	observed R_{max} (RU)
trastuzumab	43.0	2.4×10^5	2.6×10^{-4}	1.1×10^{-9}	40.3	27.5
**27**	37.9	1.3×10^5	1.6×10^{-4}	1.2×10^{-9}	33.7	14.0

Figure S43. Surface plasmon resonance (SPR) analysis of binding of (A) trastuzumab and (B) MMAE–Cy5–trastuzumab (**27**) to human HER2 / ErbB2 protein. The black and red lines indicate the experimental and simulated binding curves, respectively. (C) The list of the binding parameters.

Additional experimental data for the assessment of anti-proliferative activity of 27

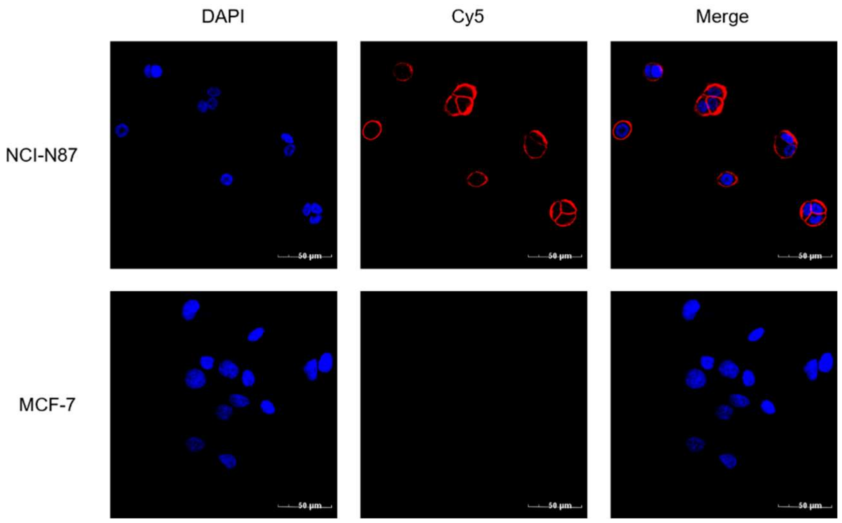

Figure S44. Representative confocal microscopy images of human epidermal growth factor receptor 2 (HER2) positive cells NCI-N87 and HER2 negative cells MCF-7 treated with MMAE–Cy5–trastuzumab **27.**

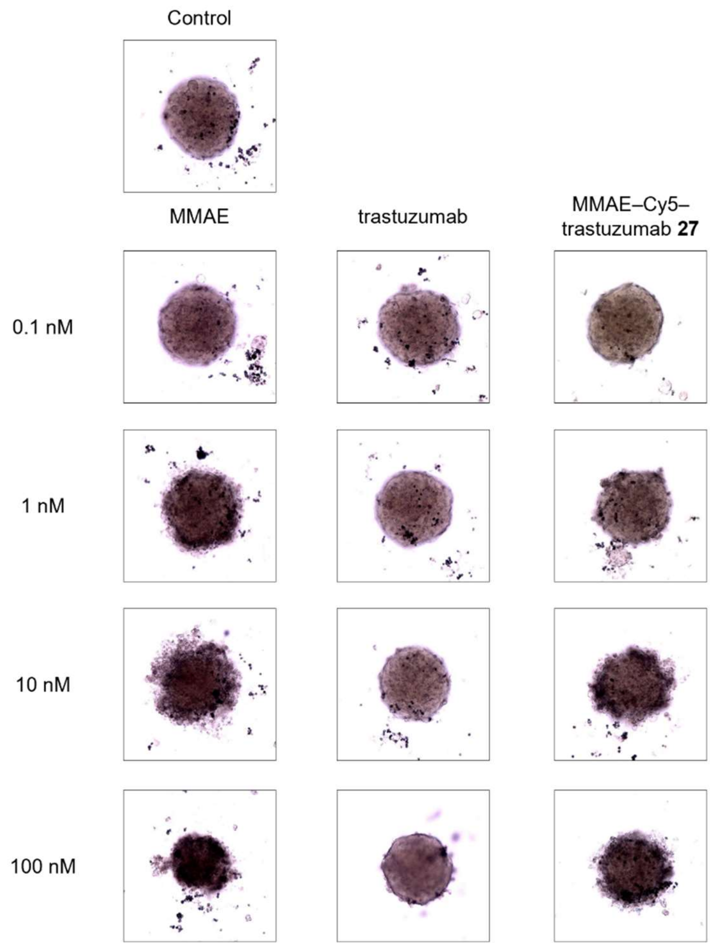

Figure S45. The morphology of NCI-N87 spheroids after treatment with MMAE, trastuzumab, and **27**.

2. Experimental procedures

2-1. General information

All reagents and solvents purchased from commercial suppliers were used without further purification. Angiotensin II (A9525) (**6**), ubiquitin from bovine erythrocytes (U6253), and β-Lactoglobulin A from bovine milk (L7880) were purchased from Sigma-Aldrich. Peptide T (4188-v) (**1**), substance P (**7**) (4173-v), angiotensin IV (**8**) (4331), mastoparan (**9**) (4107-v), delta sleep-inducing peptide (4054-v) (**10**), neurokinin A (**S6**) (4013-v), neuromedin C (**S7**) (4153-v), LH-RH (human) (**S8**) (4013), and α-MSH (**S9**) (4057-v) were purchased from Peptide Institute, INC. Insulin, Human, recombinant expressed in Yeast, animal-free (**12**) (1287886), myoglobin from horse muscle (23550-74), and lysozyme from egg white (19499-91) were purchased from Nacalai tesque. Kisspeptin-10 (**11**) (E2307) was purchased from Selleck Chemicals LLC. Trastuzumab BS was purchased from Daiichi-Sankyo. Biotin–PEG6–maleimide (**14**) (B5563) and TCO–PEG3–maleimide (**18**) (T3948) were purchased from Tokyo Chemical Industry Co., Ltd. Sulfo DBCO–PEG4–maleimide (**16**) (CCT-1231-25) and sulfo Cy5–maleimide (**19**) (FP-1676-25) were purchased from Vector Laboratories, Inc. Methyltetrazine–PEG4–maleimide (**17**) (BP-22436) was purchased from BroadPharm. The concentrations of peptides and proteins (except for myoglobin) solutions were calculated by the absorbance at 280 nm. The molar extinction coefficient at 280 nm of each peptide or protein was computed by Expasy ProtParam tool. The concentration of myoglobin was estimated by Bradford assay using Protein Aassay CBB solution (Nacalai tesque). Silica gel column chromatography was performed using Wakosil® C-300 (spherical and neutral, 40-64 μm, 233-01677, Fujifilm Wako pure chemical Co.) and the Isolera One flash purification system (Biotage). ^{1}H and ^{13}C NMR spectra were measured on a Bruker ADVANCE 500 or ADVANCE NEO 600 spectrometer (Bruker). The chemical shifts were calibrated to the residual solvent peaks ($CDCl_3$: 77.16 ppm for ^{13}C NMR, d_6-DMSO: 2.50 ppm for ^{1}H NMR and 39.52 ppm for ^{13}C NMR) or TMS (0.00 ppm for ^{1}H NMR). Electrospray ionization mass spectrometry (ESI-MS) was recorded on JMS-T100LP AccuTOF (JASCO Co.). High-performance liquid chromatography (HPLC) was performed with CBM-20A, SPD-10A VP, LC-20AD, and CTO-10AS VP (Shimadzu) using COSMOSIL 5C18-AR-II (Φ 4.6 x 150 mm, Nacalai tesque) for analytical scale and COSMOSIL 5C-18 MS-II (Φ 10 x 250 mm, Osaka Soda) for preparative scale. The flow rates were 1 mL/min for analytical scale and 4 mL/min for preparative scale. In every HPLC analysis, a gradient of 0.1% trifluoroacetic acid (TFA) in H_2O (mobile phase A) and 0.1% TFA in acetonitrile (mobile phase B) was employed. Compounds were detected by UV detector at 230 nm or 280 nm. Size exclusion chromatography was performed with SCL-40, DGU-403, photo diode array detector SPD-M40, LC-40D XR, and CTO-40S (Shimadzu) using COSMOSIL 5Diol-300-II (Φ 7.5 x 300 mm, Nacalai tesque). The flow rates were 1 mL/min and the column oven temperature was maintained at 30 °C. Arg-SEC mobile phase (standard) (Nacalai tesque) was used as a mobile phase. MALDI-TOF MS analysis was performed on MALDI-8020 (Shimadzu). Each reaction mixture was desalted using ZipTip® C4 or C18 (Merck) in accordance with supplier's protocol prior to MALDI-TOF MS analysis. α-Cyano-4-hydroxycinnamic acid (CHCA) (20 mg/mL solution in 50:50:0.1 H_2O/MeCN/TFA) or 3,5-dimethoxy-4-hydroxycinnamic acid (SA) (20 mg/mL solution in 50:50:0.1 H_2O/MeCN/TFA) was used as a matrix in MALDI-TOF MS analysis. LC-MS and LC-MS/MS analyses were conducted on CBM-20A, SPD-20A, LC-20AD, CTO-20A, SIL-20AC HT, and LCMS-8050 (Shimadzu) with COSMOSIL 5C18-MS-II (Φ 2.0 x 150 mm, Nacalai tesque) for peptides. A gradient of 0.1% formic acid (FA) in

H_2O (mobile phase A) and 0.1% FA in acetonitrile (mobile phase B) was employed. Deconvoluted mass spectra of modified proteins were obtained using LabSolutions Insight Explore software (Shimadzu). Electrophoresis was conducted by using NuPAGE™ 4–12% or 12% Bis-Tris Protein Gels (Invitrogen) and XCell SureLock™ Mini-Cell (Invitrogen) in accordance with supplier's protocol. Gel images were recorded on Amersham ImageQuant800 (Cytiva). ICP-OES analysis was performed on Agilent 5100 VDV system (Agilent). Surface plasmon resonance (SPR) analysis was performed with Biacore T200 (Cytiva) and Series S Sensor Chip CM5 (29104988, Cytiva) using HBS-EP+ buffer 10× (BR-1008-26, Cytiva), Human antibody capture kit (BR-1008-39, Cytiva), and Human Her2 / ErbB2 Protein, His Tag (HE2-H5225, AcroBiosystems). SPR data was analyzed using Biacore T200 Evaluation Software ver.2.0 (Cytiva).

2-2. Peptide modification

General procedure for copper(II)-mediated N-terminal modification of peptide

Peptide (2 μL, 2.5 mM in H_2O) was incubated with an aldehyde (1 μL, 50 mM in DMSO), a dipolarophile (1 μL, 50 mM in DMSO), and $Cu(OAc)_2$ (1 μL, 50 mM in H_2O) in phosphate buffer (20 μL, 10 mM, pH 6.0) for a given time of period at 37 °C. The reaction was quenched with EDTA (1 μL, 100 mM in H_2O) and methoxyamine (1 μL, 1 M in H_2O at pH 4.5). The mixture was analyzed by LC-MS (gradient elution: 0–3 min, 5% B; 3–16 min, 5–75% B), LC-MS/MS (gradient elution: 0–3 min, 5% B; 3–19 min, 5–95% B), and MALDI-TOF MS.

Preparation of the modified peptide T 4aa for the structure determination by NMR studies

Peptide T (**1**) (1 mL, 2.5 mM in H_2O) was incubated with **2a** (2.4 μL), **3a** (125 μL, 200 mM in DMSO), and $Cu(OAc)_2$ (250 μL, 100 mM in H_2O) in phosphate buffer (11.125 mL, 10 mM, pH 6.0) for 3 h at 37 °C. The reaction was quenched with EDTA (0.5 mL, 100 mM in H_2O). The resulting solution was purified by preparative HPLC (gradient elution: 0–15 min, 15% B. The fractions at 8.5 and 10.2 min were collected and lyophilized to obtain a colorless powder. The powder was dissolved in DMSO-d_6 (600 μL) and analyzed by LC-MS, ^{1}H NMR, ^{13}C NMR, HSQC, HMBC, and TOCSY.

X-ray crystallographic analysis of (±)-5, N-terminal substructure of 4aa

The analysis of (±)-**5** was performed on a diffractometer equipped with a beamline BL-5A at KEK (the High Energy Accelerator Research Organization, Japan) with a Pilatus3 S6M detector (synchrotron, λ = 0.7500 Å, T = 95 K). Using Olex2,[1] the structure was solved with the SHELXT[2] structure solution program using Intrinsic Phasing and refined with the SHELXL[3] refinement package using Least Squares minimization. All non-hydrogen atoms were refined with anisotropic displacement parameters. All hydrogen atoms were created with ideal geometry and refined using a riding model.

Crystallographic data have been deposited with Cambridge Crystallographic Data Centre: Deposition number CCDC-2354963. Copies of the data can be obtained free of charge via http://www.ccdc.cam.ac.uk/conts/retrieving.html (or from the Cambridge Crystallographic Data Centre, 12, Union Road, Cambridge, CB2 1EZ, UK; Fax: +44 1223 336033; e-mail: deposit@ccdc.cam.ac.uk).

The single crystal of (±)-**5** was prepared by a vapor diffusion method. (±)-**5** was dissolved in AcOEt. The solution in a micro vial was put into a vial filled with pentane and stayed overnight. Crystal data of (±)-**5**: $C_{32}H_{40}N_8O_6$, colorless, 0.2 × 0.15 × 0.07 mm^3, orthorhombic, space group $Pna2_1$, a = 19.2530(6), b = 17.0120(6), c = 9.706(2) Å, α = 90°, β = 90°, γ = 90°, V = 3179.0(7) Å^3 , ρ_{calcd} = 1.322 g/cm^3 , Z = 4, 11265 unique reflections (R_{int} = 0.0210, R_{sigma} = 0.0136) out of 68265 with I > 2σ (I) reflections measured, R_1 = 0.03883, and wR_2 = 0.1050 [I > 2σ(I)], R_1 = 0.0403, and wR_2 = 0.1068 for all data, GOF = 1.060, and flack parameter = −0.04(9).

Kinetic study of the modification of peptide T (1) with 2a and 3a

Peptide (10 µL, 2.5 mM in H_2O) was incubated with **2a** (5 µL, 50 mM in DMSO), **3a** (5 µL, 50 mM in DMSO), and $Cu(OAc)_2$ (5 µL, 50 mM in H_2O) in phosphate buffer (100 µL, 10 mM, pH 6.0) for a given time of period at 37 °C. An aliquot (12.5 µL) was taken and was quenched with EDTA (0.5 µL, 100 mM in H_2O) and methoxyamine (0.5 µL, 1 M in H_2O at pH 4.5). The mixture was analyzed by LC-MS (gradient elution: 0–3 min, 5% B; 3–16 min, 5–75% B), LC-MS/MS (gradient elution: 0–3 min, 5% B; 3–19 min, 5–95% B).

Stability test of the modified peptide T 4aa

The modified peptide T **4aa** (10.9 µL, 1.83 mM in DMSO-d_6) was incubated for a given period of time in the following solution (89.1 µL): aqueous HCl solution (0.1 M), phosphate buffer (50 mM, pH 7.4), aqueous NaOH solution (0.1 M), aqueous glutathione (reductive form) solution (1 mM in phosphate buffer (50 mM, pH 7.4)), aqueous TCEP solution (1 mM in phosphate buffer (50 mM, pH 7.4)), or aqueous H_2O_2 solution (1 mM in phosphate buffer (50 mM, pH 7.4)). The reaction mixture was analyzed by LC-MS. The remaining percent of **4aa** was calculated by using the peak area in total ion chromatograms.

2-3. Protein modification

***N*-Terminal protein modification with 2a and 3a**

A solution (2 µL) of myoglobin (10 mg/mL in 10 mM phosphate buffer at pH 7.5), lysozyme (10 mg/mL, 10 mM phosphate buffer at pH 7.5), ubiquitin (10 mg/mL in 10 mM phosphate buffer at pH 7.5), or β-lactoglobulin A (10 mg/mL, 10 mM phosphate buffer at pH 7.5) was incubated with **2a** (1 µL, 50 mM in DMSO), **3a** (1 µL, 50 mM in DMSO) and $Cu(OAc)_2$ (1 µL, 50 mM in H_2O) in phosphate buffer (20 µL, 10 mM, pH 6.0) for a given period of time at 37 °C. The reaction was quenched with EDTA (1 µL, 100 mM in H_2O) and methoxyamine (1 µL, 1 M in H_2O at pH 4.5). The mixture was analyzed by LC-MS. Deconvoluted mass spectrum was obtained using LabSolutions Insight Explore software.

In-gel digestion and MALDI-TOF MS and LC-MS/MS analysis for identification of modification site

The reaction mixture of myoglobin, lysozyme, ubiquitin, or β-lactoglobulin A with **2a** and **3a** was diluted with 2×sample buffer with 2-mercaptoethanol (30566-22, nacalai tesque, INC.) and the resulting

mixture was boiled for 5 min. The samples were loaded on a 12% acrylamide gel and run for 50 min at 200 V constant. After CBB staining, each band corresponding to modified proteins was separated. Separated gels were cut into ca. 1-mm cubes. The gel pieces were washed twice with 50% MeCN, 0.1% TFA in H_2O. For destaining, the gel pieces were incubated with 50% MeCN in aqueous NH_4HCO_3 solution (100 mM) for 45 min at 37 °C for de-staining and then in MeCN at room temperature for dehydration. To the tube, 100 mM dithiothreitol in aqueous NH_4HCO_3 solution (100 mM) was added to reduce disulfide bonds in a protein. The resulting mixture was reacted at 37 °C for 30 min. Freed thiols were capped by incubation with 250 mM iodoacetamide in aqueous NH_4HCO_3 solution (100 mM) for 30 min at room temperature under dark. The gels were washed sequentially with aqueous NH_4HCO_3 solution (100 mM), 50% MeCN in aqueous NH_4HCO_3 solution (100 mM), and MeCN. After drying gels, trypsin gold (Promega) (20 µg/mL in 10% MeCN in aqueous NH_4HCO_3 solution (40 mM)) was added to dried gels in the tube and the mixture was incubated for 60 min at rt. To the mixture, 10% MeCN in in aqueous NH_4HCO_3 was further added and the whole was incubated overnight at 37 °C. The supernatant was collected in a new tube and the gels were washed with H_2O and 50% MeCN, 5% TFA in H_2O. Liquids were combined and freeze-dried. The residue was redissolved in 0.1% TFA in H_2O and desalted and concentrated using ZipTip® C18 to prepare a sample for MALDI-TOF MS.

N-Terminal protein modification with 2-PCA derivative and maleimide derivative

A solution (32 µL) of myoglobin (10 mg/mL in 10 mM phosphate buffer at pH 7.5), ubiquitin (10 mg/mL in 10 mM phosphate buffer at pH 7.5), or β-lactoglobulin A (10 mg/mL, 10 mM phosphate buffer at pH 7.5) was incubated with 2-PCA **2a** or **15** (16 µL, 50 mM in DMSO), maleimide **17**—**19** (16 µL, 50 mM in DMSO) and $Cu(OAc)_2$ (16 µL, 50 mM in H_2O) in phosphate buffer (320 µL, 10 mM, pH 6.0) for 3 h at 37 °C. The reaction was quenched with EDTA (16 µL, 100 mM in H_2O) and methoxyamine (16 µL, 1 M in H_2O at pH 4.5). The mixture was desalted with Zeba™ spin desalting column 7K MWCO and the filtrate was dialyzed in phosphate buffer (20 mM, pH 7.0) using Slide-A-Lyzer Dialysis Cassette 3.5K MWCO (Thermo Fisher) overnight in a cold room. The adducts were analyzed by LC-MS. Deconvoluted mass spectrum was obtained using LabSolutions Insight Explore software. The concentration of each adduct was estimated by Bradford protein assay for **23** and UV-Vis spectroscopy for **21** and **22**. The following values were used as molar extinction coefficients (ε) at 280 nm. ubiquitin: 1490; β-lactoglobulin A: 17200; methyltetrazine: 12800. The concentrations of **21**, **22**, and **23** were calculated to be 27.5 µM, 29.5 µM, and 16.4 µM, respectively.

Stepwise preparation of ternary conjugate of N-terminal functionalized proteins

Method A: **21**+**22**, then +**23**

Az–Tet–Lac (**21**) (30 µL, 27.5 µM in 20 mM phosphate buffer at pH 7.0) and DBCO–ubiquitin (**22**) (30 µL, 29.5 µM in 20 mM phosphate buffer at pH 7.0) were incubated for 24 h at room temperature. An aliquot (30 µL) was taken for analysis. To the remaining reaction mixture, TCO–Myo (**23**) (15 µL, 16.4 µM in 20 mM phosphate buffer at pH 7.0) was added, and the mixture was incubated for 15 h at room temperature. The resulting mixture was analyzed by SDS-PAGE and HPLC with a size exclusion column.

Method B: **21**+**23**, then +**22**

Az–Tet–Lac (**21**) (30 μL, 27.5 μM in 20 mM phosphate buffer at pH 7.0) and TCO–Myo (**23**) (30 μL, 16.4 μM in 20 mM phosphate buffer at pH 7.0) were incubated for 15 h at room temperature. An aliquot (30 μL) was taken for analysis. To the remaining reaction mixture, DBCO–ubiquitin (**22**) (15 μL, 29.5 μM in 20 mM phosphate buffer at pH 7.0 was added, and the mixture was incubated for 24 h at room temperature. The resulting mixture was analyzed by SDS-PAGE and HPLC with a size exclusion column.

Preparation of MMAE–Cy5–trastuzumab (27)

Trastuzumab (48 μL, 115 μM in PBS) was incubated with azide–2-PC **15** (12 μL, 50 mM in DMSO), $Cu(OAc)_2$ (12 μL, 50 mM in H_2O), and sulfo Cy5–maleimide **19** (36 μL, 50 mM in DMSO) in phosphate buffer (492 μL, 10 mM, pH 6.0) for 3 h at 37 °C. The reaction was quenched with EDTA (24 μL, 100 mM in H_2O) and methoxyamine (24 μL, 1 M in H_2O at pH 4.5). The mixture was desalted with Zeba™ spin desalting column 40K MWCO (Thermo Fisher) to afford azide–Cy5–trastuzumab. DBCO–PEG3–Glu–Val–Cit–PMB–MMAE **20** (15 μL, 5 mM in DMSO) was added to the filtrate and the mixture was incubated for 2 h at r.t. The mixture was desalted with Zeba™ spin desalting column 40K MWCO and the filtrate was dialyzed in PBS using Slide-A-Lyzer Dialysis Cassette 10K MWCO (Thermo Fisher) overnight in a cold room. The conjugation of sulfo Cy5 to trastuzumab was confirmed by MALDI-TOF MS after the treatment with TCEP and SDS-PAGE. Drug-to-antibody ratio (DAR) was calculated to be 3.0 by UV-Vis spectroscopy. For UV-Vis spectroscopy, the following values were used as molar extinction coefficients (ε) of trastuzumab and sulfo Cy5 at 280 nm and 366 nm. Trastuzumab: ε_{280} = 215000; sulfo Cy5: ε_{280} = 7500, ε_{649} = 250000.

Papain digestion of MMAE–Cy5–trastuzumab (27)

Papain (10 μL, 10 mg/mL in phosphate buffer at pH 6.5) was diluted with activation buffer (90 μL, 10 mM EDTA, 10 mM cysteine in PBS). The mixture was incubated for 10 min at 37 °C. A solution of activated papain (10 μL) was added to MMAE–Cy5–trastuzumab (25 μL, 1.38 μM in PBS). The mixture was incubated for 1 h at 37 °C and quenched with aqueous H_2O_2 solution (1 μL, 100 mM). The resulting mixture was incubated for 1 h at 37 °C. For SDS-PAGE, the mixture was diluted with 6×sample buffer (10 μL, 09499-14, nacalai tesque, INC.) and the resulting mixture was boiled for 5 min. The samples (8 μL) were loaded on a 4–12% acrylamide gel and run for 45 min at 200 V constant. After a fluorescence image was obtained, the same gel was stained with CBB stain one (04543-51, Nacalai tesque) in accordance with the supplier's protocol.

Surface plasmon resonance (SPR) analyses for determination of the binding affinity of trastuzumab and 27 with HER2

Anti-Human IgG(Fc) antibody was immobilized on Series S Sensor Chip CM5 (Cytiva) using Human antibody capture kit (Cytiva), following the supplier's protocol. Trastuzumab and MMAE–Cy5–

trastuzumab (**27**) were diluted to 0.1 and 0.2 μg/mL, respectively, in HBS-EP+ buffer (Cytiva), the running buffer. Each sample was injected over the anti-human IgG(Fc) antibody-immobilized sensor surface at a flow rate of 10 μL/min for 60 sec at 25 °C. The resonance units (RU) of captured trastuzumab and **27** captured on the flow cells were approximately 42.0 RU and 37.6 RU, respectively. Human Her2/ErbB2 Protein (AcroBiosystems) (1, 3, 9, 27, and 81 nM in HBS-EP+ buffer) was injected over the surfaces at 30 μL/min and 25 °C for 120 sec. The running buffer was maintained at a flow rate of 30 μL/min and 25 °C during 900 sec dissociation phase. For the binding of trastuzumab and **27** to HER2, the binding kinetics, k_{on} (M^{-1} s^{-1}), k_{off} (s^{-1}), and K_D (M) were calculated from global fittings using a 1:1 kinetics binding model with the Biacore T200 evaluation software ver 2.0.

Cell culture

NCI-N87 cells, a human gastric adenocarcinoma cell line, and MCF-7 cells, a human breast cancer cell line, were maintained in RPMI-1640 medium and McCoy's 5a medium, respectively. Both media were supplemented with 10% fetal bovine serum, 100 U/mL of penicillin, and 100 mg/mL of streptomycin. The cells were incubated in an atmosphere of 5% CO_2 at 37°C.

Immunofluorescence experiment

NCI-N87 cells and MCF-7 cells were seeded in m-Dish (φ = 35 mm; ibidi, Germany) at a cell density of 10,000 cells/dish and cultured overnight. After removing the medium, the cells were washed with PBS and fixed with 4% paraformaldehyde for 10 min at room temperature and pH 7.4. After washing with PBS three times, the cells were blocked in 3% bovine serum albumin solution (in PBS) at room temperature for 30 min. The blocking solution was removed, and the cells were incubated with 100 nM MMAE–Cy5–trastuzumab and 1% DAPI in 3% bovine serum albumin solution at room temperature for 1 hour. The cells were washed with PBS three times, and then fluorescence images were obtained using an FV1000 confocal laser scanning biological microscope (Olympus, Japan). ImageJ software was used to generate images suitable for publication.

Cytotoxicity assay in 2D- and 3D-cultured model

The NCI-N87 (HER2 positive, 25,000 cells/well) and MCF-7 cells (HER2 negative, 3,000 cells/well) were seeded in a 96-well cell culture plate overnight. Thereafter, the cells were treated with either MMAE (0, 0.001, 0.01, 0.1, 1, 2.5, 5, 10, 50, and 100 nM), trastuzumab (0, 0.00035, 0.0035, 0.035, 0.35, 0.875, 1.75, 3.5, 17.5, and 35 nM), or **27** (MMAE [trastuzumab]: 0 [0], 0.001 [0.00035], 0.01 [0.0035], 0.1 [0.035], 1 [0.35], 2.5 [0.875], 5 [1.75], 10 [3.5], 50 [17.5], and 100 [35] nM) for 72 h. Cell viability was determined using a cell count reagent (nacalai tesque, Japan).

To prepare NCI-N87 spheroids, NCI-N87 cells (2.5×10^3 cells/well) were prepared by a liquid overlay method using an ultralow attachment 96-well cell culture plate at 37°C for 96 h.[4] Thereafter, either MMAE (0, 0.1, 1, 10, and 100 nM), trastuzumab (0, 0.035, 0.35, 3.5, and 35 nM), or **27** (MMAE [trastuzumab]: 0 [0], 0.1 [0.035], 1 [0.35], 10 [3.5], and 100 [35] nM) was added to spheroids (final

concentration: 0, 0.1 1, 10, and 100 nM), and further incubated at 37 °C for 72 h. The image of spheroid morphology using a phase-contrast microscope (BZ-X700).

Confocal microscopy

NCI-N87 cells (10,000 cells/dish) and MCF-7 cells (10,000 cells/dish) were cultured in μ-Dish (ibidi, Germany) overnight. After washing with PBS thrice, cells were fixed with 10% paraformaldehyde at room temperature for 10 min. The cells were washed with PBS thrice and blocked in PBS containing 3% bovine serum albumin at room temperature for 30 min. The blocking solution was removed, and the cells were incubated with 100 nM MMAE–Cy5–trastuzumab **27** with 1% DAPI in 3% bovine serum albumin solution at room temperature for 1 h. The cells were washed with PBS thrice, then fluorescence images were obtained by FV3000 confocal laser scanning biological microscope (Olympus, Japan).

Biodistribution experiment in NCI-N87 tumor-bearing mice

After acclimation for one-week, five BALB/c Slc-nu/nu mice (7-week-old females, Japan SLC, Inc., Shizuoka, Japan) were subcutaneously inoculated NCI-N87 cells (1.5×10^7 cells/mouse) on the back of the right femoral area. Four NCI-N87 tumor-bearing mice (tumor size: 169 ± 42 mm3) were intravenously received MMAE–Cy5–trastuzumab **27** (0.22 mg/kg, 7.5 mL/kg). At 1, 3, 6, 12, and 24 h after injection, the fluorescence intensity of whole body was obtained using IVIS Lumina LT (PerkinElmer Inc., Waltham, MA, USA). After obtaining final whole-body image (24 h), the mice were euthanized by cervical dislocation to harvest biological samples (blood, kidneys, liver, spleen, heart, lungs, and tumor). Fluorescence intensity (Ex/Em = 660/710 nm) in the biological samples were quantitative by IVIS Lumina LT (PerkinElmer Inc., Waltham, MA, USA). All experiments related to animals were carried out according to ARRIVE guidelines. The animal experiments were approved by the Institutional Animal Care and Use Committee of Keio University (Ethics accreditation number: A2022-123).

2-4. Organic syntheses

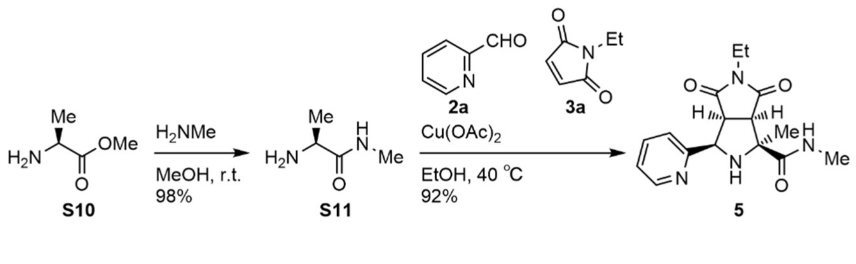

2-Amino-*N*-methylpropanamide (S11)

L-Alanine methyl ester hydrochloride (**S10**) (5.33 g, 38.2 mmol) was added to a 40% solution of methylamine in MeOH. The solution was stirred for 24 h at room temperature. The mixture was concentrated *in vacuo*. The residue was suspended with aqueous NaOH solution saturated with NaCl (10 M, 15 mL). The organic materials were extracted with $CHCl_3$ (5 × 20 mL). The combined organic layers were dried over Na_2SO_4, filtered, and concentrated *in vacuo* to afford **S11** (3.83 g, 98% yield) as colorless oil. ^{1}H NMR (600 MHz, $CDCl_3$/TMS): δ 3.50 (q, *J* = 7.0 Hz, 1H), 2.82 (d, *J* = 5.1 Hz, 3H),

1.34 (d, J = 7.0 Hz, 3H). ^{13}C NMR (125 MHz, $CDCl_3$): δ 176.4, 50.8, 25.9, 21.9. These data were in good agreement with those reported in ref 5.[5]

(1S*,3R*,3aS*,6aR*)-5-Ethyl-*N*,1-dimethyl-4,6-dioxo-3-(pyridin-2-yl)octahydropyrrolo[3,4-c]pyrrole-1-carboxamide (5)

2-Pyridinecarboxaldehyde (**2a**, 107 mg, 1.00 mmol), N-ethylmaleimide (**3a**, 125 mg, 1.00 mmmol) and $Cu(OAc)_2$ (202 mg, 1.01 mmol) were added to a solution of **S11** (101 mg, 0.992 mmol) in EtOH (15 mL). The suspension was stirred for 18 h at 40 °C. Aqueous ethylenediamine-*N,N,N',N'*-tetraacetic acid, tetrasodium salt, dihydrate (EDTA·4Na) solution (100 mM, pH 7, 40 mL) was added to the mixture. The organic materials were extracted with $CHCl_3$ (3 × 30 mL). The combined organic layers were washed with brine (20 mL), dried over Na_2SO_4, filtered, and concentrated *in vacuo*. The residue was purified using Isolera One system ($CHCl_3$/MeOH, 2–18%) to afford **5** (289 mg, 92% yield) as a colorless amorphous solid. ^{1}H NMR (600 MHz, $CDCl_3$/TMS): δ 8.60 (dd, J = 1.7, 4.8 Hz, 1H), 7.70 (ddd, J = 1.7, 7.5, 7.8 Hz, 1H), 7.37 (dd, J = 0.8, 7.8 Hz, 1H), 7.26 (ddd, J = 0.8, 4.8, 7.5 Hz, 1H), 7.11 (br, 1H), 4.92 (d, J = 8.7 Hz, 1H), 3.63 (dd, J = 7.9, 8.7 Hz, 1H), 3.40 (q, J = 7.2 Hz, 2H), 3.22 (d, J = 7.9 Hz, 1H), 2.86 (d, J = 4.9 Hz, 3H), 1.67 (s, 3H), 1.05 (t, J = 7.2 Hz, 3H). ^{13}C NMR (151 MHz, $CDCl_3$): δ 174.7, 174.6, 172.0, 157.3, 149.4, 136.6, 123.3, 121.6, 67.9, 63.7, 54.7, 48.9, 33.9, 26.4, 24.2, 13.2. HRMS (ESI): calcd for $C_{16}H_{20}N_4NaO_3$ $[M+Na]^+$ 339.1433, found 339.1441.

3. ^{1}H and ^{13}C NMR spectra of the key and new compounds

2-Amino-*N*-methylpropanamide (S11)

^{1}H NMR (600 MHz, $CDCl_3$/TMS)

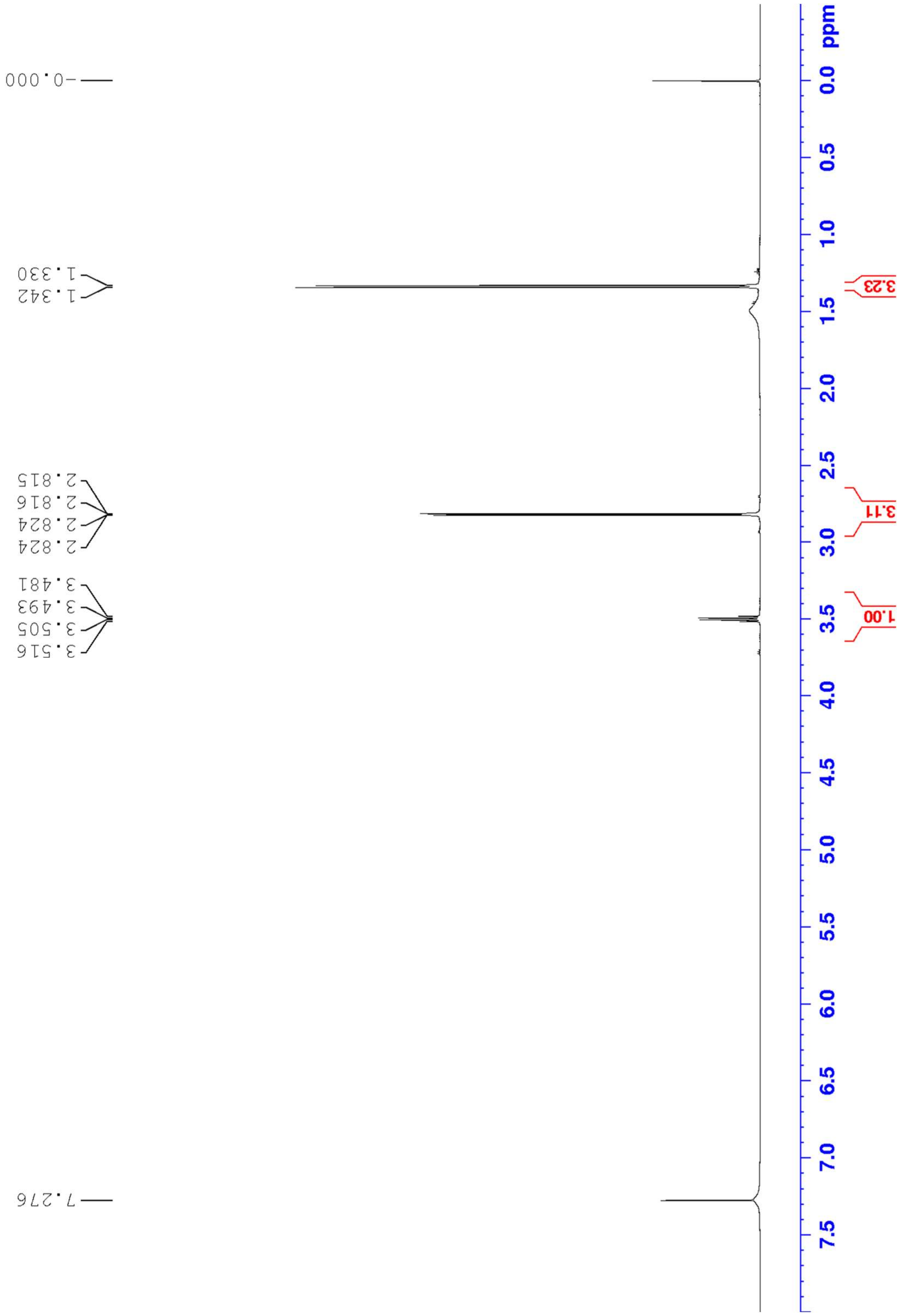

^{13}C NMR (125 MHz, $CDCl_3$)

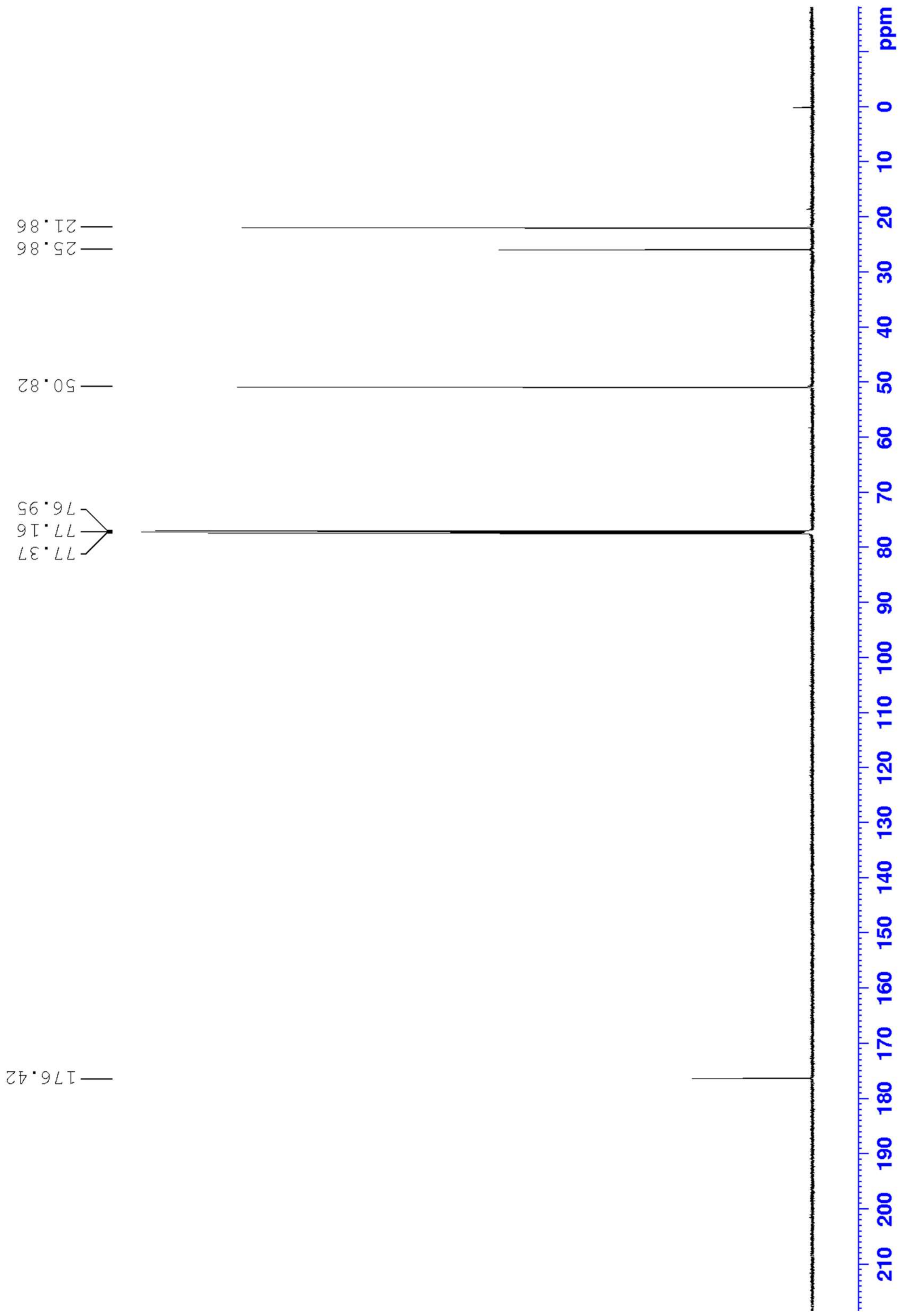
21.86
25.86
50.82
76.95
77.16
77.37
176.42
ppm

(1S*,3R*,3aS*,6aR*)-5-Ethyl-*N*,1-dimethyl-4,6-dioxo-3-(pyridin-2-yl)octahydropyrrolo[3,4-c]pyrrole-1-carboxamide (5)

^{1}H NMR (600 MHz, $CDCl_3$/TMS)

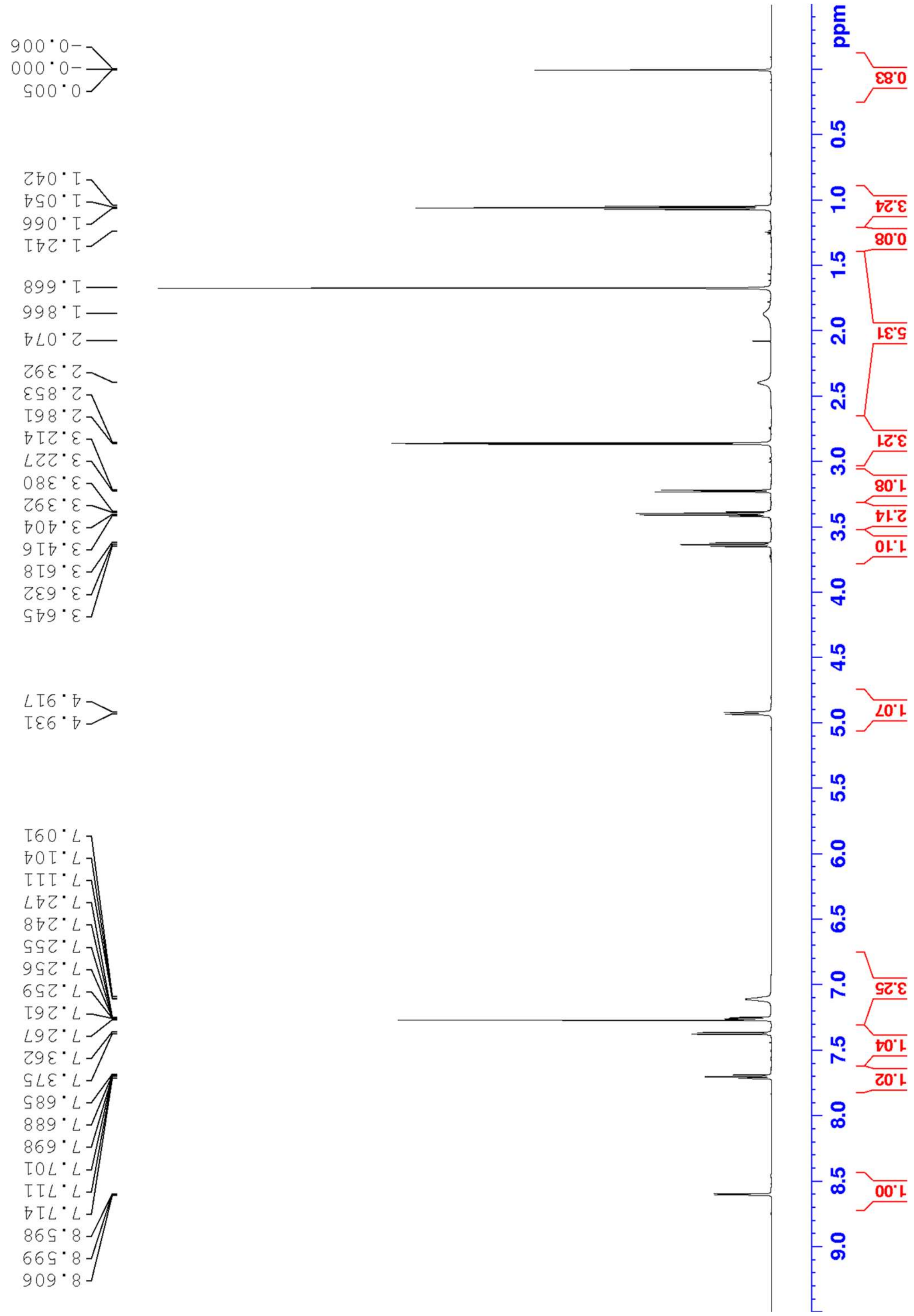

^{13}C NMR (125 MHz, $CDCl_3$)

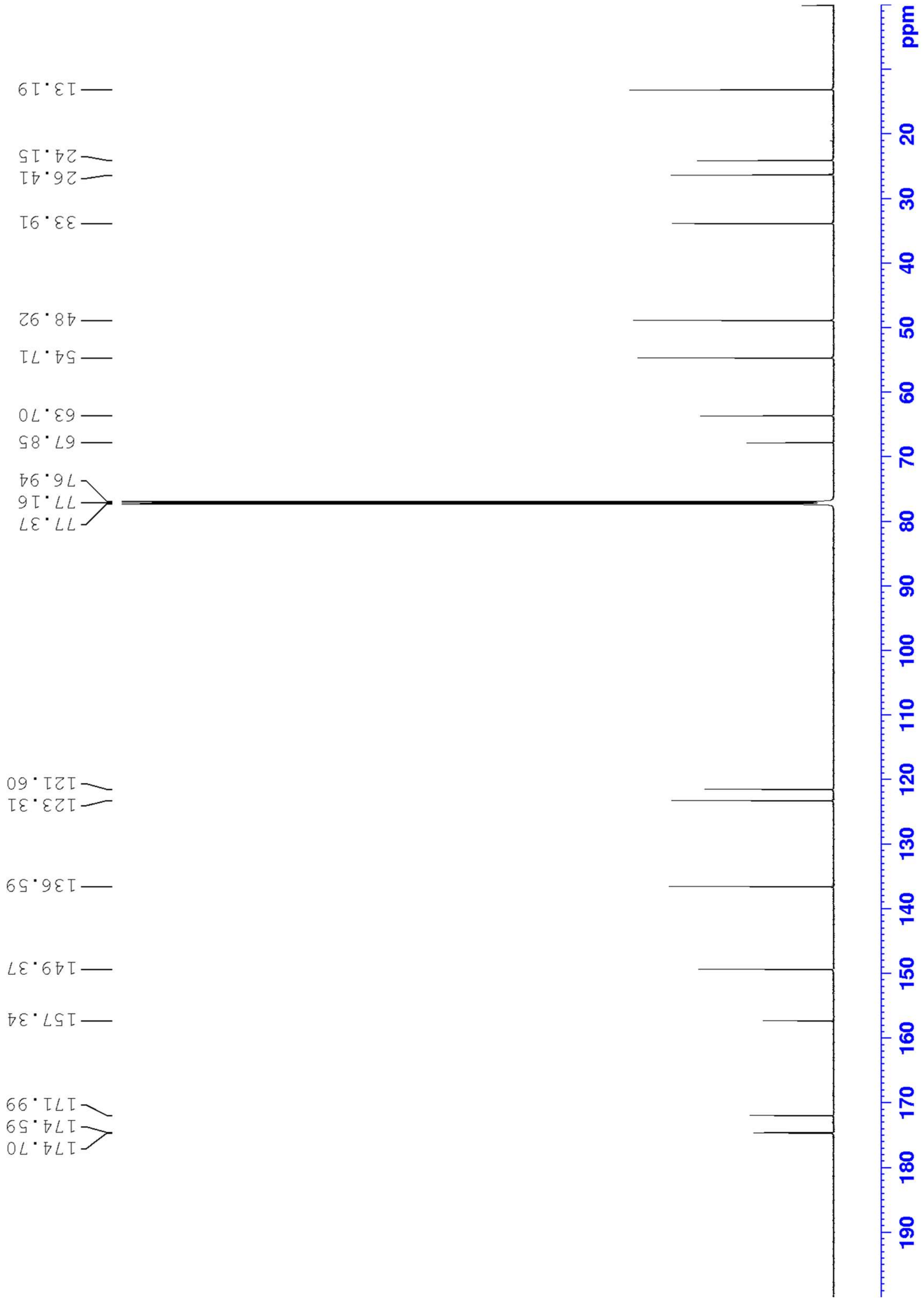

Modified peptide T 4aa (Rt: 8.5 min)

^{1}H NMR (600 MHz, DMSO-d_6)

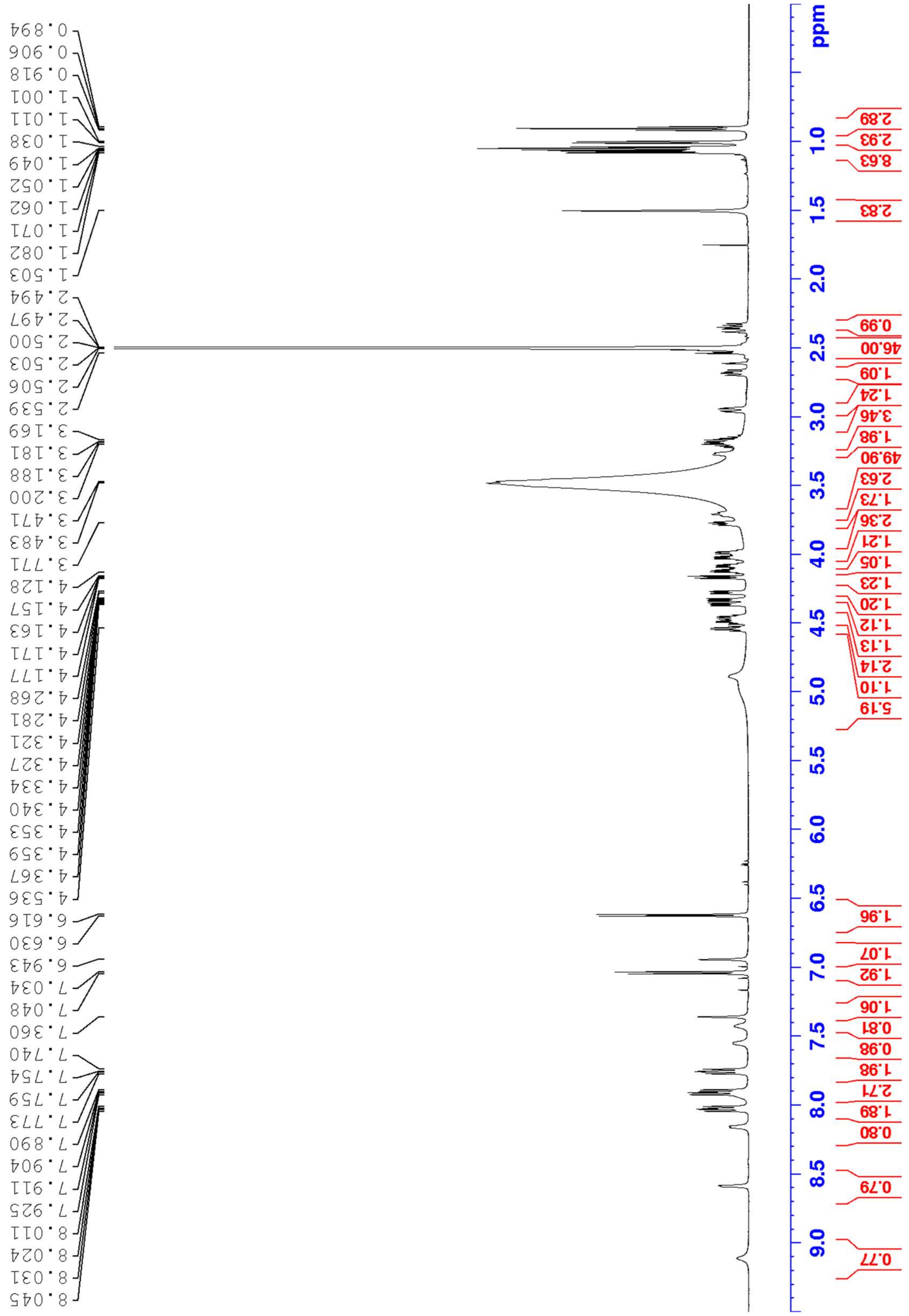

^{13}C NMR (125 MHz, DMSO-d_6)

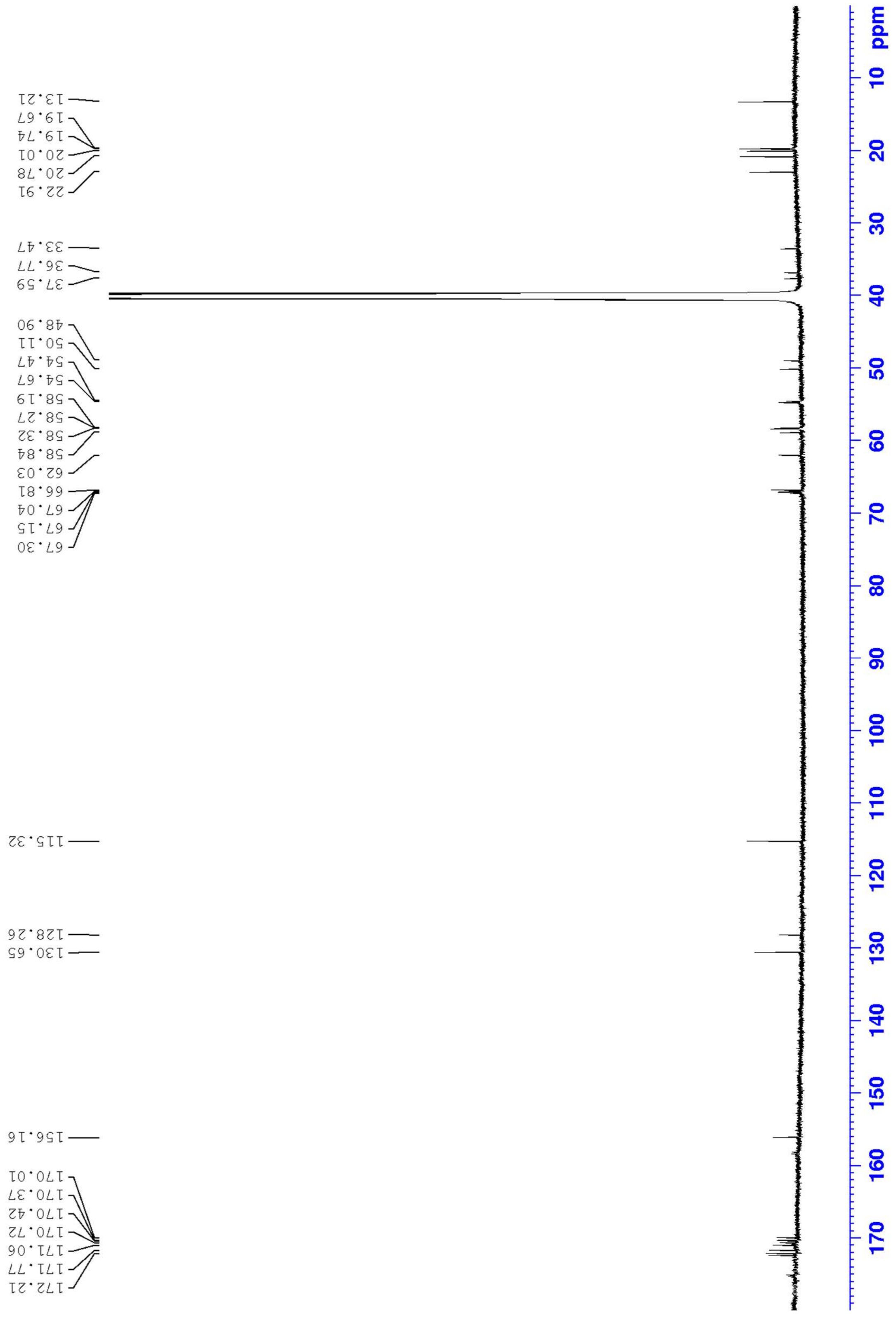
13.21
19.67
19.74
20.01
20.78
22.91
33.47
36.77
37.59
48.90
50.11
54.47
54.67
58.19
58.27
58.32
58.84
62.03
66.81
67.04
67.15
67.30
115.32
128.26
130.65
156.16
170.01
170.37
170.42
170.72
171.06
171.77
172.21
ppm

Modified peptide T 4aa (Rt: 10.2 min)

^{1}H NMR (600 MHz, DMSO-d$_6$)

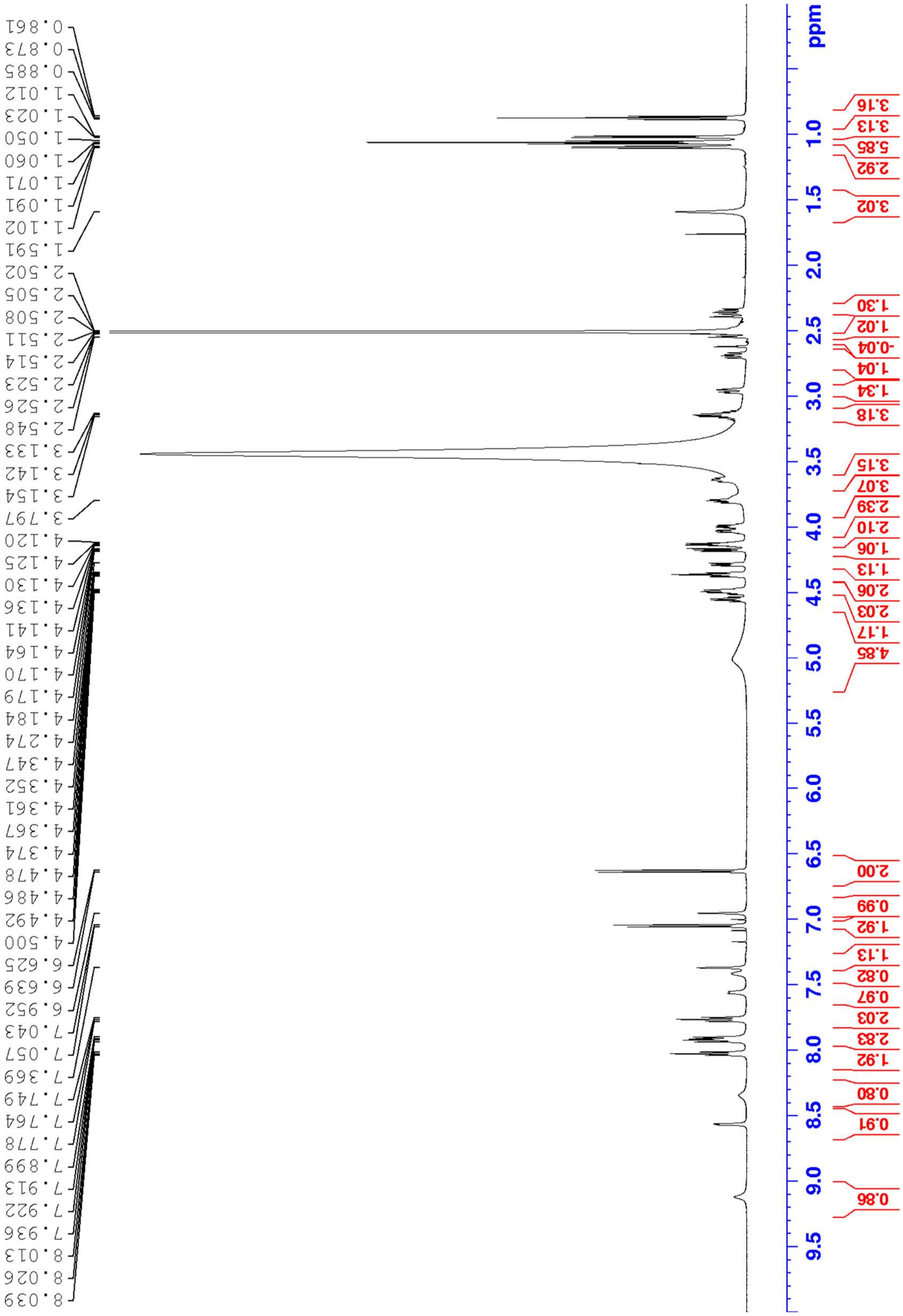

^{13}C NMR (125 MHz, DMSO-d$_6$)

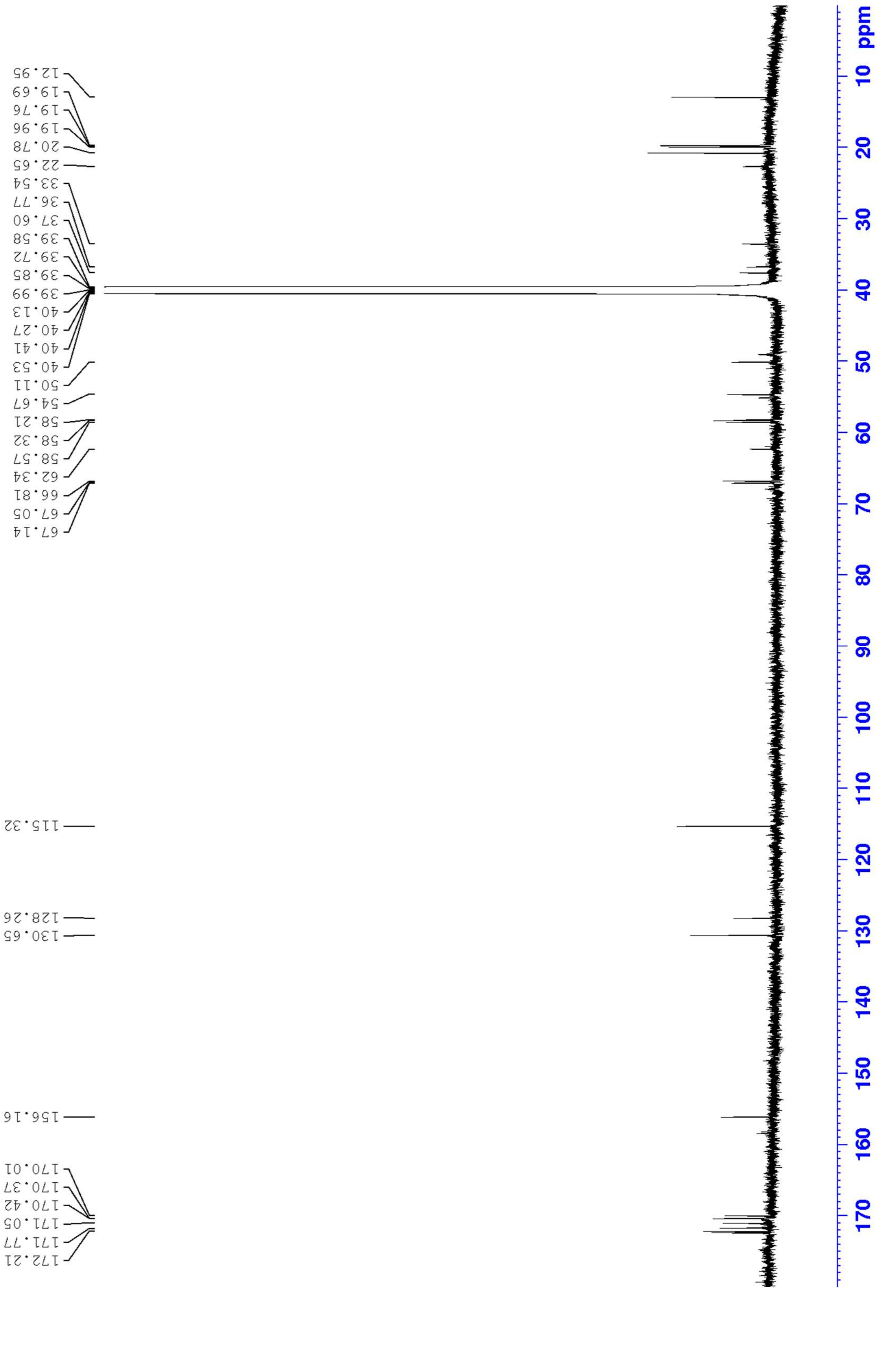

12.95
19.69
19.76
19.96
20.78
22.65
33.54
36.77
37.60
39.58
39.72
39.85
39.99
40.13
40.27
40.41
40.53
50.11
54.67
58.21
58.32
58.57
62.34
66.81
67.05
67.14
115.32
128.26
130.65
156.16
170.01
170.37
170.42
171.05
171.77
172.21
ppm
10
20
30
40
50
60
70
80
90
100
110
120
130
140
150
160
170

4. References

[1] O. V. Dolomanov, L. J. Bourhis, R. J. Gildea, J. a. K. Howard, H. Puschmann, *J. Appl. Crystallogr.* **2009**, *42*, 339–341.
[2] G. M. Sheldrick, *Acta Crystallogr. Sect. Found. Adv.* **2015**, *71*, 3–8.
[3] G. M. Sheldrick, *Acta Crystallogr. Sect. C Struct. Chem.* **2015**, *71*, 3–8.
[4] Y. Okamoto, K. Taguchi, S. Imoto, V. T. Giam Chuang, K. Yamasaki, M. Otagiri, *J. Drug Deliv. Sci. Technol.* **2020**, *55*, 101381.
[5] T. Morack, C. Mück-Lichtenfeld, R. Gilmour, *Angew. Chem. Int. Ed.* **2019**, *58*, 1208–1212.
